# Radical-mediated dehydrative preparation of cyclic imides using (NH_4_)_2_S_2_O_8_–DMSO: application to the synthesis of vernakalant

**DOI:** 10.3762/bjoc.11.113

**Published:** 2015-06-12

**Authors:** Dnyaneshwar N Garad, Subhash D Tanpure, Santosh B Mhaske

**Affiliations:** 1 CSIR-National Chemical Laboratory, Division of Organic Chemistry, Pune 411 008, India

**Keywords:** APS-DMSO, imides, practical synthesis, radical-mediated, vernakalant

## Abstract

Ammonium persulfate–dimethyl sulfoxide (APS–DMSO) has been developed as an efficient and new dehydrating reagent for a convenient one-pot process for the synthesis of miscellaneous cyclic imides in high yields starting from readily available primary amines and cyclic anhydrides. A plausible radical mechanism involving DMSO has been proposed. The application of this facile one-pot imide forming process has been demonstrated for a practical synthesis of vernakalant.

## Introduction

**Figure 1:**
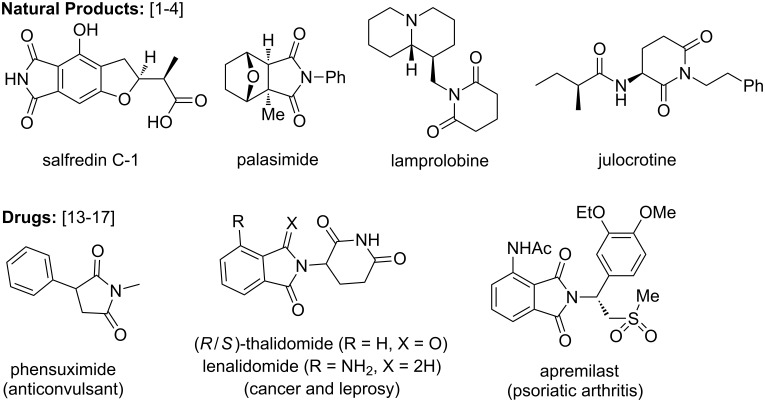
Natural products and drugs featuring imide core.

Cyclic imides are privileged pharmacophores and important building blocks for the synthesis of natural products, drugs, agrochemicals, advanced materials and polymers. Migrastatin, lamprolobine, julocrotine, cladoniamide A, palasimide and salfredin C-1 are few of the important natural products having the imide motif [1–4]. Imides have been extensively used in the synthesis of several bioactive natural products [5–12]. Various drugs such as lurasidone, phensuximide, buspirone, (*R*/*S*)-thalidomide, lenalidomide and apremilast contain cyclic imide moieties and possess a wide range of biological properties (Figure 1) [13–17]. Cyclic imides have found immense applications in agrochemicals such as chlorophthalim (herbicide), captan (fungicide), flumipropyn (herbicide), flumioxazin (herbicide), procymidone (pesticide) and cinidon-ethyl (herbicide) [18–19]. Imides are the backbones of several commercially available high-performance polymers [20–21] and many other advanced materials [22–25].

The most commonly used method to construct the imide functionality is to react the corresponding primary amines with cyclic anhydrides to form amic acids, which are then cyclized using acetic anhydride and sodium acetate in a separate step [26]. Hexamethyldisilazane–zinc chloride has also become one of the most widely used dehydrating reagent for imide synthesis [27]. Several dehydrating conditions such as heating in ionic liquid at 140 °C, heating at 150–180 °C under microwave in various solvents, reaction with *N*,*N'*-disuccinimidyl oxalate followed by heating in trichloroethylene with 4-(dimethylamino)pyridine, Nb_2_O_5_, hydrothermal cyclization, H_2_SO_4_ in acetic acid, PEG-600, silica supported TaCl_5_, esterification using inorganic base and alkylating agent followed by heating in the presence of tetrabutylammonium bromide, diphenyl 2-oxo-3-oxazolinylphosphonate and Et_3_N as well as several other conditions have been reported in the literature [28–45]. Thermal cyclization processes are still the preferred way for the synthesis of polyimides from the intermediate polyamic acid, which is heated in high boiling solvents (>200 °C) with continuous removal of water by distillation [46]. Imide synthesis methodologies starting from different types of starting materials also have been reported [47–54]. However, low yields, use of acidic conditions, lack of generality, use of expensive and toxic metal catalysts or reagents, formation of hazardous byproducts, difficult to access starting materials, azeotropoic removal of water, column purification, harsh reaction conditions, longer reaction time and lack of scalability are some of the drawbacks of prior methods. The development of improved processes to such an important building block is an area of persistent interest [28–54].

## Results and Discussion

**Scheme 1:**
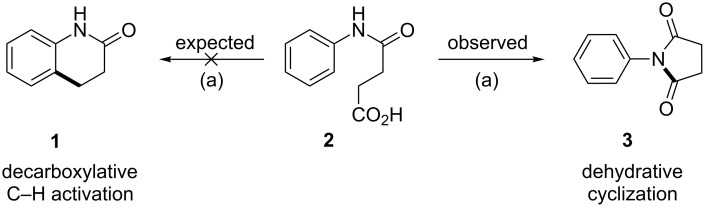
Attempted methodology and its outcome (reaction conditions: (a) Pd(OAc)_2_ (10 mol %), ammonium persulfate (APS) (2 equiv), 1,4-dioxane (0.1 M), DMSO (5% v/v), 100 °C, 3 h in a Schlenk tube).

While working on the development of a palladium-catalyzed decarboxylative C–H activation methodology to access the important core structure dihydroquinolone **1** from succinanilic acid (**2**), we observed dehydrative cyclization to furnish *N*-phenylsuccinimide (**3**) instead of the expected product **1** (Scheme 1).

We were curious to find the actual reagents responsible for this serendipitous facile transformation. We found that the cyclization reaction works in the absence of a palladium catalyst and without the need of inert atmosphere or Schlenk tube, however, the presence of both APS and DMSO was necessary. Optimization of the protocol using various permutations and combinations provided the ideal reaction conditions for imide synthesis (Table 1, entry 9). To the best of our knowledge, although, APS is being used in several commercial applications [55] and in organic [56–60] as well as polymer [55] chemistry as an oxidizing agent, it has been never reported to work as a dehydrating reagent via a radical pathway.

**Table 1:** Optimization studies^a^.

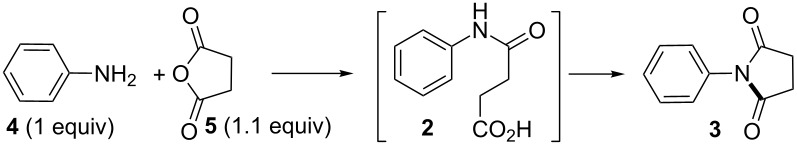				

Entry	Oxidant/DMSO (equiv)	Solvent (3 mL)	Temp/Time^b^ (°C/h)	Yield (%)^c^

1	APS(2)/ 5% v/v	dioxane	100/3	72
2	APS (2)/ -	dioxane	100/3	10
3	– /5% v/v	dioxane	100/4	–
4	APS (1.2)/ 4	dioxane	100/6	40
5	APS (1.5)/ 4	dioxane	100/6	75
6	APS (2)/ 4	dioxane	100/6	94
7	APS (2)/ 1	dioxane	100/8	72
8	APS (2)/ 1.5	dioxane	100/8	78
9	APS (2)/2	dioxane	100/6	93
10	K_2_S_2_O_8_ (1.2)/ 2	dioxane	100/6	15
11	APS (2)/ excess	–	25/4	–
12	APS (2)/ 2	toluene	111/7	67
13	APS (2)/ -	water	100/6	–
14	APS (2)/2	water	100/6	–

^a^All reactions were performed on 60 mg scale of amine **4** under air atmosphere in a round bottom flask equipped with a water condenser; ^b^time required for the radical cyclization step; ^c^isolated yields.

In this context, reported herein is a convenient one-pot process for the preparation of structurally diverse cyclic imides starting from readily available primary amines and cyclic anhydrides using APS–DMSO as an efficient and novel dehydrating reagent and its application to a drug synthesis.

The scope of the developed protocol was studied on varyingly substituted aliphatic/aromatic primary amines and saturated/unsaturated cyclic anhydrides. The generalization of the protocol was first studied on succinic anhydrides and various aromatic amines (Table 2, entries 1–10). The reaction works well with aniline, alkyl-substituted aniline and aniline with electron donating or withdrawing substituents at various positions of the aromatic ring (Table 2, entries 1–5). The reaction performed equally well on 1 g scale (Table 2, entry 3). The steric hindrance or electronic factors did not show much effect on the yield of the reaction. Anilines having halogen substituents at various positions of the aromatic ring furnished the corresponding succinimides in high yields (Table 2, entries 6–9). We were expecting some interference by the iodine in the ortho-position due to a probable formation of a radical; however, we did not observe such effect (Table 2, entry 9). The polyaromatic amine 2-aminoanthracene also reacted smoothly under the optimized conditions (Table 2, entry 10). We studied the effect of substituted succinic anhydrides and observed that mono- and di-substituted succinic anhydride provides the corresponding succinimides in excellent yield (Table 2, entries 11 and 12). Interestingly, the *N*-phenyl analogue of Captan, a commercially used fungicide could be synthesized in excellent yield (Table 2, entry 12) [18–19].

**Table 2:** Imides from substituted/unsubstituted aromatic amines and succinic anhydrides^a^.

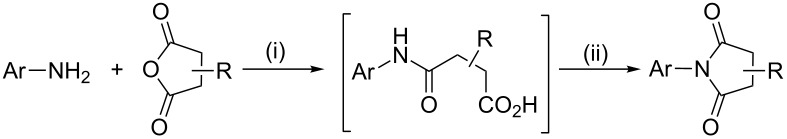			

Entry	Product	Time^b^	Yield^c^

1	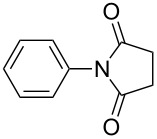	6 h	93%
2	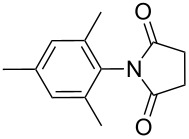	3 h	90%
3	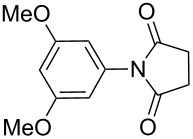	5 h	90% 92%^d^
4	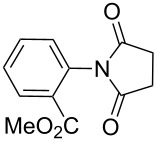	8 h	81%
5	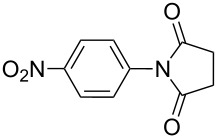	9 h	93%
6	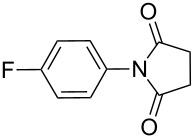	6 h	92%
7	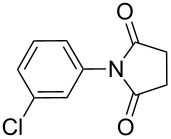	7 h	95%
8	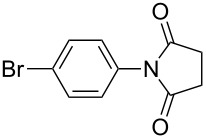	6 h	91%
9	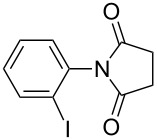	7 h	89%
10	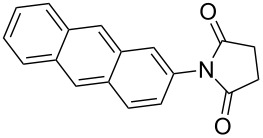	7 h	76%
11	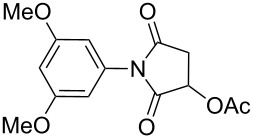	9 h	94%
12	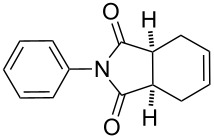	4 h	97%

^a^Reaction conditions: (i) amine (1 equiv, 60 mg scale), anhydride (1.1 equiv), 1,4-dioxane (0.1 M), 25/100 °C, 10 min–12 h; (ii) APS (2 equiv), DMSO (2 equiv), 100 °C; ^b^time required for the radical cyclization step; ^c^isolated yields; ^d^yield for the reaction using 1 g of amine.

The reaction of aromatic amines with unsaturated anhydrides to form maleimides was investigated (Table 3). Aniline, *p*-toluidine and *p*-cyanoaniline furnished the corresponding maleimides from maleic anhydride (Table 3, entries 1–3). 4,4’-Oxydianiline and maleic anhydride also reacted well to provide 4,4’-bis(maleimidodiphenyl) ether in excellent yield (Table 3, entry 4). This bismaleimide and its analogues are important monomers for the synthesis of polymers used in high temperature applications [61–63]. Methyl maleic anhydride and *p*-bromoaniline smoothly provided the corresponding maleimide (Table 3, entry 5). The reaction of *p*-toluidine and phthalic anhydride was very facile and the corresponding phthalimide was obtained in very good yield (Table 3, entry 6).

**Table 3:** Imides from substituted/unsubstituted aromatic amines and unsaturated anhydrides^a^.

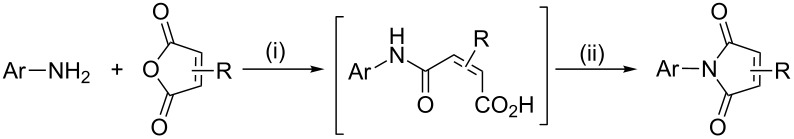			

Entry	Product	Time^b^	Yield^c^

1	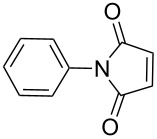	7 h	84%
2	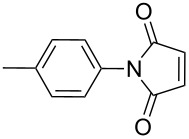	10 h	87%
3	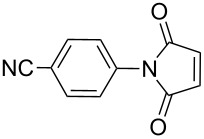	7 h	94%
4	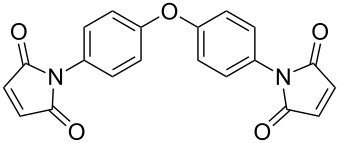	7 h	96%
5	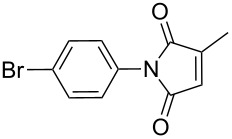	6 h	80%
6	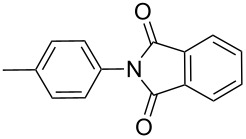	3 h	90%

^a^Reaction conditions: (i) amine (1 equiv, 60 mg scale), anhydride (1.1 equiv), 1,4-dioxane (0.1 M), 25/100 °C, 15 min–24 h; (ii) APS (2 equiv), DMSO (2 equiv), 100 °C; ^b^time required for the radical cyclization step; ^c^isolated yields.

Furthermore, the scope of the protocol to obtain imides from aliphatic amines and saturated anhydride was explored (Table 4). Primary aliphatic amines with short and long alkyl chains were treated with succinic anhydride and they were found to give succinimides in good to excellent yields (Table 4, entries 1–3). Interestingly, the use of secondary and tertiary aliphatic amines also worked equally well (Table 4, entries 4 and 5). Benzylamine was reacted with succinic anhydride and diacetoxysuccinic anhydride to obtain the respective succinimides in high yields (Table 4, entries 6 and 7). The formation of imide from benzylamine and glutaric anhydride worked smoothly (Table 4, entry 8).

**Table 4:** Imides from aliphatic amines and saturated anhydrides^a^.

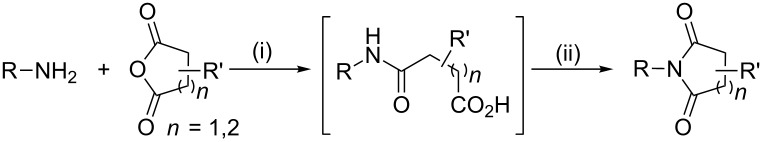			

Entry	Product	Time^b^	Yield^c^

1	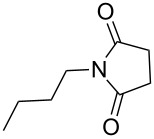	7 h	85%
2	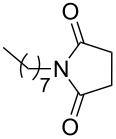	6 h	99%
3	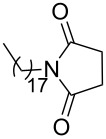	7 h	99%
4	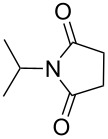	7 h	75%
5	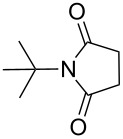	7 h	65%
6	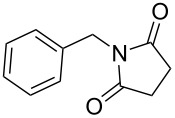	7 h	95%
7	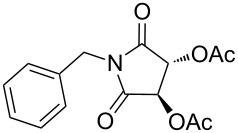	8 h	85%
8	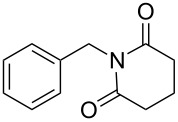	4 h	98%

^a^Reaction conditions: (i) amine (1 equiv, 60 mg scale), anhydride (1.1 equiv), 1,4-dioxane (0.1 M), 25/100 °C, 5 min–1 h; (ii) APS (2 equiv), DMSO (2 equiv), 100 °C; ^b^time required for the radical cyclization step; ^c^isolated yields.

Our protocol worked efficiently with all types of amines and anhydrides (Tables 2–4), but unfortunately it could not be applied successfully to the synthesis of imides from the combination of aliphatic amines and unsaturated anhydrides. Plausibly, the intermediate amic acid in these cases may be prone to decarboxylation [57] and radical polymerization similar to the acrylamide polymerization using APS as a radical initiator [55].

**Scheme 2:**
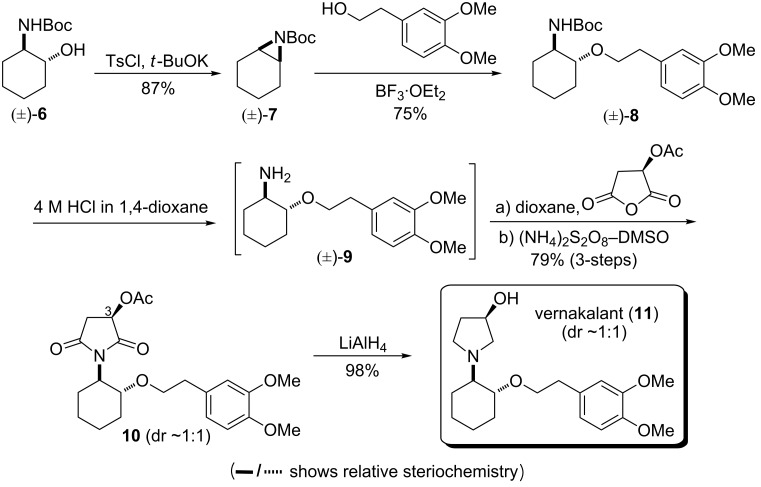
A practical synthesis of vernakalant (**11**).

Encouraged by this elegant transformation (Tables 2–4), we planned to explore our imide forming protocol to the synthesis of the drug vernakalant (**11**). It was discovered by Cardiome/Astellas Pharma Inc. and later developed as a novel antiarrhythmic agent for the treatment of atrial fibrillation (cardiac arrhythmia leading to strokes) in collaboration with Merck & Co., Inc. Its intravenous formulation has been approved as a drug by the European agency (EMEA) under the trade name Brinavess (Cardiome/Merck) [64]. Few synthetic routes to vernakalant (**11**) and its intermediates have been reported in the literature [64]. Our plan was to devise a concise and practical synthetic route. The planned synthetic strategy is illustrated in Scheme 2.

The synthesis began from Boc-protected *trans*-amino alcohol **6** [65]. The two-step sequence – tosylation of the secondary alcohol **6** followed by elimination to form the Boc- protected aziridine **7** – was optimized in a one-pot procedure using *t*-BuOK in THF. Nucleophilic ring opening of the aziridine **7** by homoveratryl alcohol in the presence of a catalytic amount of the Lewis acid BF_3_·OEt_2_ furnished compound **8**. The -NHBoc was deprotected using 4 M HCl in 1,4-dioxane. The free amine **9** obtained after neutralization was directly subjected to the developed protocol, wherein the solution of (*R*)-acetoxy succinic anhydride and the amine **9** was stirred at room temperature for 30 min followed by the addition of APS–DMSO and further heating the reaction mixture at 100 °C for 8 h to give succinimide **10** without any racemization at the C-3 centre. Finally, reduction of the imide and deprotection of the acetyl moiety was done in a single step by LiAlH_4_ to obtain vernakalant (**11**) as the free base. The synthesis was completed in 5 steps with 51% overall yield.

**Figure 2:**
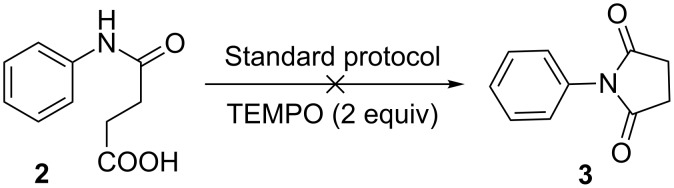
Radical trapping experiment.

A preliminary mechanistic study revealed that the transformation developed herein may be proceeding via a radical pathway. The reaction was conducted with the substrate **2** using our standard protocol in the presence of TEMPO (Figure 2), wherein complete inhibition of the reaction was observed, thus indicating involvement of radical intermediates [57]. Similarly, involvement of DMSO in this transformation was also confirmed by the fact that the reaction does not work well in its absence (Table 1, entry 2). Based on the above observations and some additional studies using GC and ^1^H NMR analysis a plausible reaction mechanism of our imide formation protocol has been depicted in Supporting Information File 1. However, we believe that a more concrete study is necessary to find out the actual mechanism.

## Conclusion

In conclusion, we have developed a novel and efficient protocol “APS–DMSO” for the synthesis of cyclic imides. The scope of the developed protocol is wide and pure products could be obtained without column chromatographic purification, which makes it a commercially sustainable process. A practical synthesis of the drug vernakalant has been achieved using our one-pot imide forming process as one of the important steps. Currently, we are exploring the application of this newly developed protocol for the synthesis of other hetereocyclic compounds, natural products, drugs and polyimides.

## Supporting Information

File 1 Experimental details, characterization data, copies of NMR spectra of all compounds and the details of mechanistic studies.
