# Supplementary material for: Radical-mediated dehydrative preparation of cyclic imides using (NH4)2S2O8–DMSO: application to the synthesis of vernakalant
Source: Beilstein J Org Chem. 2015 Jun 12;11:1008–16. doi: 10.3762/bjoc.11.113 (PMC4505096; doi:10.3762/bjoc.11.113)
Supplement: File 1 — Experimental details, characterization data, copies of NMR spectra of all compounds and the details of mechanistic studies. [file Beilstein_J_Org_Chem-11-1008-s001.pdf]

Supporting Information

for

Radical-mediated dehydrative preparation of cyclic imides using $(NH_4)_2S_2O_8$–DMSO: application to the synthesis of vernakalant

Dnyaneshwar N. Garad, Subhash D. Tanpure and Santosh B. Mhaske*

Address: CSIR-National Chemical Laboratory, Division of Organic Chemistry, Pune 411 008, India

Email: Santosh B. Mhaske* - sb.mhaske@ncl.res.in

*Corresponding author

Experimental details, characterization data, copies of NMR spectra of all compounds and the details of mechanistic studies.

Sr. No.	Index	Pages
1	General information	S2
2	Experimental procedures	S3–S4
3	Characterization data of compounds	S5–S17
4	Mechanistic study	S18–S20
5	Experimental procedures for the synthesis of Vernakalant	S21–S25
6	References	S26–S27
7	Copies of ^{1}H, ^{13}C, DEPT spectra	S28–S117

1. General information:

All reagents and solvents were used as received from commercial sources unless otherwise noted. All experiments were carried out under air atmosphere. 2-Acetoxy and 2,3-diacetoxy succinic anhydrides were synthesized by known procedures [1] and the rest of the anhydrides and amines were purchased from commercial sources. Pre-coated plates (silica gel 60 PF254, 0.25 mm or 0.5 mm) were utilized for thin layer chromatography (TLC). Column chromatographic purifications were carried out on flash silica-gel (240–400 mesh) using petroleum ether and ethyl acetate as eluents. The ^{1}H, ^{13}C NMR spectra were recorded on 200/400/500 MHz, and 50/100/125 MHz NMR spectrometers, respectively in $CDCl_3$/CD_3OD. Chemical shifts were reported as δ values from standard peaks. Melting points were recorded on a Buchi instrument. Mass spectra were taken on LC–MS (ESI) mass spectrometer and GC–MS mass spectrometer. HRMS for new compounds were scanned at NCL, Pune.

2. Experimental procedures:

General experimental procedure for the synthesis of imides:

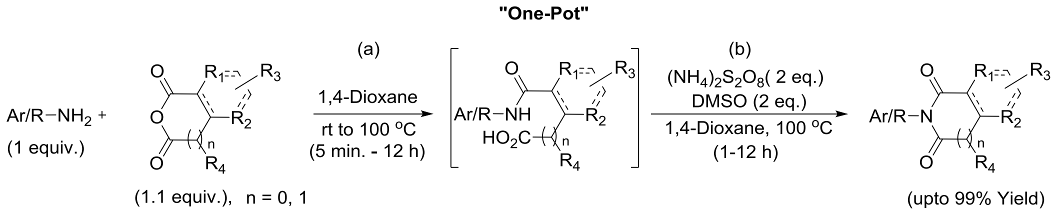

A solution of amine (1 equiv) and anhydride (1.1 equiv) in 1,4-dioxane was stirred at room temperature (or 100 °C if necessary) in a two neck round bottom flask equipped with a water condenser. As soon as all the amine converts to the corresponding amic acid (monitored by TLC), ammonium persulfate [$(NH_4)_2S_2O_8$] (2 equiv) and DMSO (2 equiv) were added and the reaction mixture was heated to 100 °C. Heating was continued at the same temperature until completion (3–10 h) of the reaction. After completion, the reaction mixture was filtered through a cotton plug and dioxane was removed under vacuo. The residue was dissolved in ethyl acetate and washed with dilute HCl, saturated aqueous $NaHCO_3$ and brine. The organic layer was dried over anhydrous Na_2SO_4 and evaporated under vacuo to furnish the corresponding imides in good to excellent yields with more than 98% purity (GC, NMR).

Large scale experiment: Table 2, entry 3

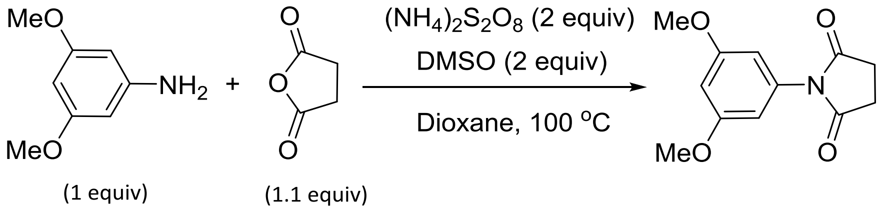

In a two neck round bottom flask equipped with water condenser, a solution of 3,5-dimethoxy aniline (1 g, 6.52 mmol) and succinic anhydride (718.61 mg, 7.18 mmol) in dioxane were stirred at 100 °C for 10 min. Ammonium persulfate (2.98 g, 13.05 mmol) and DMSO (0.93 mL, 13.05 mmol) were added to the reaction mixture and the heating was continued for another 5 h at 100 °C. Thereafter the reaction mixture was filtered through a cotton plug and dioxane was removed under vacuo. The crude product was dissolved in ethyl acetate and washed with dilute HCl, saturated aqueous $NaHCO_3$ and brine. The organic layer was dried over anhydrous Na_2SO_4 and the solvent was evaporated under vacuo to afford *N*-(3,5-dimethoxy)phenyl succinimide (Table 2, entry 3; 1.41 g, 92% yield) in pure form as a brown solid.

3. Characterization data of compounds:

All reactions were performed on 60 mg scale of amines. Representative large scale experiment was performed on 1 g scale of 3,5-dimethoxyaniline.

1-Phenylpyrrolidine-2,5-dione: Table 2, entry 1 [2]

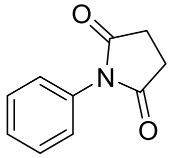

Reaction time: Step (a) at 100 °C, 10 min and step (b), 6 h; R_f: 0.5 (1:1 EtOAc:Pet. ether); white solid, 105 mg, 93% yield; mp = 153-154 °C; ^{1}H NMR (400 MHz, $CDCl_3$) δ 7.47 (t, J = 7.6 Hz, 2H), 7.38 (t, J = 7.6 Hz, 1H), 7.27 (d, J = 7.6 Hz, 2H), 2.87 (s, 4H); ^{13}C NMR (100 MHz, $CDCl_3$) δ 176.1, 131.9, 129.2, 128.6, 126.4, 28.4; ESI-Mass $(M+Li)^+$ 182.

1-Mesitylpyrrolidine-2,5-dione: Table 2, entry 2 [3]

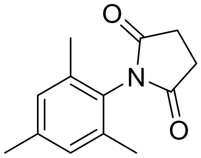

Reaction time: Step (a) at 100 °C, 4 h and step (b), 3 h; R_f: 0.5 (2:3 EtOAc:Pet. ether); white solid, 87 mg, 90% yield; mp = 151-153 °C; ^{1}H NMR (400 MHz, $CDCl_3$) δ 6.90 (s, 2H), 2.86 (s, 4H), 2.23 (s, 3H), 2.00 (s, 6H); ^{13}C NMR (100 MHz, $CDCl_3$) δ 176.1, 139.5, 135.2, 129.4, 127.5, 28.6, 21.1, 17.7; ESI-Mass $(M+H)^+$ 218.

1-(3,5-Dimethoxyphenyl)pyrrolidine-2,5-dione: Table 2, entry 3 [4]

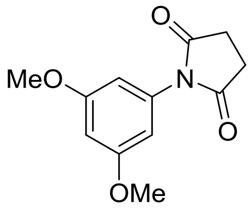

Reaction time: Step (a) at 100 °C, 10 min and step (b), 5 h; R_f: 0.4 (1:1 EtOAc:Pet. ether); brown solid, 83 mg, 90% yield; mp = 133-135 °C; ^{1}H NMR (400 MHz, $CDCl_3$) δ 6.44-6.41 (m, 1H), 6.35-6.33 (m, 2H), 3.71 (s, 6H), 2.81 (s, 4H); ^{13}C NMR (100 MHz, $CDCl_3$) δ 176.0, 161.0, 133.3, 105.0, 101.1, 55.5, 28.4; ESI-Mass $(M+Na)^+$ 258.

Methyl 2-(2,5-dioxopyrrolidin-1-yl)benzoate: Table 2, entry 4 [5]

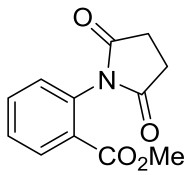

Reaction time: Step (a) at 100 °C, 8 h and step (b), 8 h; R_f: 0.5 (1:1 EtOAc:Pet. ether); yellow oil, 75 mg, 81% yield; ^{1}H NMR (400 MHz, $CDCl_3$) [Mixture of two atropisomers] δ 8.07 (m, 1H), 7.59 (m, 1H), 7.45 (m, 1H), 7.18 (m, 1H), 3.77 (two s, 3H), 2.95-2.75 (m, 4H); ^{13}C NMR (100 MHz, $CDCl_3$) δ 176.7, 164.9, 133.5, 132.6, 131.8, 129.8, 129.4, 127.1, 52.3, 28.8; ESI-Mass $(M+Na)^+$ 256.

1-(4-Nitrophenyl)pyrrolidine-2,5-dione: Table 2, entry 5 [6]

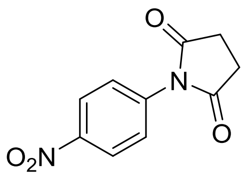

Reaction time: Step (a) in toluene at 100 °C, 1 h and step (b) in dioxane, 9 h; R*f*: 0.6 (1:1 EtOAc:Pet. ether); yellow solid, 89 mg, 93% yield; mp = 208-209 °C; ^{1}H NMR (500 MHz, $CDCl_3$) δ 8.27 (d, J = 9.1 Hz, 2H), 7.53 (d, J = 9.1 Hz, 2H), 2.89 (s, 4H); ^{13}C NMR (100 MHz, $CDCl_3$) δ 175.2, 147.0, 137.4, 126.8, 124.4, 28.4; GC-MS (M^+) 220.

1-(4-Fluorophenyl)pyrrolidine-2,5-dione: Table 2, entry 6 [7]

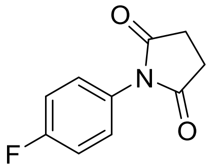

Reaction time: Step (a) at rt, 30 min and step (b), 6 h; R_f: 0.6 (1:1 EtOAc:Pet. ether); brown solid, 96 mg, 92% yield; mp = 175-177 °C; ^{1}H NMR (500 MHz, $CDCl_3$) δ 7.23-7.18 (m, 2H), 7.12-7.07 (m, 2H), 2.82 (s, 4H); ^{13}C NMR (125 MHz, $CDCl_3$) δ 176.0, 162.2 (d, J = 248.9 Hz), 128.3 (d, J = 9.1 Hz), 127.7 (d, J = 2.7 Hz), 116.2 (d, J = 22.7 Hz), 28.3; ESI-Mass $(M+Na)^+$ 216.

1-(3-Chlorophenyl)pyrrolidine-2,5-dione: Table 2, entry 7 [6]

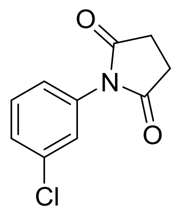

Reaction time: Step (a) at 100 °C, 4 h and step (b), 7 h; R_f: 0.5 (2:3 EtOAc:Pet. ether); brown solid, 94 mg, 95% yield; mp = 107-109 °C; ^{1}H NMR (400 MHz, $CDCl_3$) δ 7.38-7.28 (m, 2H), 7.27 (t, J = 1.7 Hz, 1H), 7.15 (dt, J = 7.3, 1.7 Hz, 1H), 2.83 (s, 4H); ^{13}C NMR (100 MHz, $CDCl_3$) δ 175.7, 134.7, 132.9, 130.1, 128.8, 126.7, 124.6, 28.4; GC-MS (M^+) 209.

1-(4-Bromophenyl)pyrrolidine-2,5-dione: Table 2, entry 8 [6]

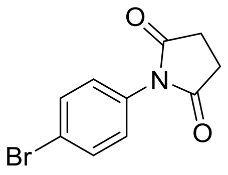

Reaction time: Step (a) at 100 °C, 3 h and step (b), 6 h; R_f: 0.6 (2:3 EtOAc:Pet. ether); off-white solid, 81 mg, 91% yield; mp = 171-172 °C; ^{1}H NMR (400 MHz, $CDCl_3$) δ 7.53 (d, J = 8.6 Hz, 2H), 7.13 (d, J = 8.6 Hz, 2H), 2.82 (s, 4H); ^{13}C NMR (100 MHz, $CDCl_3$) δ 175.7, 132.3, 130.8, 127.9, 122.4, 28.4; GC-MS (M^+) 253:255 (1:1).

1-(2-Iodophenyl)pyrrolidine-2,5-dione: Table 2, entry 9 [8]

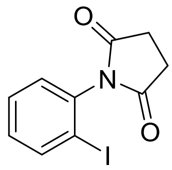

Reaction time: Step (a) at 100 °C, 12 h and step (b), 7 h; R_f: 0.5 (1:1 EtOAc:Pet. ether); off-white solid, 73 mg, 89% yield; mp = 129-130 °C; ^{1}H NMR (400 MHz, $CDCl_3$) [Mixture of two atropisomers] δ 7.88 (d, J = 7.8, Hz, 1H), 7.40 (m, 1H), 7.18-7.06 (m, 2H), 3.05-2.79 (m, 4H); ^{13}C NMR (100 MHz, $CDCl_3$) δ 175.3, 139.9, 135.4, 131.1, 129.5, 129.4, 97.9, 28.8; ESI-Mass $(M+Na)^+$ 324.

1-(Anthracen-2-yl)pyrrolidine-2,5-dione: Table 2, entry 10

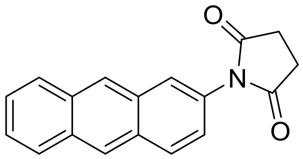

Reaction time: Step (a) at 100 °C, 4.5 h and step (b), 7 h; R_f: 0.4 (2:3 EtOAc:Pet. ether); brown solid, 65 mg, 76% yield; mp = 262-264 °C; ^{1}H NMR (400 MHz, $CDCl_3$) δ 8.37 (s, 2H), 8.01 (d, J = 9.1 Hz, 1H), 7.98-7.91 (m, 2H), 7.90 (s, 1H), 7.46-7.39 (m, 2H), 7.27 (dd, J = 9.1, 1.7 Hz, 1H), 2.89 (s, 4H); ^{13}C NMR (100 MHz, $CDCl_3$) δ 176.3, 132.2, 132.0, 130.9, 130.6, 129.6, 128.8, 128.2, 128.1, 127.0, 126.4, 125.88, 125.85, 125.79, 123.3, 28.5; HRMS-ESI (m/z) calcd $(C_{18}H_{14}NO_2)^+$ $[M+H]^+$: 276.1025, found: 276.1030.

1-(3,5-Dimethoxyphenyl)-2,5-dioxopyrrolidin-3-yl acetate: Table 2, entry 11

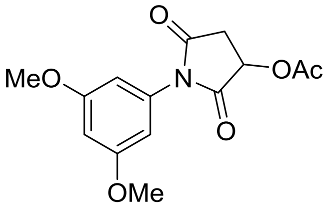

Reaction time: Step (a) at 100 °C, 45 min and step (b), 9 h; R_f: 0.5 (2:3 EtOAc:Pet. ether); white solid, 108 mg, 94% yield; mp = 90-91 °C; ^{1}H NMR (400 MHz, $CDCl_3$) δ 6.44 (t, J = 2.2 Hz, 1H), 6.37 (d, J = 2.2 Hz, 2H), 5.49 (dd, J = 8.8, 4.9 Hz, 1H), 3.72 (s, 6H), 3.25 (dd, J = 18.4, 8.8 Hz, 1H), 2.78 (dd, J = 18.4, 4.9 Hz, 1H), 2.13 (s, 3H); ^{13}C NMR (100 MHz, $CDCl_3$) δ 172.3, 172.1, 169.9, 161.1, 132.7, 104.8, 101.4, 67.6, 55.5, 35.8, 20.5; HRMS-ESI (m/z) calcd $(C_{14}H_{16}NO_6)^+$ $[M+H]^+$: 294.0978, found: 294.0979.

***cis*-2-Phenyl-3a,4,7,7a-tetrahydro-1*H*-isoindole-1,3(2*H*)-dione:** Table 2, entry 12 [9]

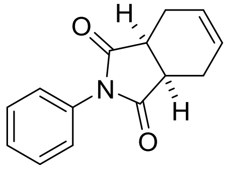

Reaction time: Step (a) at rt, 2 h and step (b), 4 h; R_f: 0.5 (1:4 EtOAc:Pet. ether); colorless crystals, 142 mg, 97% yield; mp = 114-115 °C; ^{1}H NMR (400 MHz, $CDCl_3$) δ 7.43-7.35 (m, 2H), 7.34-7.27 (m, 1H), 7.21-7.11 (m, 2H), 5.96-5.87 (m, 2H), 3.23-3.15 (m, 2H), 2.70-2.59 (m, 2H), 2.31-2.19 (m, 2H); ^{13}C NMR (100 MHz, $CDCl_3$) δ 179.2, 132.0, 129.1, 128.5, 127.8, 126.4, 39.2, 23.7; GC-MS (M^+) 227.

1-Phenyl-1*H*-pyrrole-2,5-dione: Table 3, entry 1 [10]

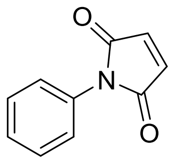

Reaction time: Step (a) at rt, 1 h and step (b), 7 h; R_f: 0.5 (1:4 EtOAc:Pet. ether); yellowish- grey solid, 94 mg, 84% yield; mp = 83-84 °C; ^{1}H NMR (400 MHz, $CDCl_3$) δ 7.40 (t, J = 7.3 Hz, 2H), 7.33-7.24 (m, 3H), 6.77 (s, 2H); ^{13}C NMR (100 MHz, $CDCl_3$) δ 169.5, 134.2, 131.2, 129.1, 127.9, 126.1; GC-MS (M^+) 173.

1-(*p*-Tolyl)-1*H*-pyrrole-2,5-dione: Table 3, entry 2 [11]

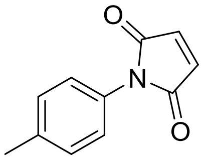

Reaction time: Step (a) at 100 °C, 45 min and step (b), 10 h; R_f: 0.5 (1:4 EtOAc:Pet. ether); yellow solid, 91 mg, 87% yield; mp = 180-182 °C; ^{1}H NMR (400 MHz, $CDCl_3$) δ 7.20 (d, J = 8.2 Hz, 2H), 7.13 (d, J = 8.2 Hz, 2H), 6.76 (s, 2H), 2.31 (s, 3H); ^{13}C NMR (100 MHz, $CDCl_3$) δ 169.7, 138.1, 134.2, 129.8, 128.5, 126.0, 21.1; GC-MS $(M)^+$ 187.

4-(2,5-Dioxo-2,5-dihydro-1*H*-pyrrol-1-yl)benzonitrile: Table 3, entry 3 [12]

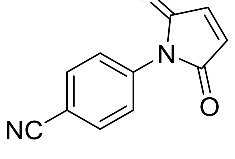

Reaction time: Step (a) at 100 °C, 6 h and step (b), 7 h; R_f: 0.5 (1:2 EtOAc:Pet. ether); brown solid, 95 mg, 94% yield; mp = 129-130 °C; ^{1}H NMR (500 MHz, $CDCl_3$) δ 7.69 (d, J = 8.9 Hz, 2H), 7.53 (d, J = 8.9 Hz, 2H), 6.84 (s, 2H); ^{13}C NMR (125 MHz, $CDCl_3$) δ 168.5, 135.5, 134.5, 133.0, 125.6, 118.1, 111.2; GC-MS $(M)^+$ 198.

1,1'-(Oxybis(4,1-phenylene))bis(1*H*-pyrrole-2,5-dione): Table 3, entry 4 [13]

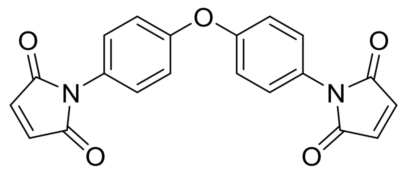

Reaction time: Step (a) at 50 °C, 24 h and step (b), 7 h; R_f: 0.5 (1:1 EtOAc:Pet. ether); brown solid, 104 mg, 96% yield; mp = 159-160 °C; ^{1}H NMR (400 MHz, $CDCl_3$) δ 7.25 (d, J = 9.0 Hz, 4H), 7.05 (d, J = 9.0 Hz, 4H), 6.79 (s, 4H); ^{13}C NMR (100 MHz, $CDCl_3$) δ 169.5, 156.3, 134.2, 127.7, 126.5, 119.5.

1-(4-Bromophenyl)-3-methyl-1*H*-pyrrole-2,5-dione: Table 3, entry 5 [14]

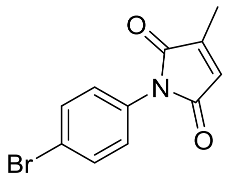

Reaction time: Step (a) at 100 °C, 8 h and step (b), 6 h; R_f: 0.5 (1:4 EtOAc:Pet. ether); white solid, 74 mg, 80% yield; mp = 142-144 °C ^{1}H NMR (500 MHz, $CDCl_3$) δ 7.53-7.48 (m, 2H), 7.21-7.17 (m, 2H), 6.42 (q, J = 1.8 Hz, 1H), 2.11 (d, J = 1.8 Hz, 3H); ^{13}C NMR (125 MHz,

$CDCl_3$) δ 170.2, 169.1, 146.0, 132.2, 130.7, 127.6, 127.2, 121.3, 11.2; GC-MS (M^+) 265:267 (1:1).

2-(*p*-Tolyl)isoindoline-1,3-dione: Table 3, entry 6 [15]

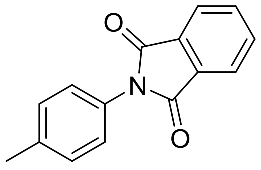

Reaction time: Step (a) at 100 °C, 15 min and step (b), 3 h; R_f: 0.5 (1:4 EtOAc:Pet. ether); white solid, 119 mg, 90% yield; mp = 173-175 °C; ^{1}H NMR (500 MHz, $CDCl_3$) δ 7.87 (dd, J = 5.3, 3.1 Hz, 2H), 7.70 (dd, J = 5.3, 3.1 Hz, 2H), 7.24 (s, 4H); 2.33 (s, 3H); ^{13}C NMR (125 MHz, $CDCl_3$) δ 167.4, 138.2, 134.3, 131.8, 129.8, 128.9, 126.5, 123.7, 21.2; GC-MS (M^+) 237.

1-Butylpyrrolidine-2,5-dione: Table 4, entry 1 [6]

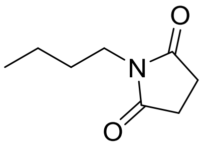

Reaction time: Step (a) at rt, 15 min and step (b), 7 h; R_f: 0.5 (2:3 EtOAc:Pet. ether); colorless oil, 108 mg, 85% yield; ^{1}H NMR (400 MHz, $CDCl_3$) δ 3.44 (t, J = 7.3 Hz, 2H), 2.63 (s, 4H), 1.53-1.42 (m, 2H), 1.20-1.19 (m, 2H), 0.86 (t, J = 7.3 Hz, 3H); ^{13}C NMR (100 MHz, $CDCl_3$) δ 177.3, 38.6, 29.7, 28.1, 20.1, 13.6; GC-MS (M^+) 155.

1-Octylpyrrolidine-2,5-dione: Table 4, entry 2 [16]

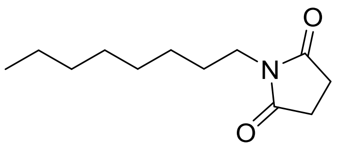

Reaction time: Step (a) at 15 min and step (b), 6 h; R_f: 0.6 (2:3 EtOAc:Pet. ether); semi-solid, 97 mg, 99% yield; ^{1}H NMR (500 MHz, $CDCl_3$) δ 3.42 (t, J = 7.5 Hz, 2H), 2.63 (s, 4H), 1.52-1.44 (m, 2H), 1.26-1.14 (m, 10H), 0.80 (t, J = 6.9 Hz, 3H); ^{13}C NMR (125 MHz, $CDCl_3$) δ 177.3, 38.9, 31.7, 29.1, 28.1 (2C), 27.7, 26.8, 22.6, 14.0; ESI-Mass $(M+Na)^+$ 234.

1-Octadecylpyrrolidine-2,5-dione: Table 4, entry 3 [17]

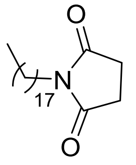

Reaction time: Step (a) at rt, 20 min and step (b), 7 h; R_f: 0.5 (1:2 EtOAc:Pet. ether); white solid, 77 mg, 99% yield; mp = 76-77 °C; ^{1}H NMR (400 MHz, $CDCl_3$) δ 3.42 (t, J = 7.5 Hz, 2H), 2.62 (s, 4H), 1.53-1.40 (m, 2H), 1.26-1.08 (m, 30H), 0.81 (t, J = 6.9 Hz, 3H); ^{13}C NMR (100 MHz, $CDCl_3$) δ 177.3, 38.9, 31.9, 29.7, 29.54, 29.46, 29.3, 29.2, 28.1, 27.7, 26.9, 22.7, 14.1; GC-MS (M^+) 351.

1-Isopropylpyrrolidine-2,5-dione: Table 4, entry 4 [18]

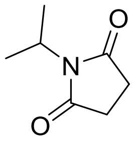

Reaction time: Step (a) at 0 °C, 1 h and step (b), 7 h; R_f: 0.6 (2:3 EtOAc:Pet. ether); dark brown solid, 107 mg, 75% yield; mp = 62-63 °C; ^{1}H NMR (400 MHz, $CDCl_3$) δ 4.32 (septet, J = 6.8 Hz, 1H), 2.57 (s, 4H), 1.32 (d, J = 6.8 Hz, 6H); ^{13}C NMR (100 MHz, $CDCl_3$) δ 177.3, 43.7, 28.1, 19.2; GC-MS (M^+) 141.

1-(*tert*-Butyl)pyrrolidine-2,5-dione: Table 4, entry 5 [18]

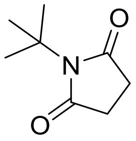

Reaction time: Step (a) at rt, 45 min and step (b), 7 h; R_f: 0.6 (1:2 EtOAc:Pet. ether); yellow solid, 83 mg, 65% yield; mp = 48-49 °C; ^{1}H NMR (500 MHz, $CDCl_3$) δ 2.51 (s, 4H), 1.51 (s, 9H); ^{13}C NMR (125 MHz, $CDCl_3$) δ 178.3, 58.3, 28.6, 28.3; GC-MS (M^+) 155.

1-Benzylpyrrolidine-2,5-dione: Table 4, entry 6 [19]

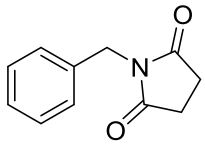

Reaction time: Step (a) at rt, 5 min and step (b), 7 h; R_f: 0.5 (2:3 EtOAc:Pet. ether); white solid, 101 mg, 95% yield; mp = 101-102 °C; ^{1}H NMR (500 MHz, $CDCl_3$) δ 7.34-7.29 (m, 2H), 7.27-

7.18 (m, 3H), 4.58 (s, 2H), 2.63 (s, 4H); ^{13}C NMR (125 MHz, $CDCl_3$) δ 176.8, 135.8, 128.9, 128.6, 127.9, 42.4, 28.2; GC-MS (M^+) 189.

(3*R*,4*R*)-1-Benzyl-2,5-dioxopyrrolidine-3,4-diyl diacetate: Table 4, entry 7 [20]

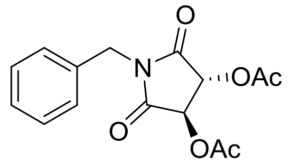

Reaction time: Step (a) at rt, 5 min and step (b), 8 h; R_f: 0.5 (1:2 EtOAc:Pet. ether); colorless thick oil, 145 mg, 85% yield; ^{1}H NMR (400 MHz, $CDCl_3$) δ 7.35-7.22 (m, 5H), 5.47 (s, 2H), 4.69 (d, J = 14.2 Hz, 1H), 4.63 (d, J = 14.2 Hz, 1H), 2.11 (s, 6H); ^{13}C NMR (100 MHz, $CDCl_3$) δ 169.8, 169.1, 134.5, 128.8, 128.7, 128.3, 72.7, 43.1, 20.3; GC-MS (M+) 305.

1-Benzylpiperidine-2,6-dione: Table 4, entry 8 [21]

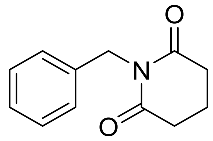

Reaction time: Step (a) at rt, 15 min and step (b), 4 h; R_f: 0.5 (1:2 EtOAc:Pet. ether); colorless oil, 111 mg, 98% yield; ^{1}H NMR (400 MHz, $CDCl_3$) δ 7.31-7.26 (m, 2H), 7.24-7.13 (m, 3H), 4.88 (s, 2H), 2.60 (t, J = 6.5 Hz, 4H), 1.87 (quintet, J = 6.6 Hz, 2H); ^{13}C NMR (100 MHz, $CDCl_3$) δ 172.4, 137.3, 128.8, 128.4, 127.4, 42.7, 32.9, 17.1; GC-MS (M^+) 203.

4. Mechanistic study:

A plausible reaction mechanism of our imide formation protocol has been depicted in Figure 1. We believe that DMSO scavenges the radical species **I** generated by a thermal homolytic cleavage of APS to form an intermediate **II**. The radical species **I** also reacts with the amic acid to generate carboxyl radical **III**. The two radical intermediates **II** and **III** form a peroxy compound **IV**, which cleaves to furnish the expected imide product **3**. The formation of the by-product methylsulfonylmethane (MSM) was confirmed by ^{1}H NMR (page no. S18) and GC (page no. S19). The formation of MSM was also observed in a blank reaction without the amic acid, however the rate of MSM formation was much faster than in the presence of amic acid. It suggests that DMSO is acting as a scavenger of radical species **I** and its involvement is necessary for the transformation. The radical mechanism was also confirmed by performing the standard reaction in the presence of TEMPO, which inhibits the generated radicals and expected transformation was not observed (page no. S20). An alternative radical chain mechanism cannot be ruled out.

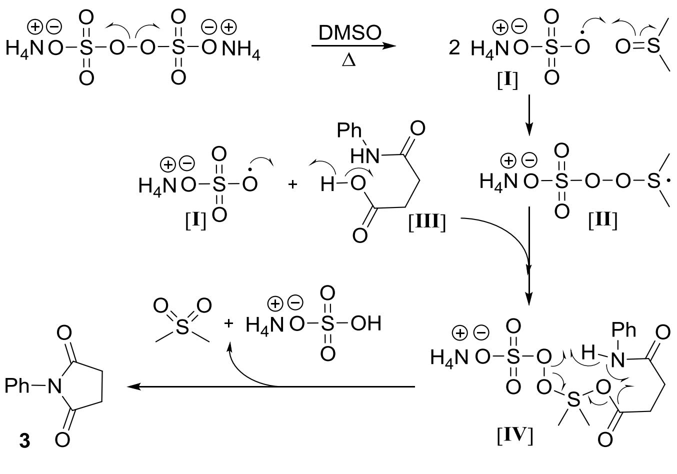

Figure 1: A plausible reaction mechanism.

^{1}H NMR analysis shows formation of MSM:

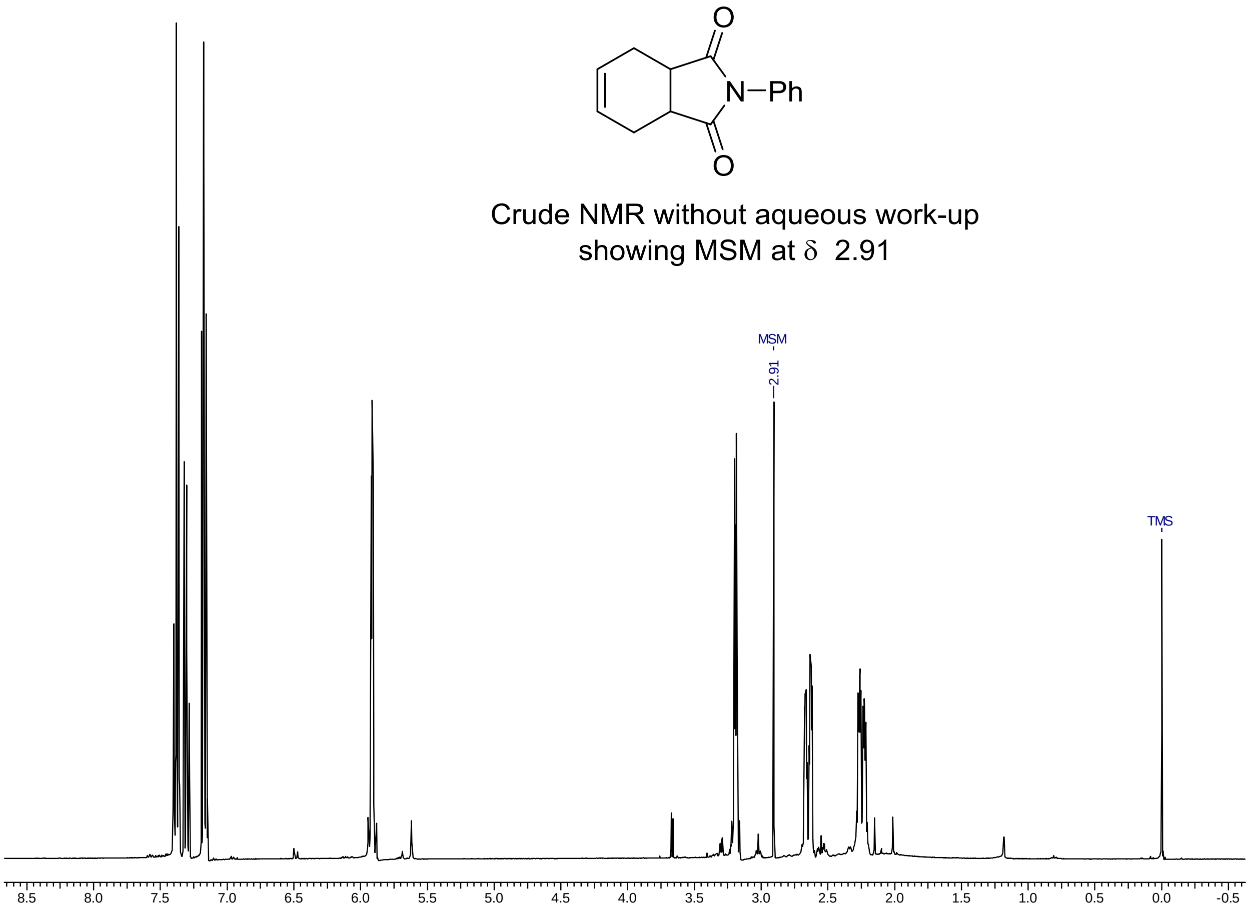

GC analysis:

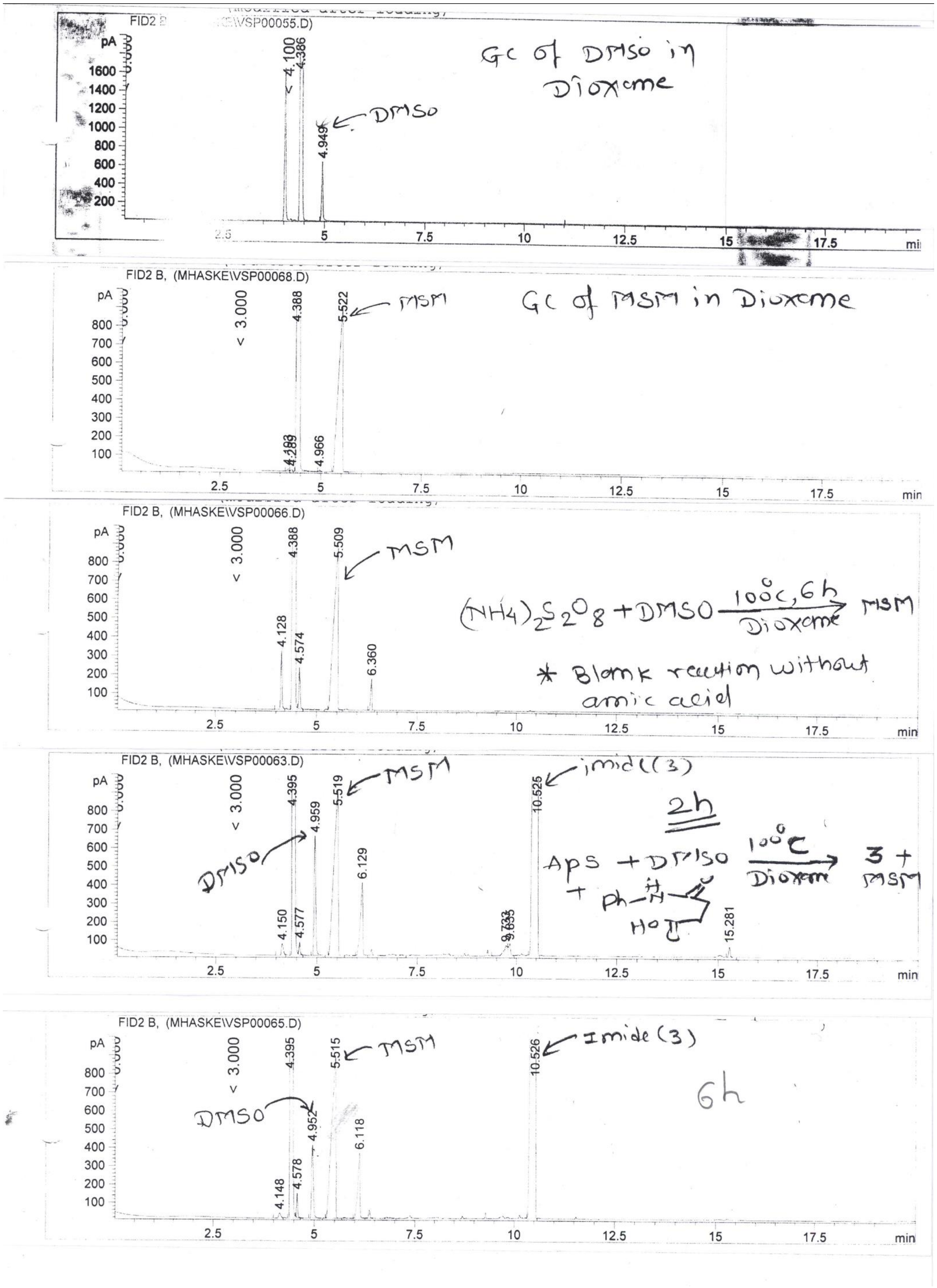
FID2 B, (MHASKE\VSP00055.D)
GC of DMSO in Dioxane
DMSO
FID2 B, (MHASKE\VSP00068.D)
MSM
GC of MSM in Dioxane
FID2 B, (MHASKE\VSP00066.D)
MSM
(NH4)2S2O8 + DMSO → MSM
100°C, 6h
Dioxane
* Blank reaction without amic acid
FID2 B, (MHASKE\VSP00063.D)
MSM
imide (3)
DMSO
2h
APS + DMSO → 3 + MSM
100°C
Dioxane
FID2 B, (MHASKE\VSP00065.D)
MSM
Imide (3)
DMSO
6h

Radical trapping experiment:

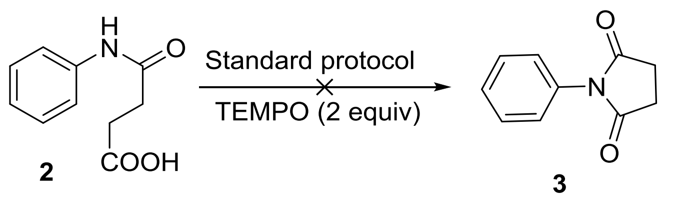

To a reaction mixture containing amic acid **2** (38 mg, 0.2 mmol), ammonium persulfate (91 mg, 0.4 mmol) and DMSO (30 µl, 0.4 mmol) in 1,4-dioxane (2 mL) was added TEMPO (63 mg, 0.4 mmol) and the resulting mixture was heated for 6 h at 100 °C.

Observation: After 6 h almost all amic acid **2** remains unreacted (TLC) and we did not observe formation of product **3**. TEMPO inhibits the reaction completely, hence justify that this is a radical transformation.

5. Experimental procedures for the synthesis of Vernakalant:

***tert*-Butyl 7-azabicyclo[4.1.0]heptane-7-carboxylate** ((±)-**7**) [22]

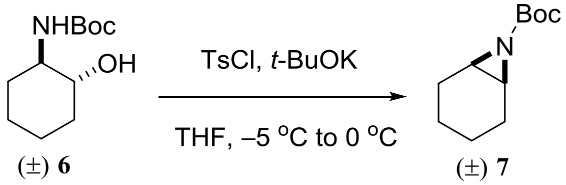

To a solution of *trans*-*tert*-butyl (2-hydroxycyclohexyl)carbamate ((±)-**6**, 1 g, 4.65 mmol) and tosyl chloride (1.33 g, 6.97 mmol) in THF (100 mL) was added potassium *tert*-butoxide (1.56 g, 13.94 mmol) portionwise at −5 °C. The resulting reaction mixture was allowed to stir at the same temparature for 15 min, then warmed to 0 °C and stirred until completion of the reaction (monitored by TLC, 90 min). The white slurry formed was filtered through celite and washed with ethyl acetate (2 × 50 mL). The filtrate was concentrated under vacuo and the residue was purified by column chromatography (neutral alumina, 1:19 EtOAc:Pet. ether) to afford pure aziridine (±)-**7** (796 mg, 87% yield) as a colorless thick oil. R_f: 0.5 (1:19 EtOAc:Pet. ether); ^{1}H NMR (500 MHz, $CDCl_3$) δ 2.51-2.45 (m, 2H), 1.88-1.81 (m, 2H), 1.75-1.67 (m, 2H), 1.38 (s, 9H), 1.36-1.30 (m, 2H), 1.20-1.11 (m, 2H);^{13}C NMR (125 MHz, $CDCl_3$) δ 163.3, 80.5, 36.9, 27.9, 23.7, 19.8; HRMS-ESI (m/z) calcd $(C_{12}H_{23}NNaO_3)^+$ $[M+(MeOH)+Na]^+$: 252.1576, found: 252.1576.

***trans-tert*-Butyl (2-(3,4-dimethoxyphenethoxy)cyclohexyl)carbamate** ((±)-**8**)

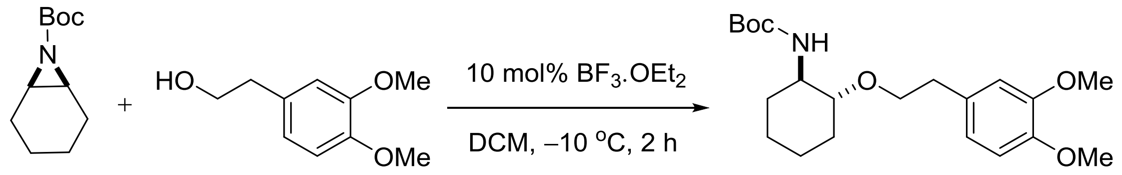

(±) **7** (±) **8**

To a solution of 2-(3,4-dimethoxyphenyl)ethan-1-ol (313 mg, 1.72 mmol) and $BF_3{\cdot}OEt_2$ (22 µL; 0.172 mmol) in anhydrous dichloromethane (0.5 mL) was slowly added a solution of aziridine (±)-**7** (340 mg; 1.72 mmol) in anhydrous dichloromethane (5 mL) over a period of 45 min at –10 °C. The resulting reaction mixture was stirred at the same temperature until completion of the reaction (monitored by TLC, 2 h). The reaction mixture was quenched by adding saturated aqueous $NaHCO_3$ (5 mL). The aqueous layer was extracted with dichloromethane (3 × 25 mL) and the combined organic layers were dried over sodium sulfate. Dichloromethane was filtered and concentrated under vacuo to obtain a residue, which was purified by column chromatography (silica gel, 1:3 EtOAc:Pet. ether) to furnish pure product (±)-**8** (490 mg, 75% yield) as a semi-solid: R_f: 0.4 (1:3 EtOAc:Pet. ether); ^{1}H NMR (400 MHz, $CDCl_3$) δ 6.77-6.67 (m, 3H), 4.48 (bs, 1H), 3.80 (s, 3H), 3.78 (s, 3H), 3.73-3.66 (m, 1H), 3.52-3.44 (m, 1H), 3.39-3.28 (m, 1H), 3.05-2.96 (m, 1H), 2.73 (t, J = 6.9 Hz, 2H), 2.11-2.01 (m, 1H), 1.94-1.86 (m, 1H), 1.67-1.57 (m, 2H),1.38 (s, 9H), 1.30-1.05 (m, 4H); ^{13}C NMR (100 MHz, $CDCl_3$) δ 155.8, 148.8, 147.5, 131.8, 120.8, 112.4, 111.3, 80.3, 79.0, 69.6, 55.9, 55.8, 53.7, 36.4, 31.1, 29.8, 28.4, 23.8, 23.6; HRMS-ESI (m/z) calcd $(C_{21}H_{33}NNaO_5)^+$ $[M+Na]^+$: 402.2256, found: 402.2256.

(3*R*)-1-(2-(3,4-Dimethoxyphenethoxy)cyclohexyl)-2,5-dioxopyrrolidin-3-yl acetate ((±) **10)**

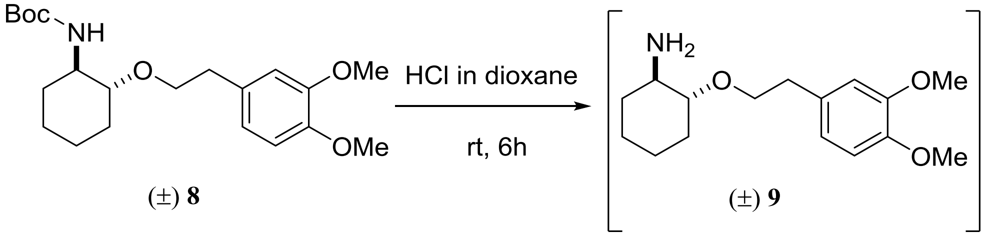

To a round bottom flask containing compound (±) **8** (450 mg, 1.19 mmol) in dioxane (5 mL), was added 4 M HCl in dioxane (12 mL) and the reaction mixture was stirred at room temperature untilall starting material converts to the corresponding primary amine (±)-**9** (monitored by TLC, 6 h). Dioxane and other volatiles were evoporated on rotavapour to obtain hydrochloride salt of amine (±)-**9**, which was dissolved in 4 M aqueous NaOH (20 mL) and diethyl ether (20 mL). The resulting biphasic mixture was stirred at room temperature for 30 min. The aqueous layer was extracted with diethyl ether (3 × 25 mL). The combined organic layers were dried over sodium sulfate and concentrated under vacuo to provide amine (±)-**9** as a thick oil, which was used for next step without further purification.

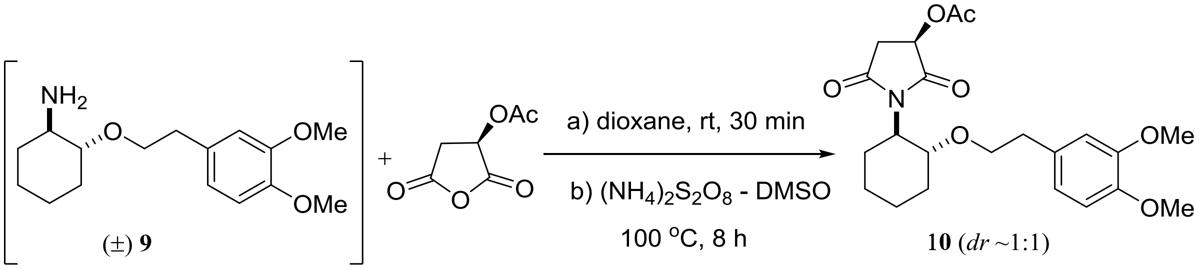

A solution of the crude amine (±)-**9** from above step and 2(*R)*-acetoxysuccinic anhydride (206 mg, 1.30 mmol) in dioxane (10 mL) were stirred at room temperature for 30 min Ammonium persulfate (539mg, 2.36mmol) and DMSO (168 µL, 2.36mmol) were added to the reaction mixture and it was heated at 100 °C for 8 h. After completion, the reaction mixture was filtered

through a cotton plug and dioxane was evaporated under vacuo to afford a crude product. It was dissolved in ethyl acetate and washed with dilute HCl, saturated aqueous $NaHCO_3$ and brine. Organic layer was dried over anhydrous Na_2SO_4 and the solvent was evaporated under vacuo to provide imide **10**, which was further purified by column chromatography (silica gel, 1:1 EtOAc:Pet. ether) to get a pure imide **10** (dr ~1:1 by NMR, 393mg, 79% yield) as thick oil: R_f: 0.6 (1:1 EtOAc:Pet. ether); ^{1}H NMR (500 MHz, $CDCl_3$) δ 6.74 (t, J = 8.5 Hz, 1H), 6.65-6.60 (m, 2H), 5.00-4.88 (m, 0.5H), 4.75-4.60 (m, 0.5H), 3.91-3.83 (m, 2H), 3.80 (s, 3H), 3.79 (s, 3H), 3.78-3.72 (m, 1H), 3.38-3.24 (m, 1H), 2.75-2.56 (m, 3H), 2.35-2.25 (m, 1H), 2.19-2.11 (m, 1H), 2.04 (s, 3H), 2.01-1.88 (m, 1H), 1.75-1.55 (m, 3H), 1.29-1.07 (m, 3H); ^{13}C NMR (125 MHz, $CDCl_3$) δ 173.7, 173.5, 173.3, 169.69, 169.66, 148.6, 148.4, 147.3, 147.2, 132.3, 132.1, 120.7, 120.4, 112.6, 112.1, 111.03, 110.97, 75.7, 75.1, 68.9, 68.8, 67.0, 66.8, 56.1, 55.86, 55.80, 55.77, 35.9, 35.3, 35.2, 31.04, 30.97, 28.1, 27.8, 25.0, 23.9, 20.5; HRMS-ESI (m/z) calcd $(C_{22}H_{29}NNaO_7)^+$ $[M+Na]^+$: 442.1842, found: 442.1837.

(3*R*)-1-(2-(3,4-Dimethoxyphenethoxy)cyclohexyl)pyrrolidin-3-ol [Venakalant (11)]: [23]

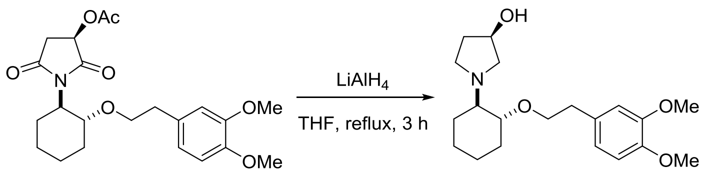

10 **venakalant 11** (*dr* ~1:1)

To a solution of imide **10** (50 mg, 0.119 mmol) in THF (2 ml) was added $LiAlH_4$ (27 mg, 0.715 mmol) at room temperature. The resulting suspension was heated to reflux for 3 h for completion of the reaction. The reaction mixture was then cooled to 0 °C and quenched by 6 M aqueous NaOH, followed by filteration through a celite pad with diethyl ether as a eluent. Water was

added to the filtrate and the aqueous layer was extracted with diethyl ether.The combined organic layers were washed with dilute HCl (2 × 25mL) to form water soluble hydrochloide salt of vernakalant **11.** The organic layer was discarded and the aqueous layer was basified by 6M aqueous NaOH to 10–12 pH. The basic aqueous phase was extracted with diethyl ether (3 x 25 mL). The combined organic layers were washed with brine, dried over Na_2SO_4 and concentrated under vacuo to afford crude product, which was purified by column chromatography (basic alumina, 2% MeOH in DCM) to furnish pure vernakalant **11** as a free base (*dr* ~1:1 by NMR, 40mg, 98% yield, thick oil): R_f: 0.2 (2% MeOH in DCM); ^1H NMR (400 MHz, CD_3OD) δ 6.88-6.83 (m, 2H), 6.78 (dd, *J* = 8.3, 1.7 Hz, 1H), 4.25-4.18 (m, 1H), 3.81 (s, 3H), 3.79 (s, 3H), 3.78-3.74 (m, 1H), 3.64-3.55 (m, 1H), 3.30-3.25 (m, 1H), 2.95 (dd, *J* = 10.3, 6.1 Hz, 0.5 H), 2.85 (dd, *J*= 10.3, 6.1 Hz, 0.5 H), 2.79 (t, *J* = 6.6 Hz, 2H), 2.77-2.73 (m, 1H), 2.65-2.54 (m, 2H), 2.36-2.28 (m, 1H), 2.10-1.85 (m, 3H), 1.71-1.55 (m, 3H), 1.35-1.18 (m, 4H); ^{13}C NMR (100 MHz, CD_3OD) δ 150.2, 148.9, 133.7, 122.3, 114.15, 114.12, 113.1, 81.1, 81.0, 71.21, 71.19, 70.7, 65.9, 65.7, 61.1, 60.2, 56.5, 56.4, 50.8, 50.2, 37.29, 37.26, 34.8, 30.3, 28.6, 28.3, 24.7, 24.2; HRMS-ESI (m/z) calcd $(C_{20}H_{32}NO_4)^+$ $[M+H]^+$: 350.2331, found: 350.2326.

6. References:

[1] Mhaske, S. B.; Argade, N. P. *J. Org. Chem.* **2001**, *66*, 9038.

[2] Foo, K.; Sella, E.; Thomé, I.; Eastgate, M. D.;. Baran, P. S. *J. Am. Chem. Soc.* **2014**, *136*, 5279.

[3] Akula, M. R.; Kabalka, G. W. *Synth. Commun.* **1998**, *28*, 2063.

[4] Kantam, M. L.; Prakash, B. V.; Reddy, C. V. *J. Mol. Catal. A- Chem.* **2005**, *241*, 162.

[5] Flitsch, W.; Rußkamp, P. *Heterocycles* **1984**, *22*, 541.

[6] Patil, S. V.; Mahale, K. A.; Gosavi, K. S.; Deshmukh, G. B.; Patil, N. S. *Org. Prep. Proced. Int.* **2013**, *45*, 314.

[7] Berthaa, F.; Gianga, L. T.; Fetter, J.; Kajtár-Peredyb, M.; Lempert, K.; Czirad, G. *J. Chem. Res.* **2003**, 759.

[8] Takayama, C.; Fujinami, A. *Pestic. Biochem. Phys.* **1979**, *12*, 163.

[9] Tan, A.; Koc, B.; Sahin, E.; Kishali, N. H.; Kara, Y. *Synthesis* **2011**, 1079.

[10] Guo, S.; Zhang, H.; Huang, L.; Guo, Z.; Xiong, G.; Zhao, J. *Chem. Commun.* **2013**, *49*, 8689.

[11] Davie, B. J.; Valant, C.; White, J. M.; Sexton, P. M.; Capuano, B.; Christopoulos, A.; Scammells, P. J. *J. Med. Chem.* **2014**, *57*, 5405.

[12] Huang, L.-F.; Tsai, L.-D.; Wu, W.-H. Flexible maleimide polymer and method for preparing the same, U.S. patent 2013/0172518 A1, Jul. 4, 2013.

[13] Gaina, C.; Ursache, O.; Gaina, V.; Buruiana, E.; Ionita, D. *Express Polym. Lett.* **2012**, *6*, 129.

[14] Sortino, M.; Postigo, A.; Zacchino, S. *Molecules* **2013**, *18*, 5669.

[15] Allen, L. J.; Cabrera, P. J.; Lee, M.; Sanford, M. S. *J. Am. Chem. Soc.* **2014**, *136*, 5607.

[16] Kalgutkar , A. S.; Crews , B. C.; Marnett, L. J. *J. Med. Chem*. **1996**, *39*, 1692.

[17] Maruyama, J.; Hidekazu, S. Reversible heat-sensitive recording material, U.S. patent 6,184,180 B1, Feb 6, 2001.

[18] Schreiber, K. C.; Fernandez, V. P. *J. Org. Chem*. **1961**, *26*, 1744.

[19] Kim, J.; Hong, S. H. *Org. lett*. **2014**, *16*, 4404.

[20] Vieira, A. S.; Ferreira, F. P.; Fiorante, P. F.; Guadagnin, R. C.; Stefani, H. A. *Tetrahedron* **2008**, *64*, 3306.

[21] Zhang, J.; Senthilkumar, M.; Ghosh, S. C.; Hong S. H. *Angew. Chem., Int. Ed*. **2010**, *49*, 6391.

[22] Mordini, A.; Russo, F.; Valacchi, M.; Zani, L.; Degl'Innocenti A.; Reginato, G. *Tetrahedron* **2002**, *58*, 7153.

[23] Limanto, J.; Ashley, E. R.; Yin, J.; Beutner, G. L.; Grau, B. T.; Kassim, A. M.; Kim, M. M.; Klapars, A.; Liu, Z.; Strotman, H. R.; Truppo, M. D. *Org. Lett*. **2014**, *16*, 2716.

7. Copies of ^1H, ^{13}C, DEPT Spectra

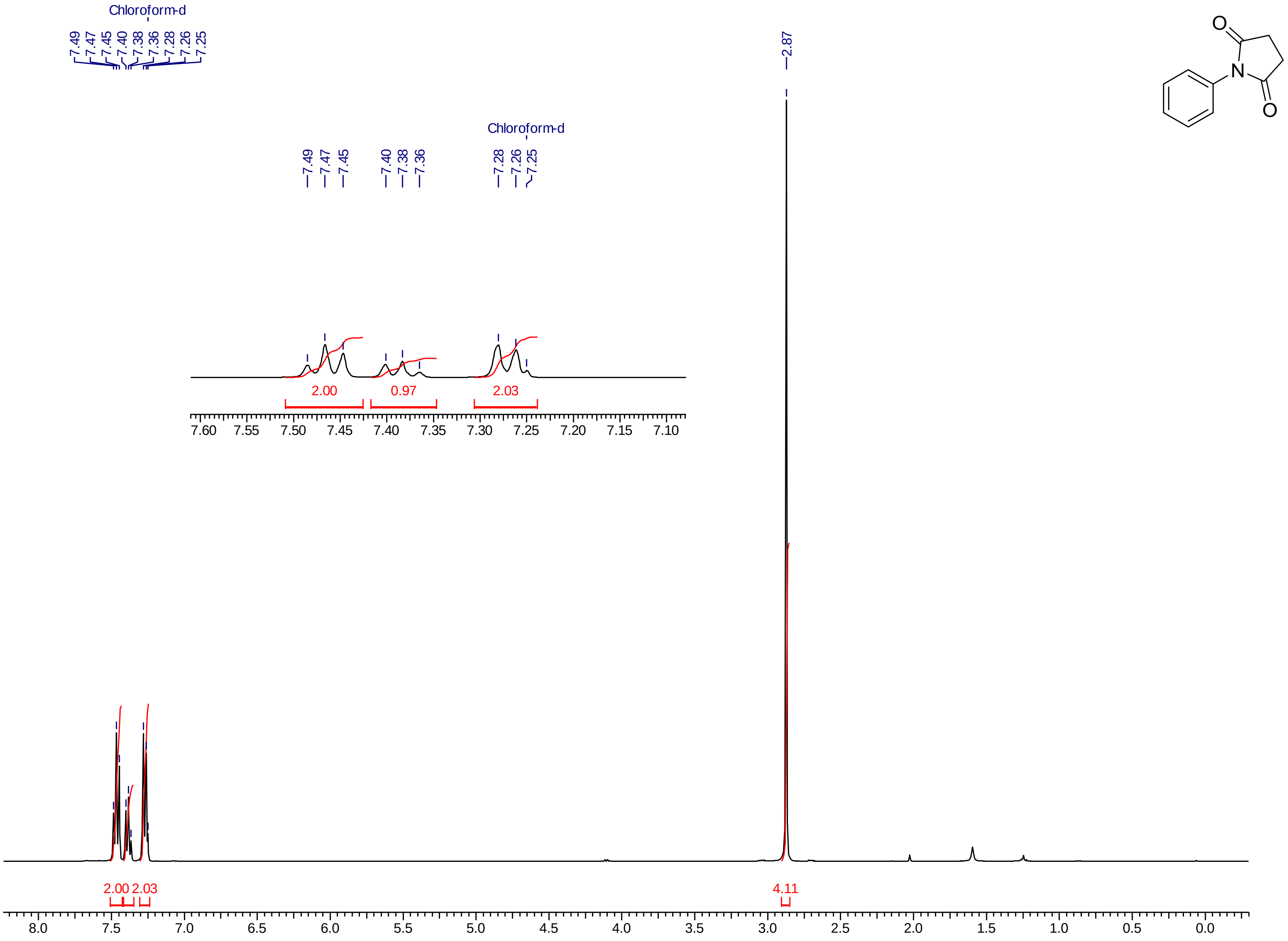

^{13}C NMR, 100 MHz

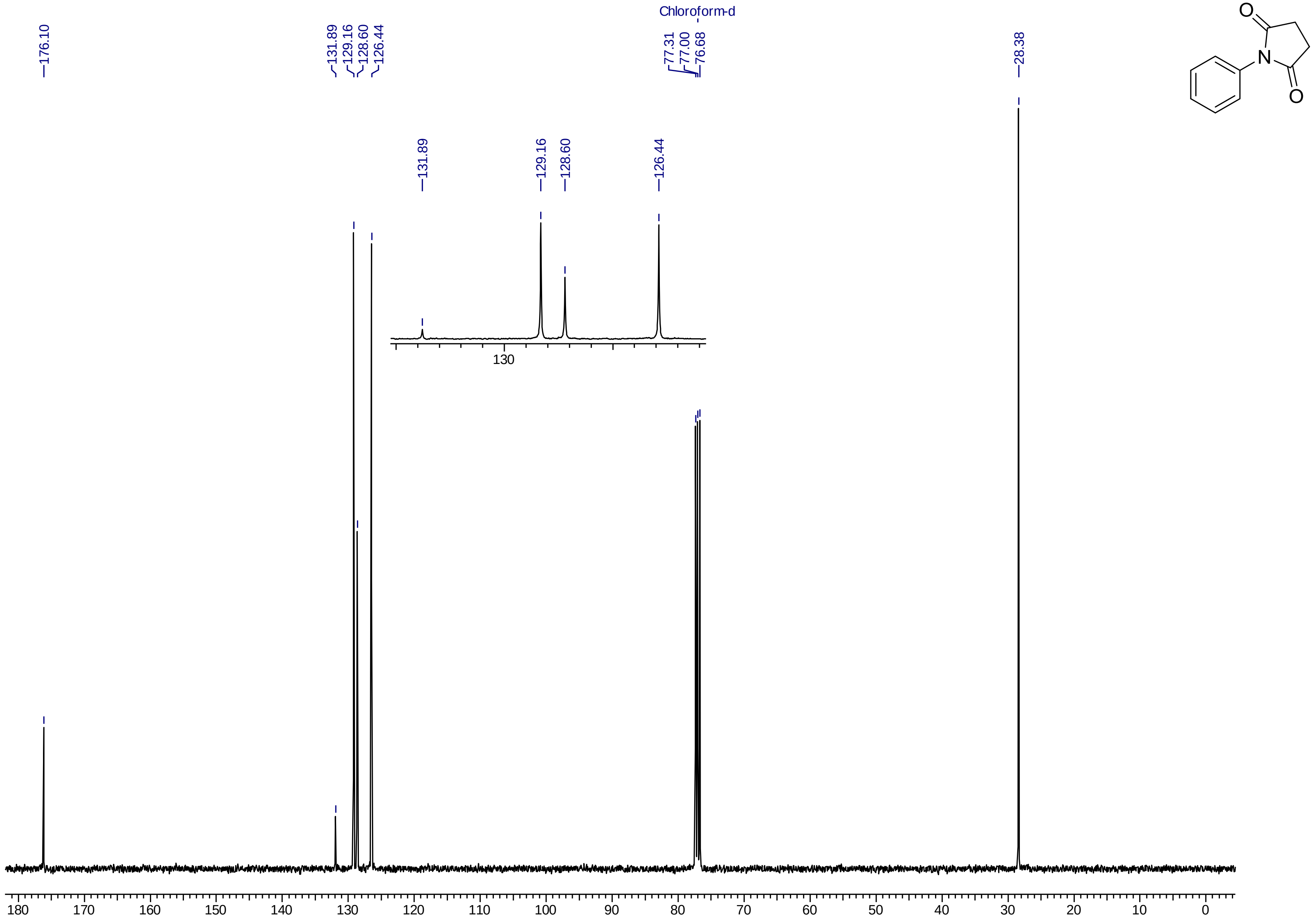

^{13}C NMR, DEPT, 100 MHz

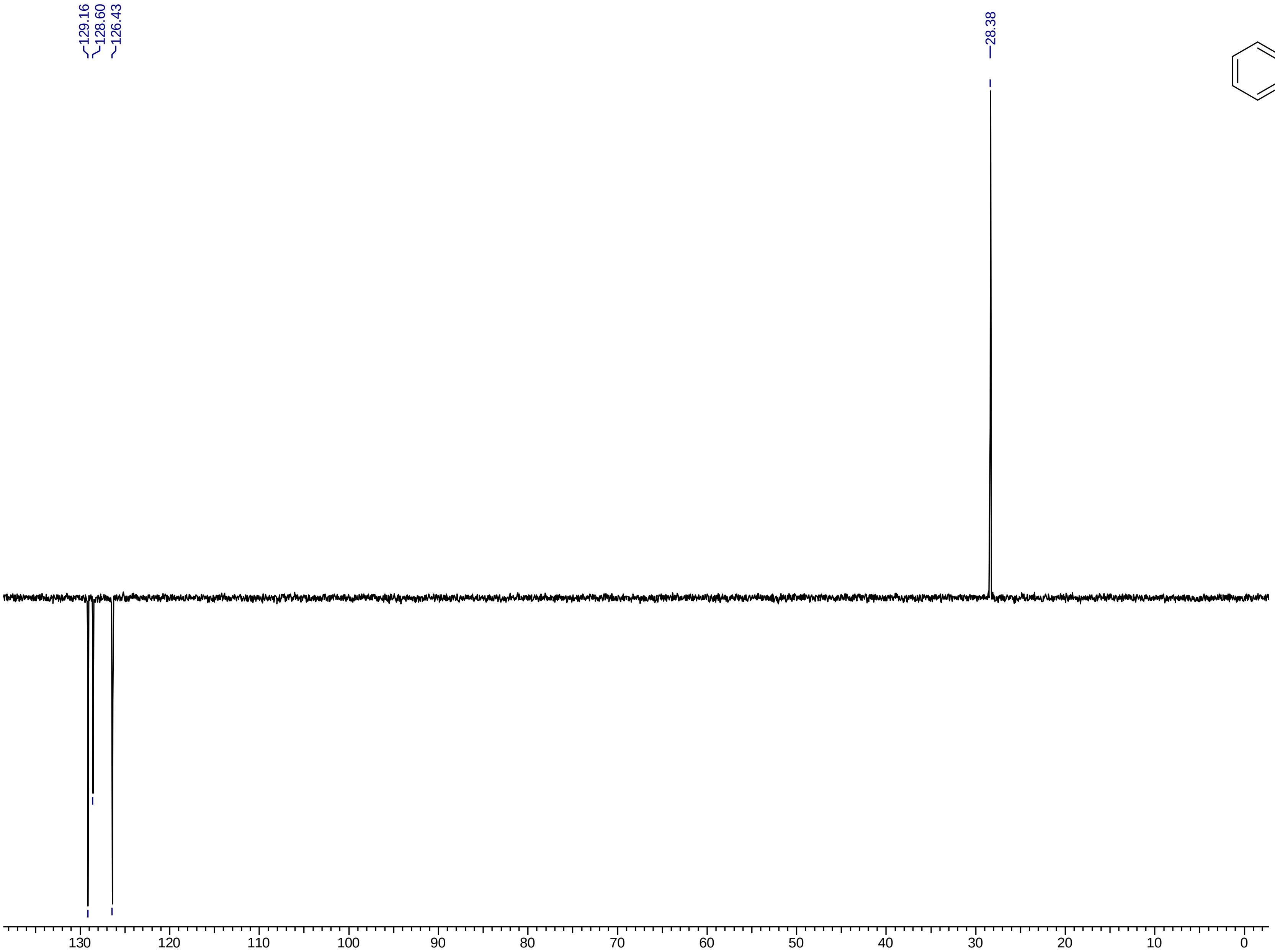

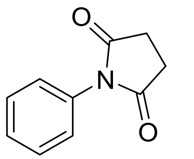

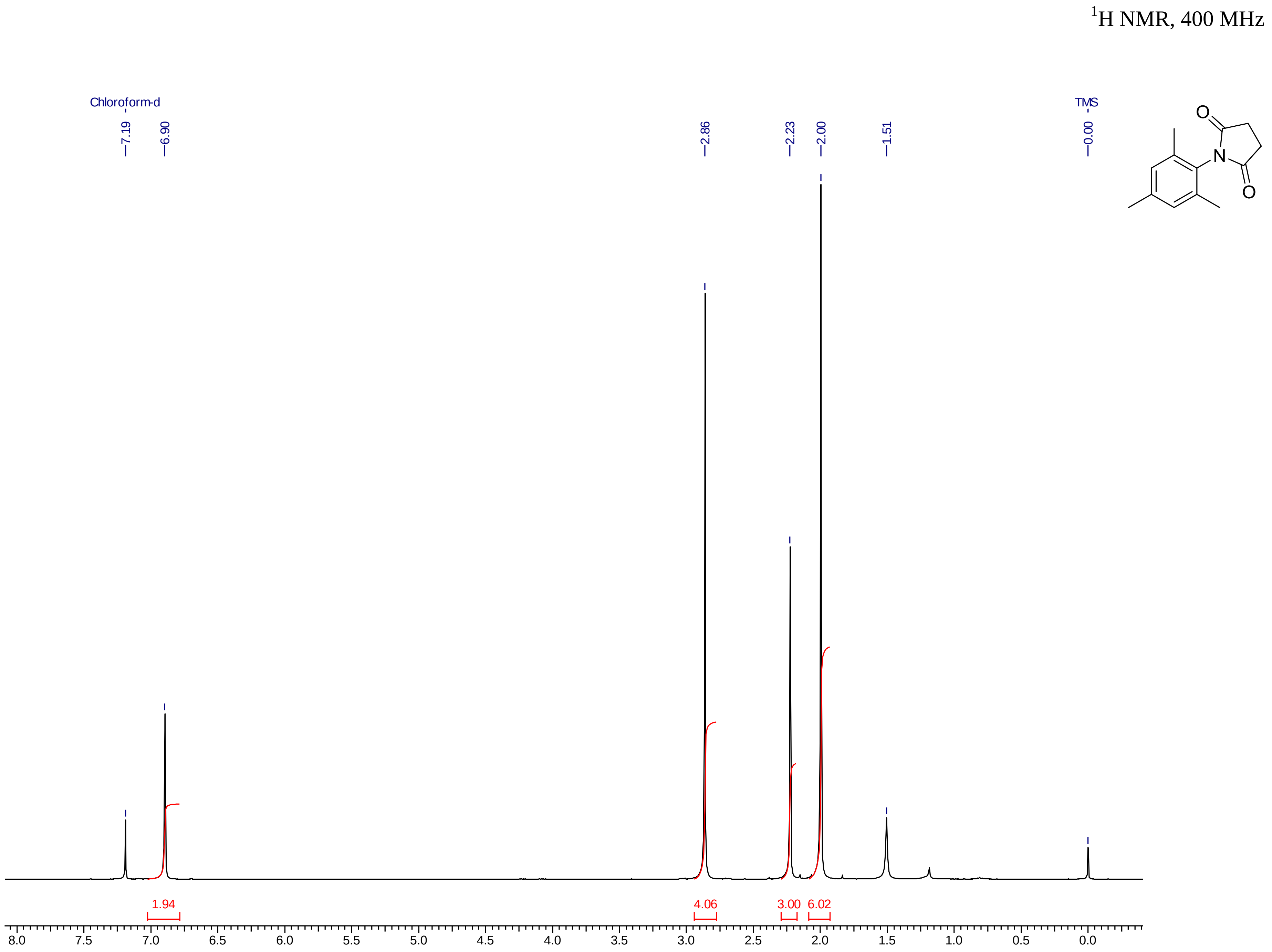

¹H NMR, 400 MHz
Chloroform-d
7.19
6.90
2.86
2.23
2.00
1.51
TMS
0.00
O
N
O
1.94
4.06
3.00
6.02
8.0
7.5
7.0
6.5
6.0
5.5
5.0
4.5
4.0
3.5
3.0
2.5
2.0
1.5
1.0
0.5
0.0

^{13}C NMR, 100 MHz

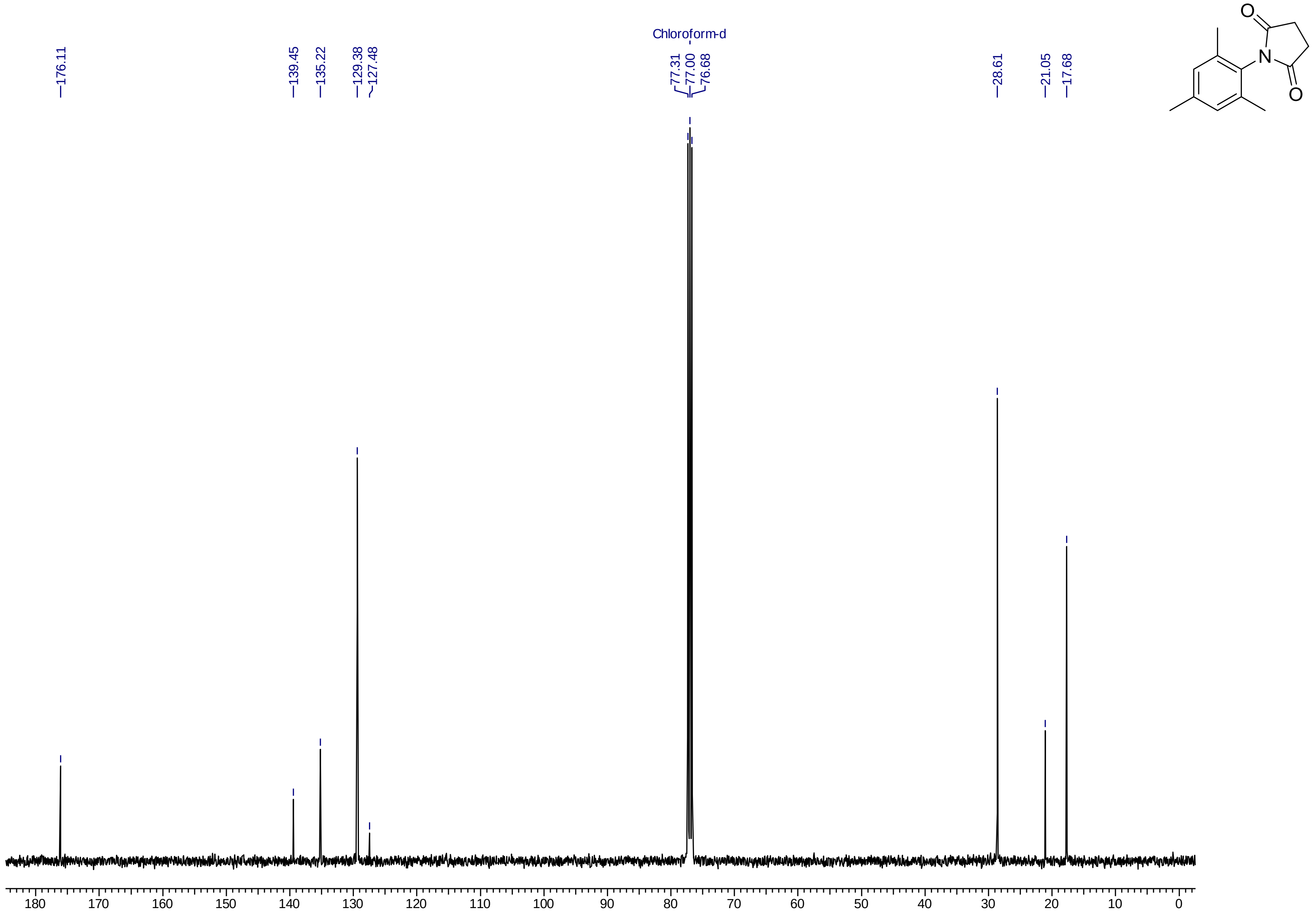

^{13}C NMR, DEPT, 100 MHz

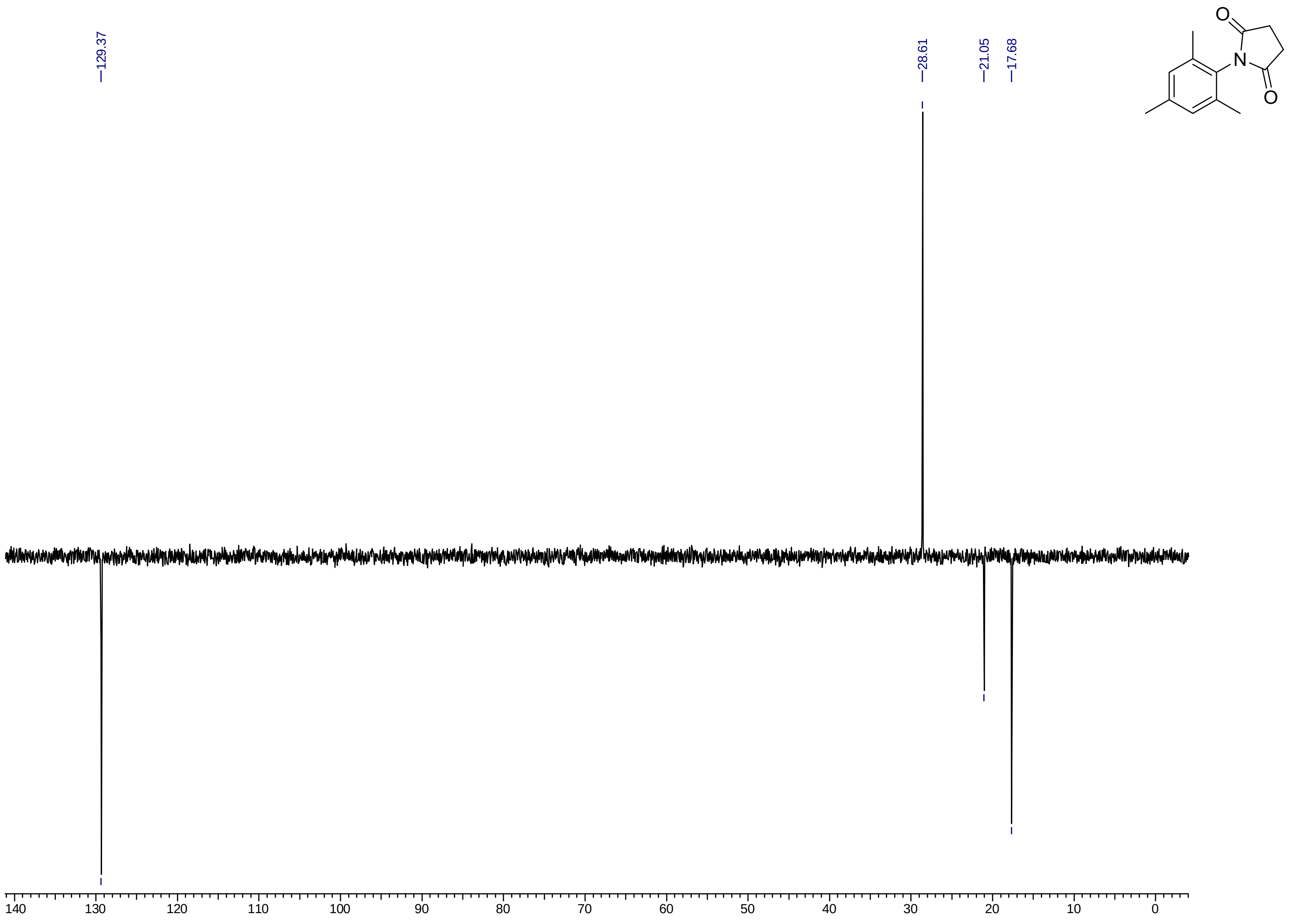

^{1}H NMR, 400 MHz

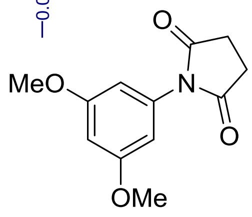

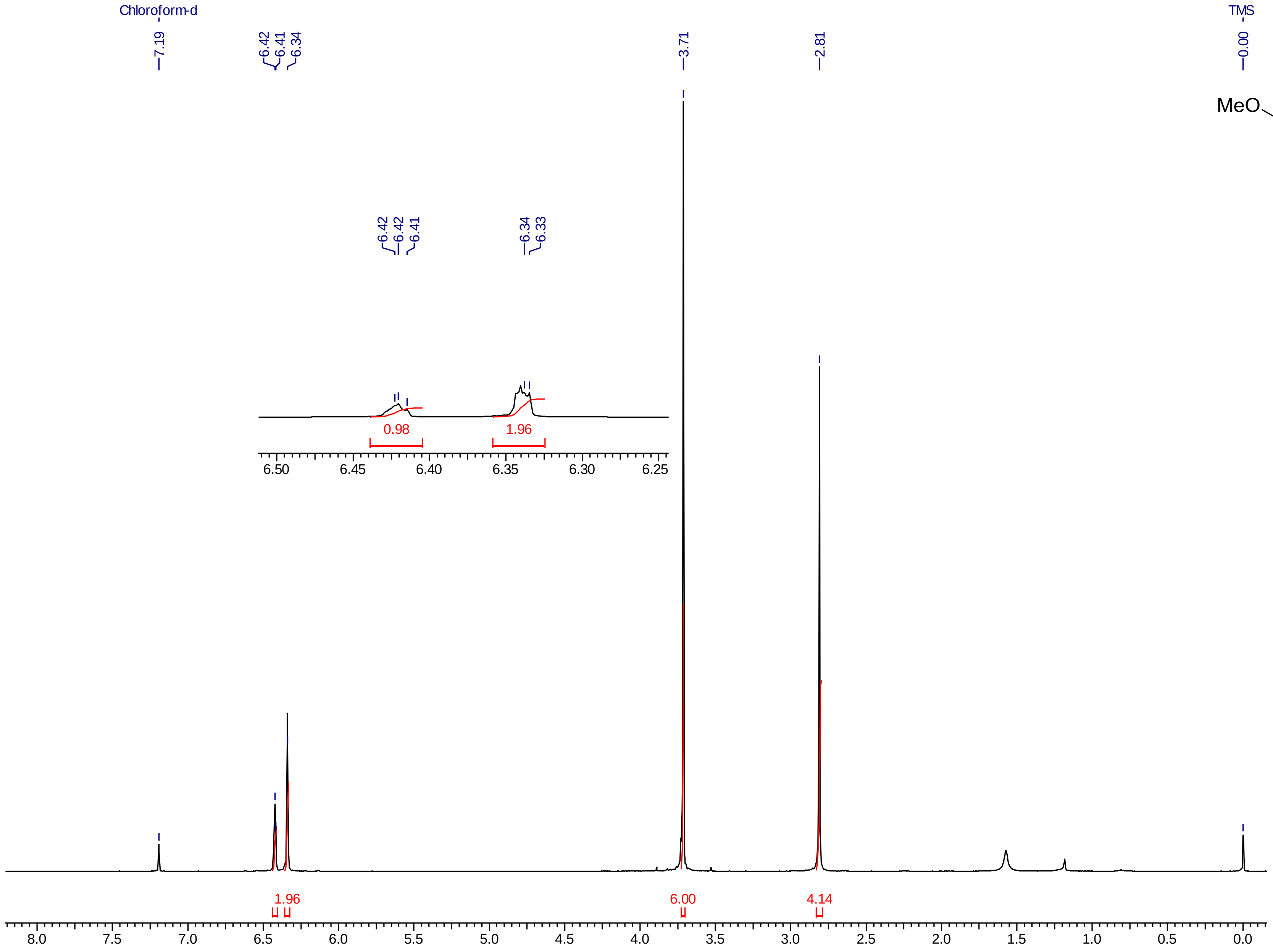

^{13}C NMR, 100 MHz

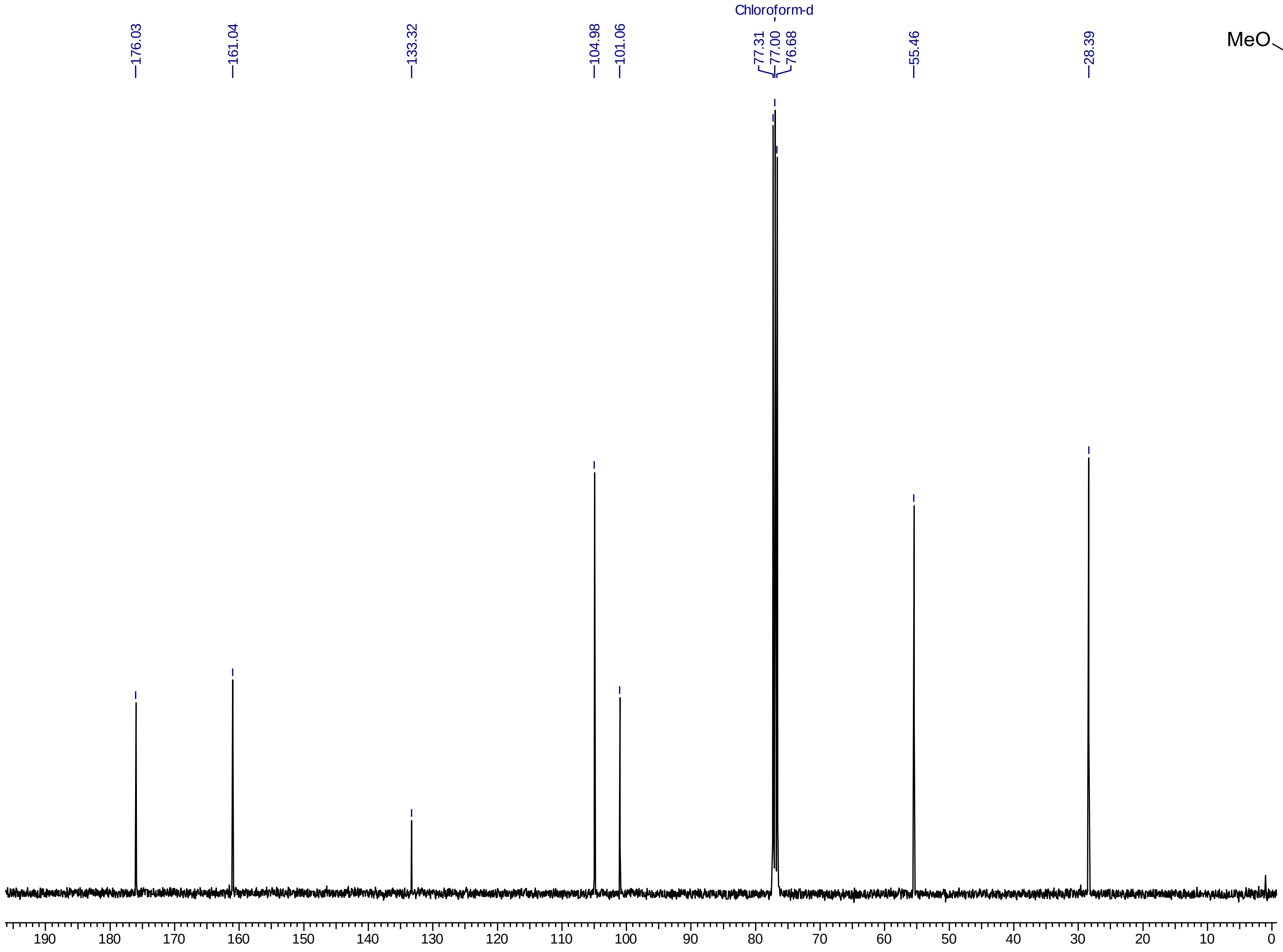

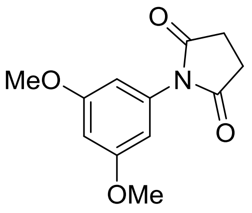

^{13}C NMR, DEPT, 100 MHz

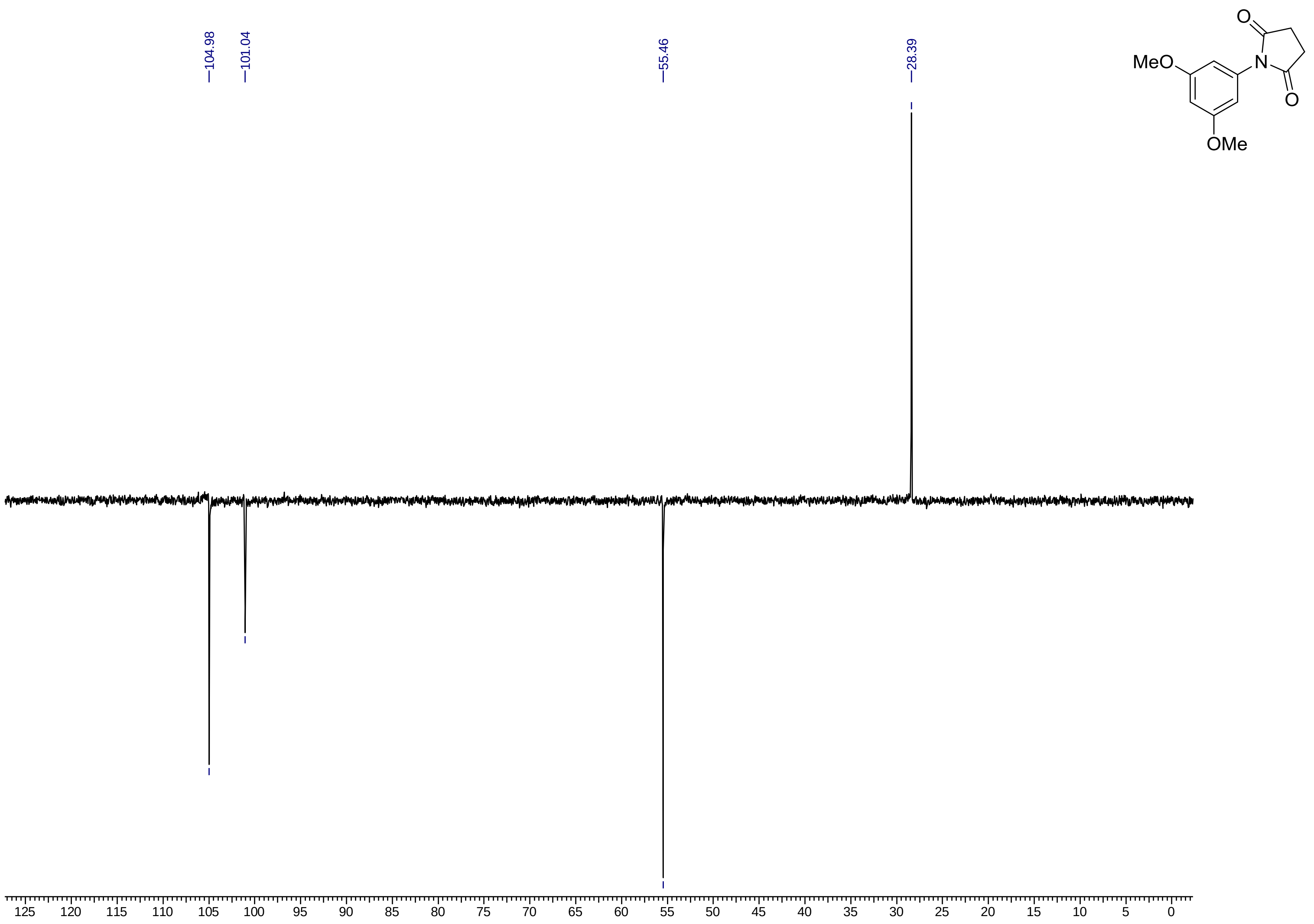

^{1}H NMR, 400 MHz

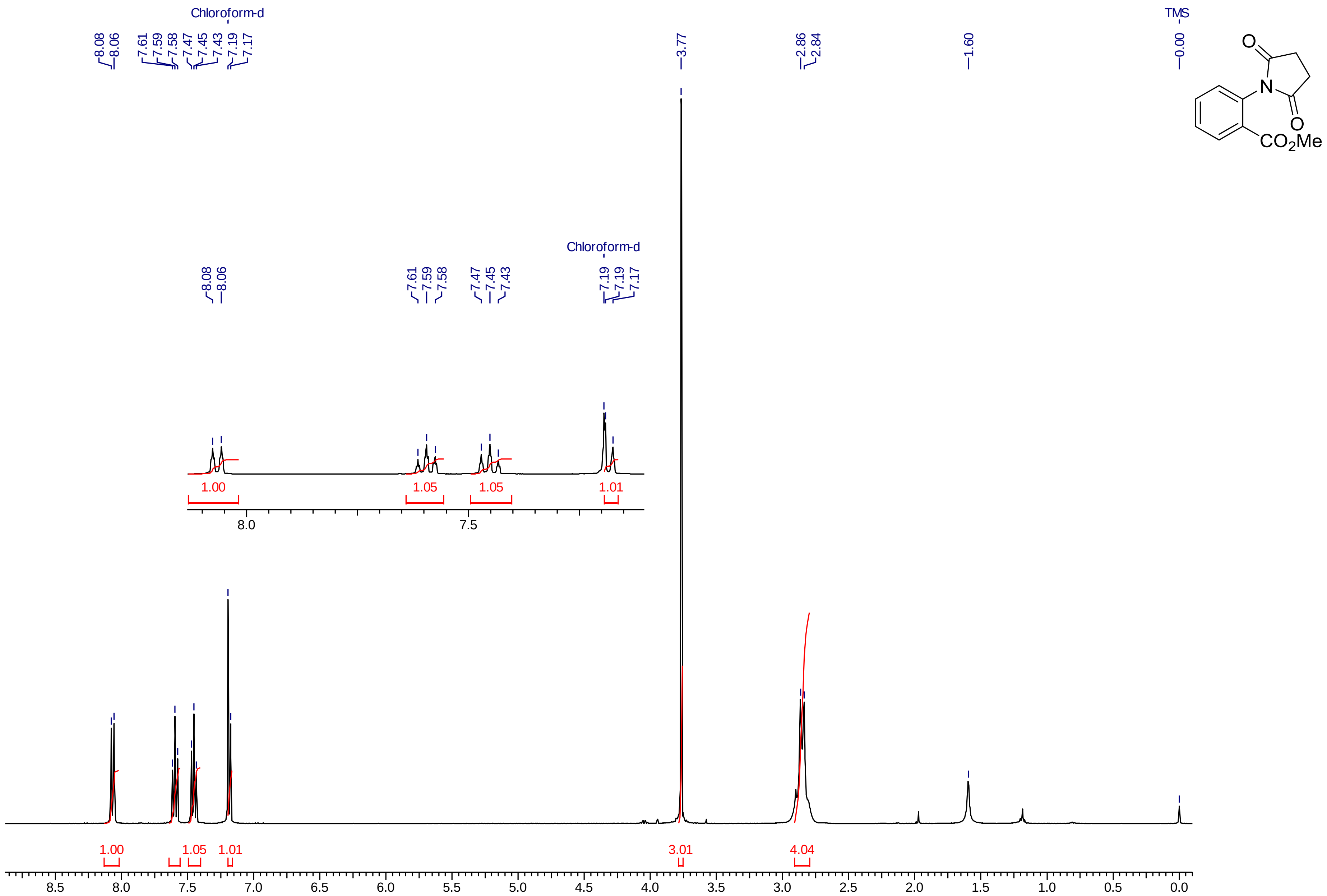

^{13}C NMR, 100 MHz

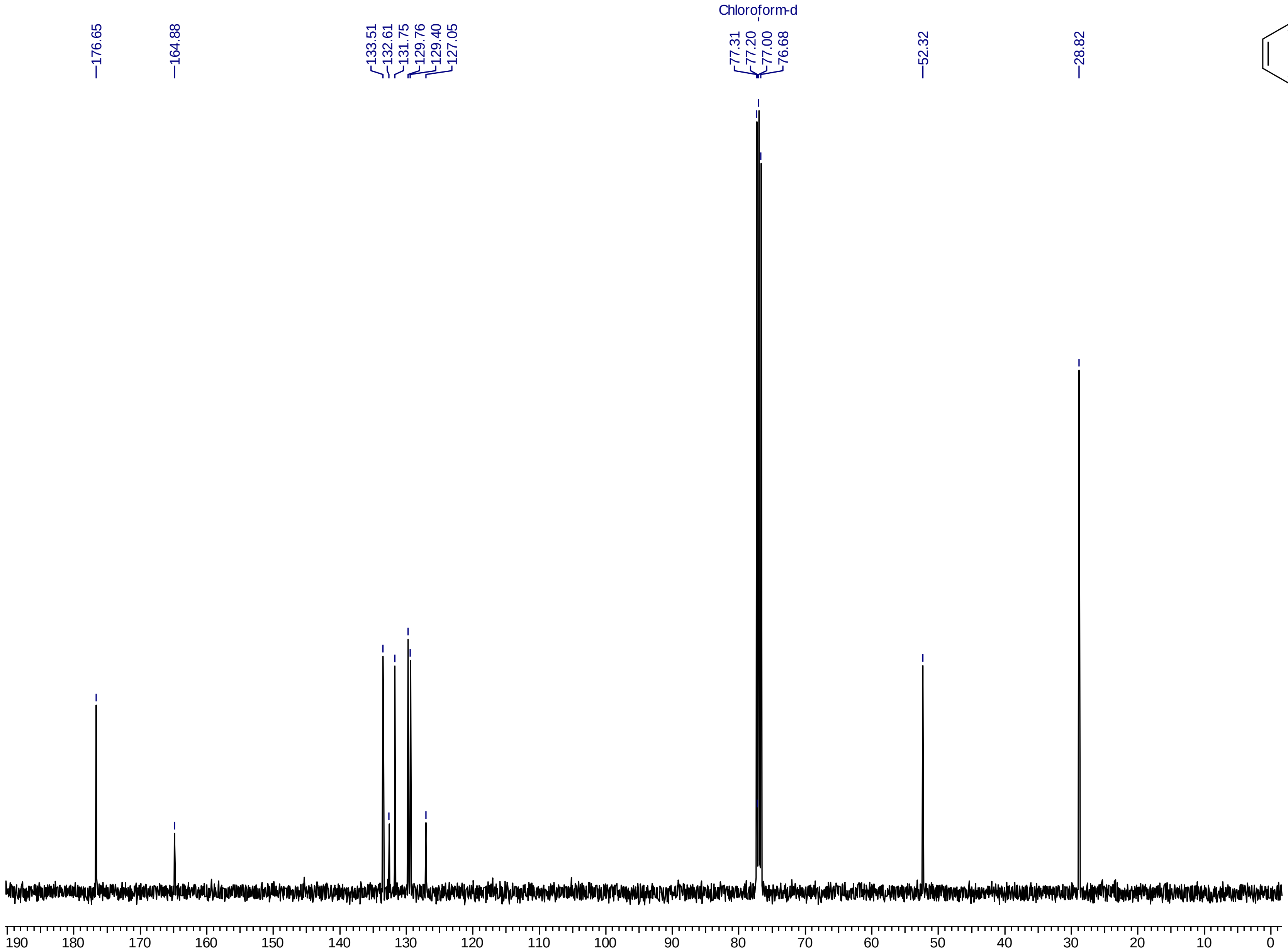

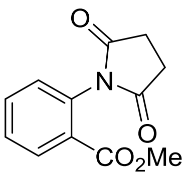

^{13}C NMR, DEPT, 100 MHz

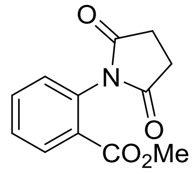

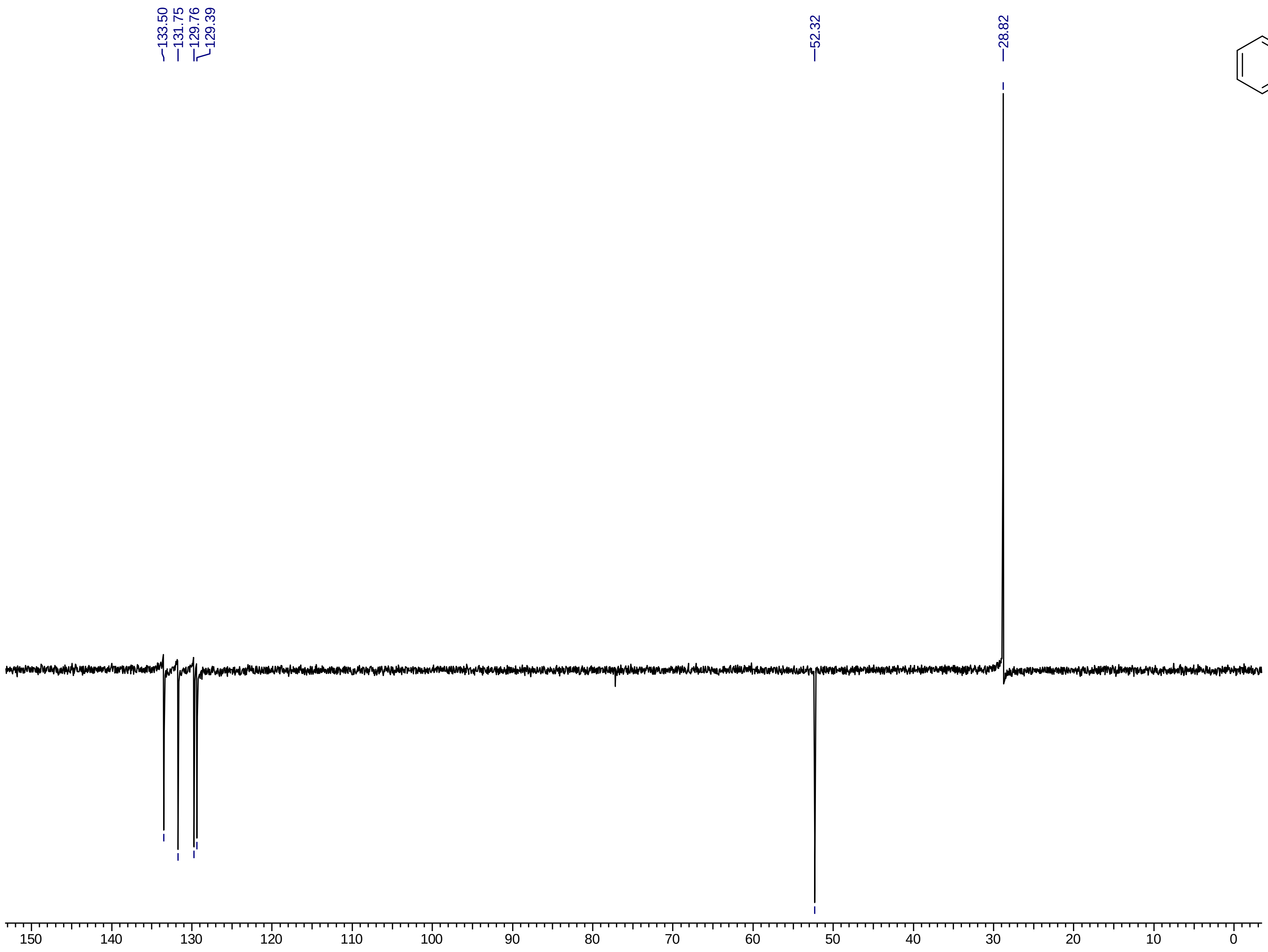

^{1}H NMR, 500 MHz

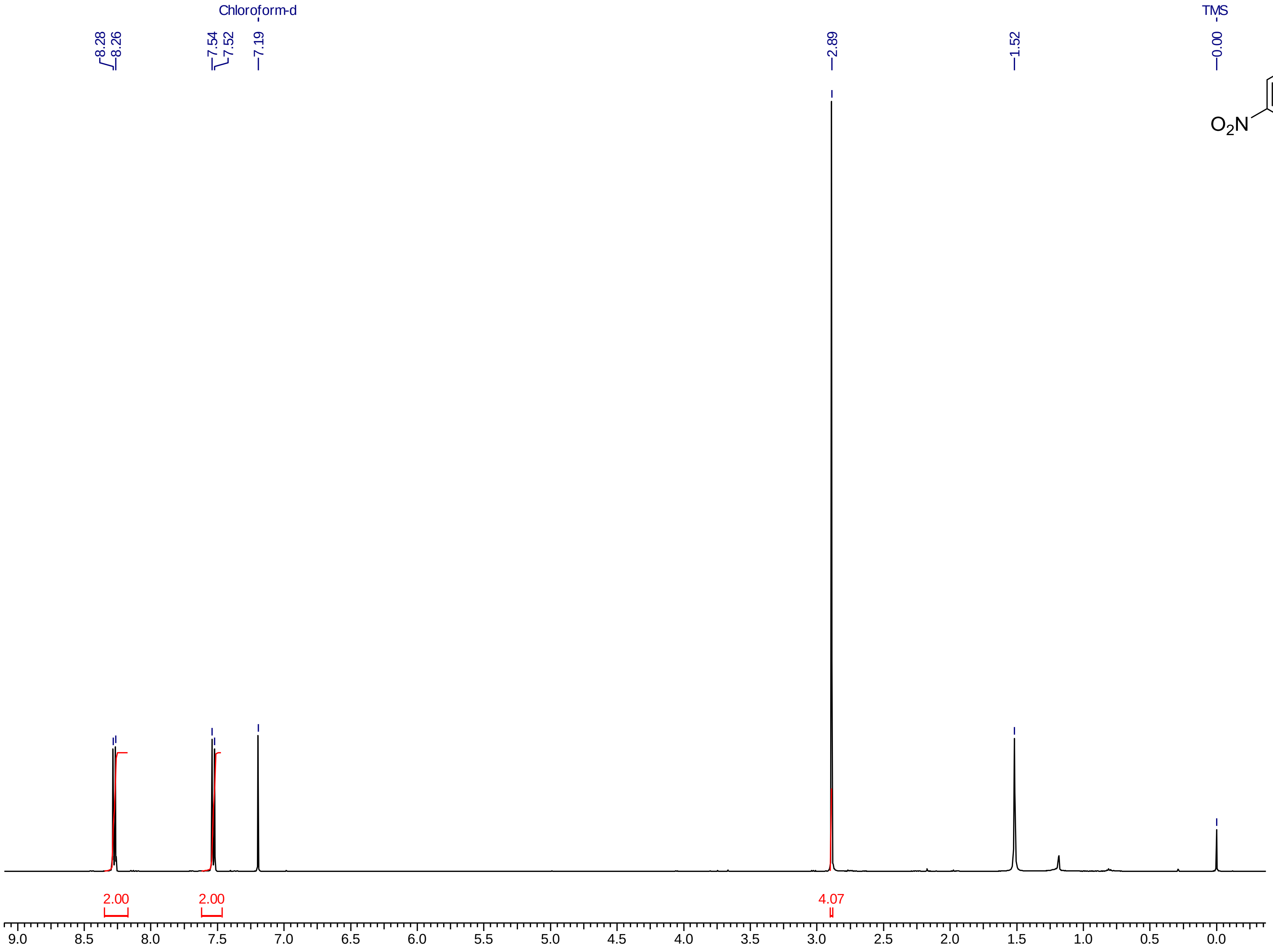

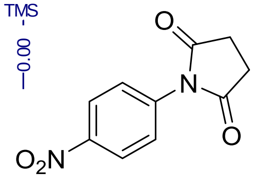

^{13}C NMR, 100 MHz

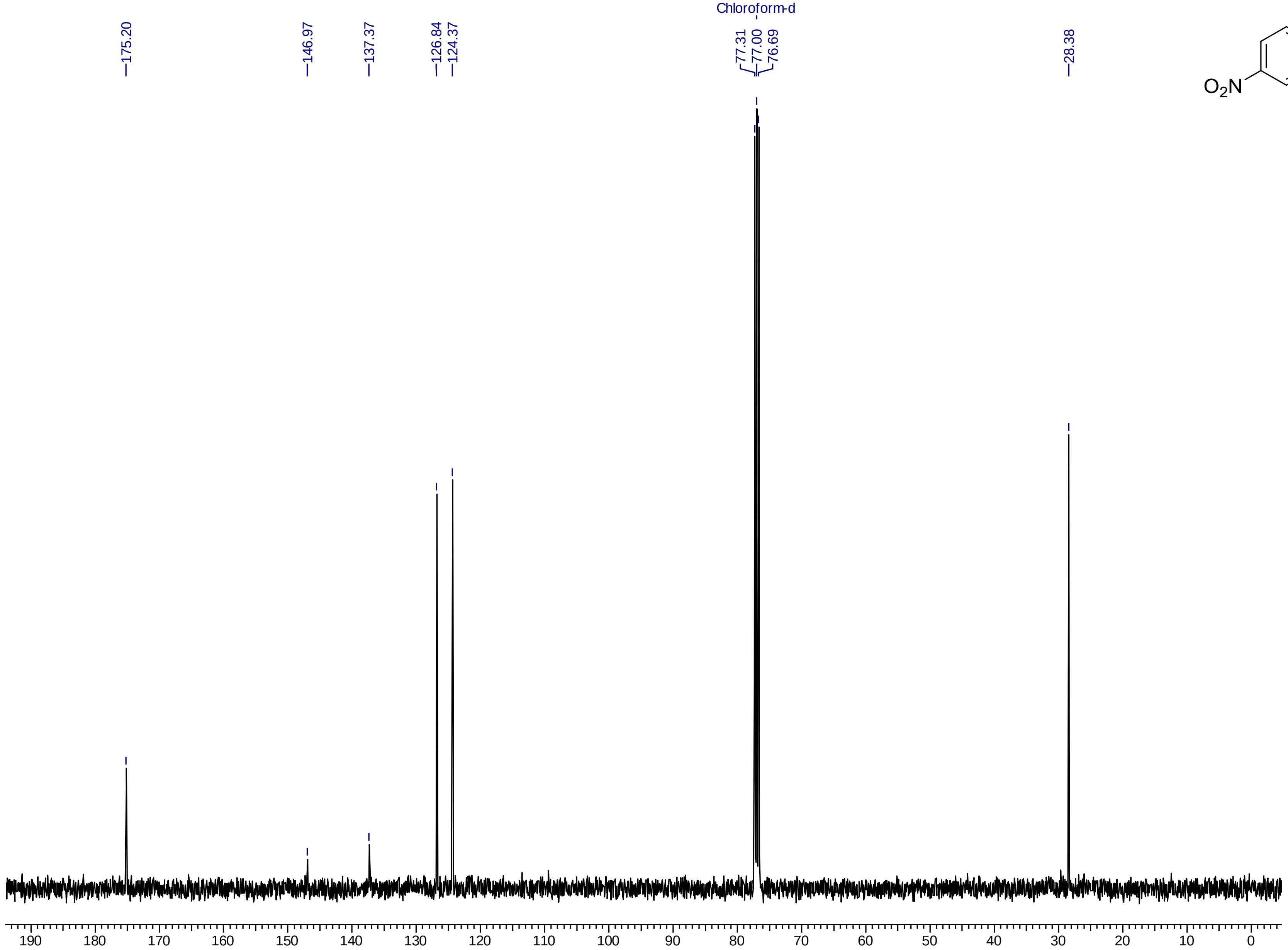

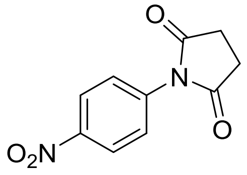

^{13}C NMR, DEPT, 100 MHz

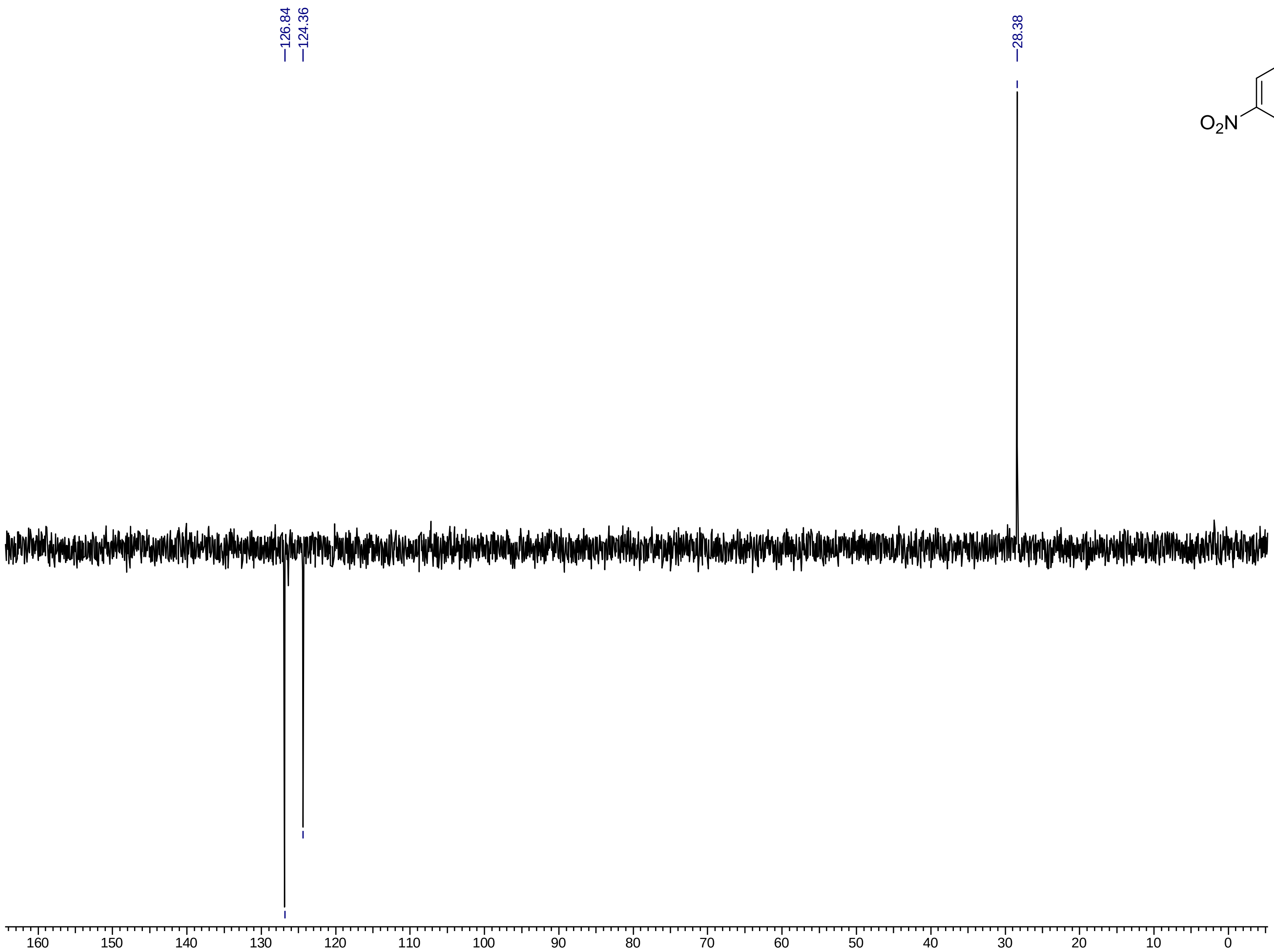

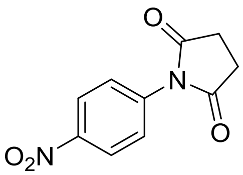

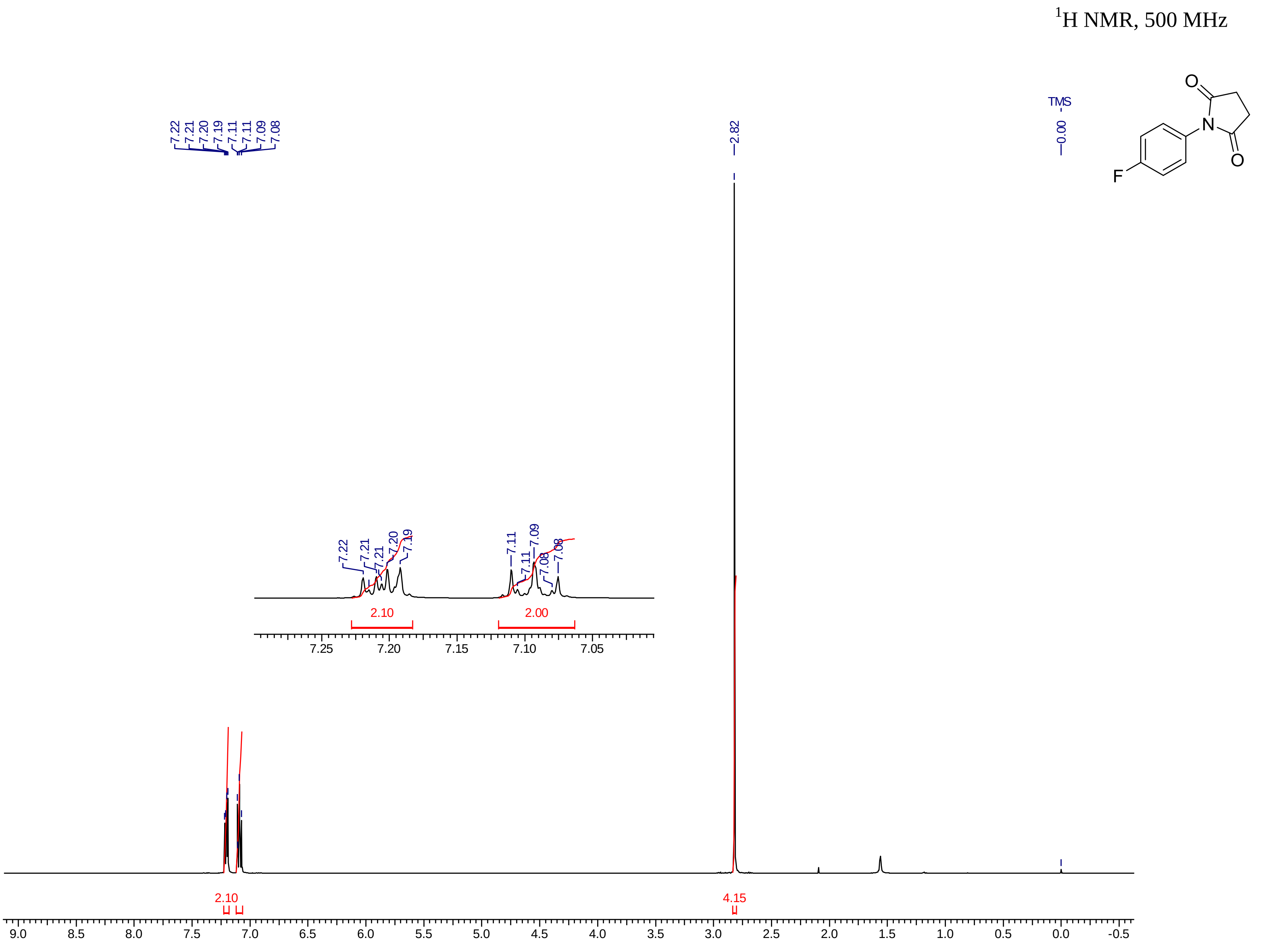

^{1}H NMR, 500 MHz
7.22 7.21 7.20 7.19 7.11 7.11 7.09 7.08
2.82
TMS
0.00
O
N
O
F
7.22 7.21 7.21 7.20 7.19
7.11 7.11 7.09 7.08 7.08
2.10
2.00
7.25 7.20 7.15 7.10 7.05
2.10
4.15
9.0 8.5 8.0 7.5 7.0 6.5 6.0 5.5 5.0 4.5 4.0 3.5 3.0 2.5 2.0 1.5 1.0 0.5 0.0 -0.5

^{13}C NMR, 125 MHz

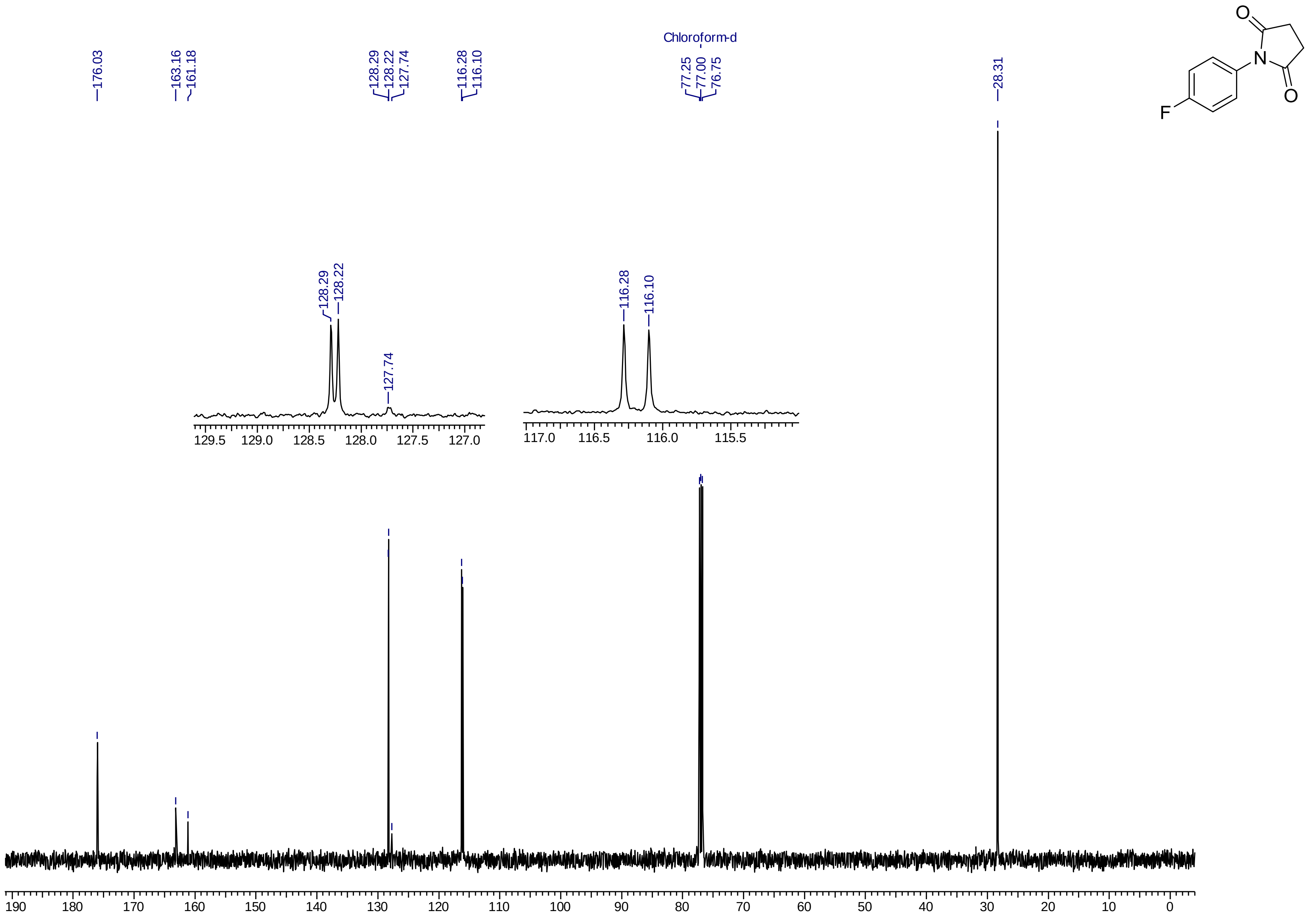

^{13}C NMR, DEPT, 125 MHz

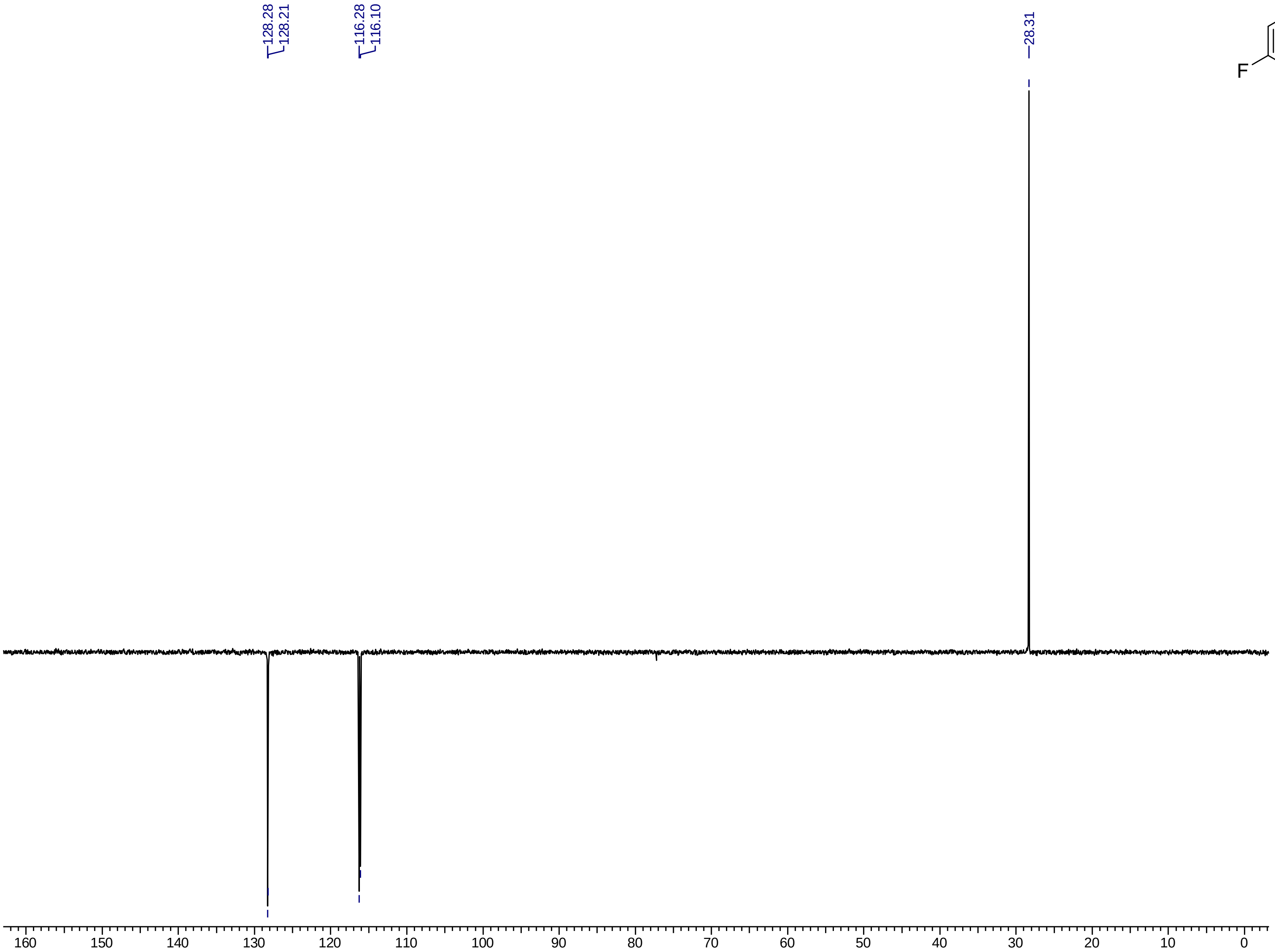

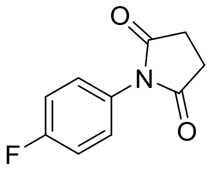

^{1}H NMR, 400 MHz

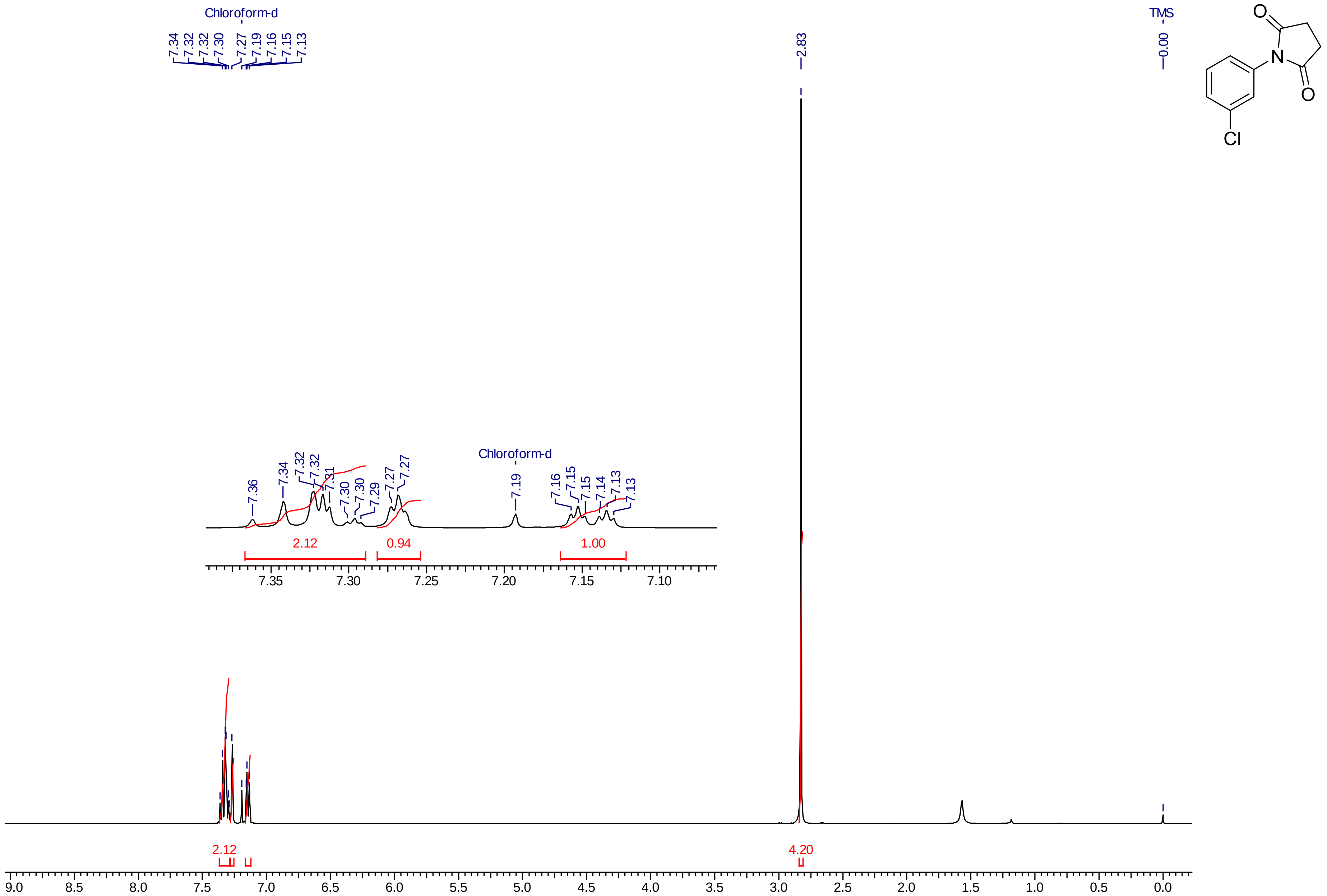

^{13}C NMR, 100 MHz

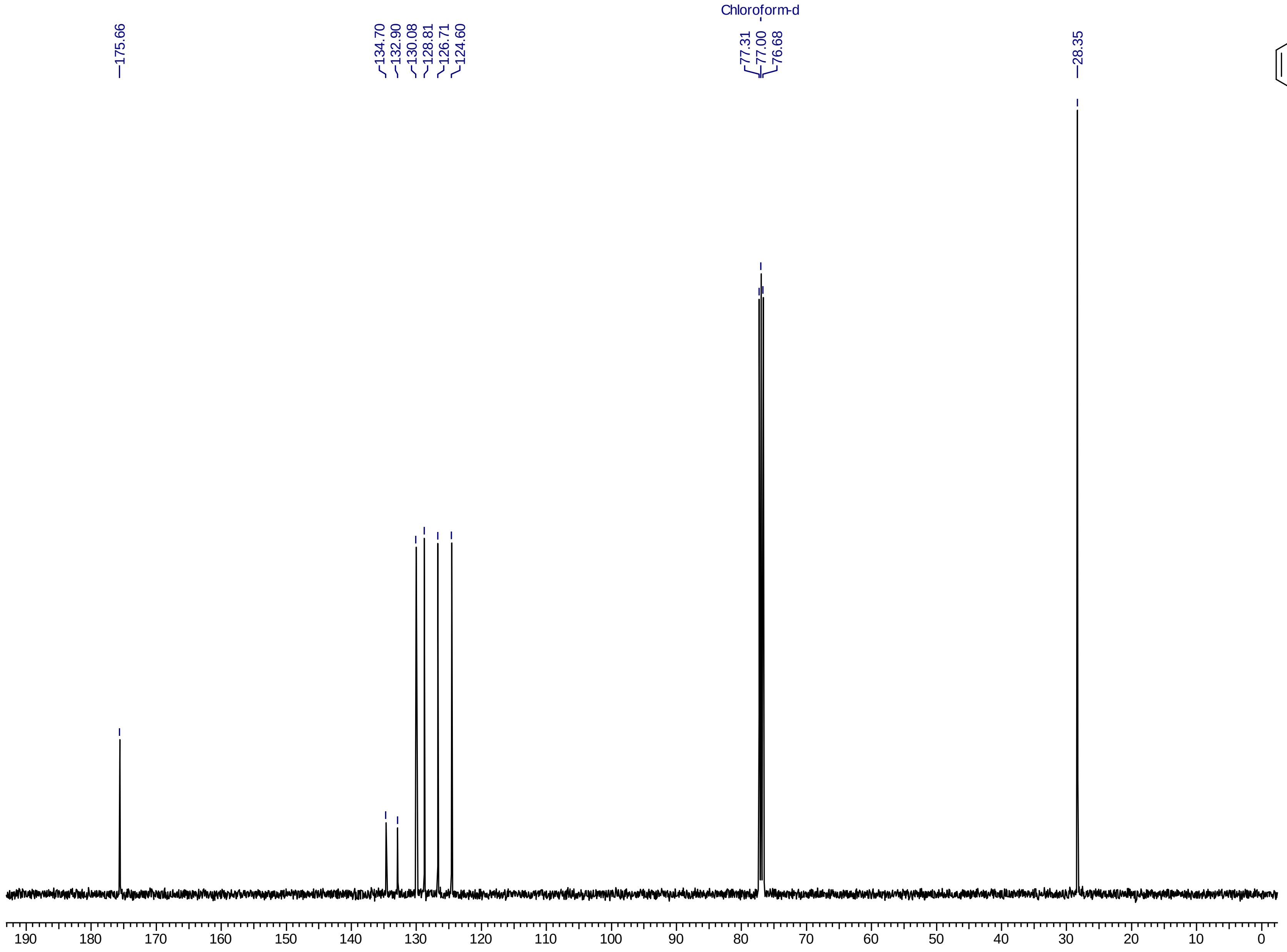

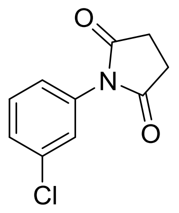

^{13}C NMR, DEPT, 100 MHz

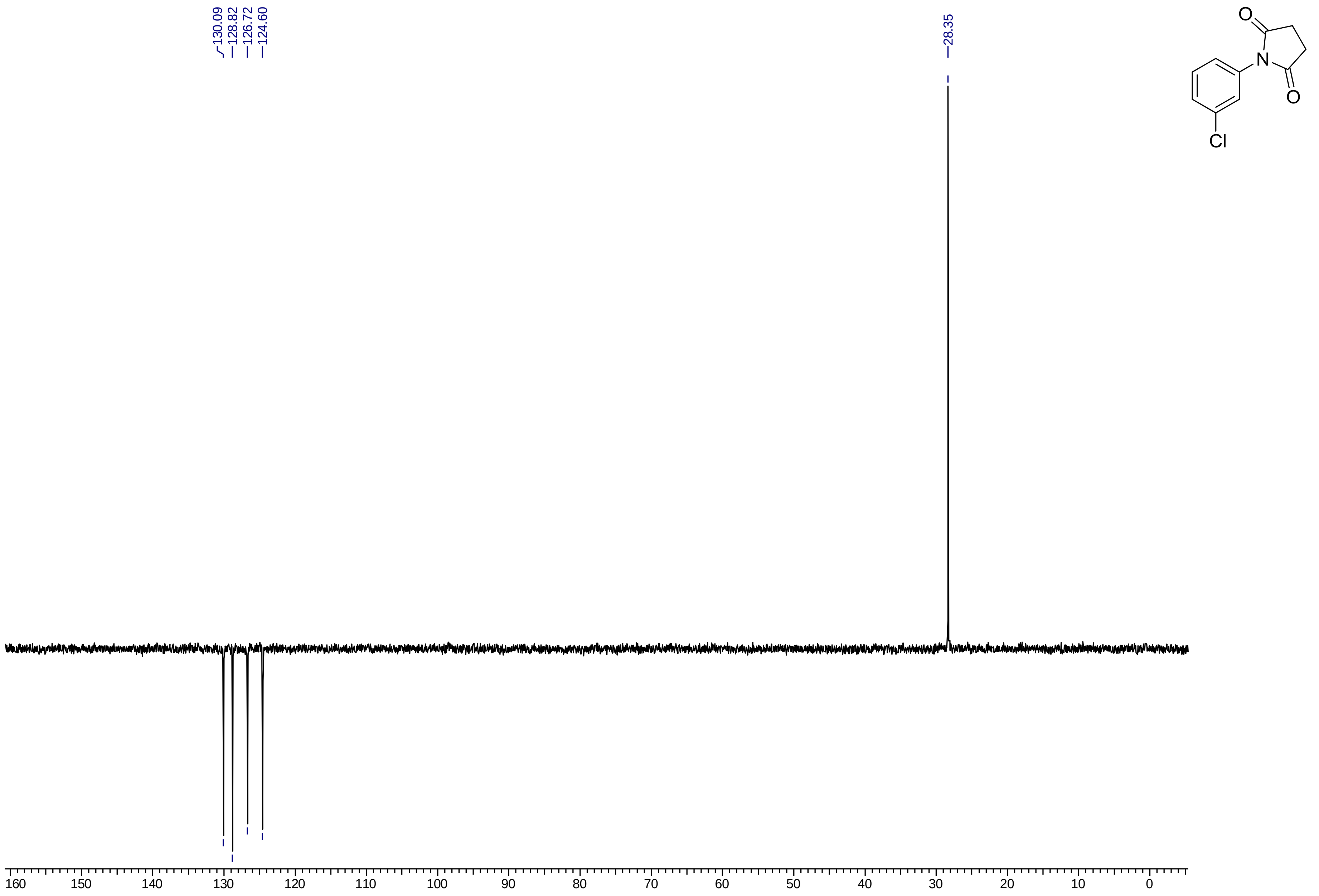

^{1}H NMR, 400 MHz

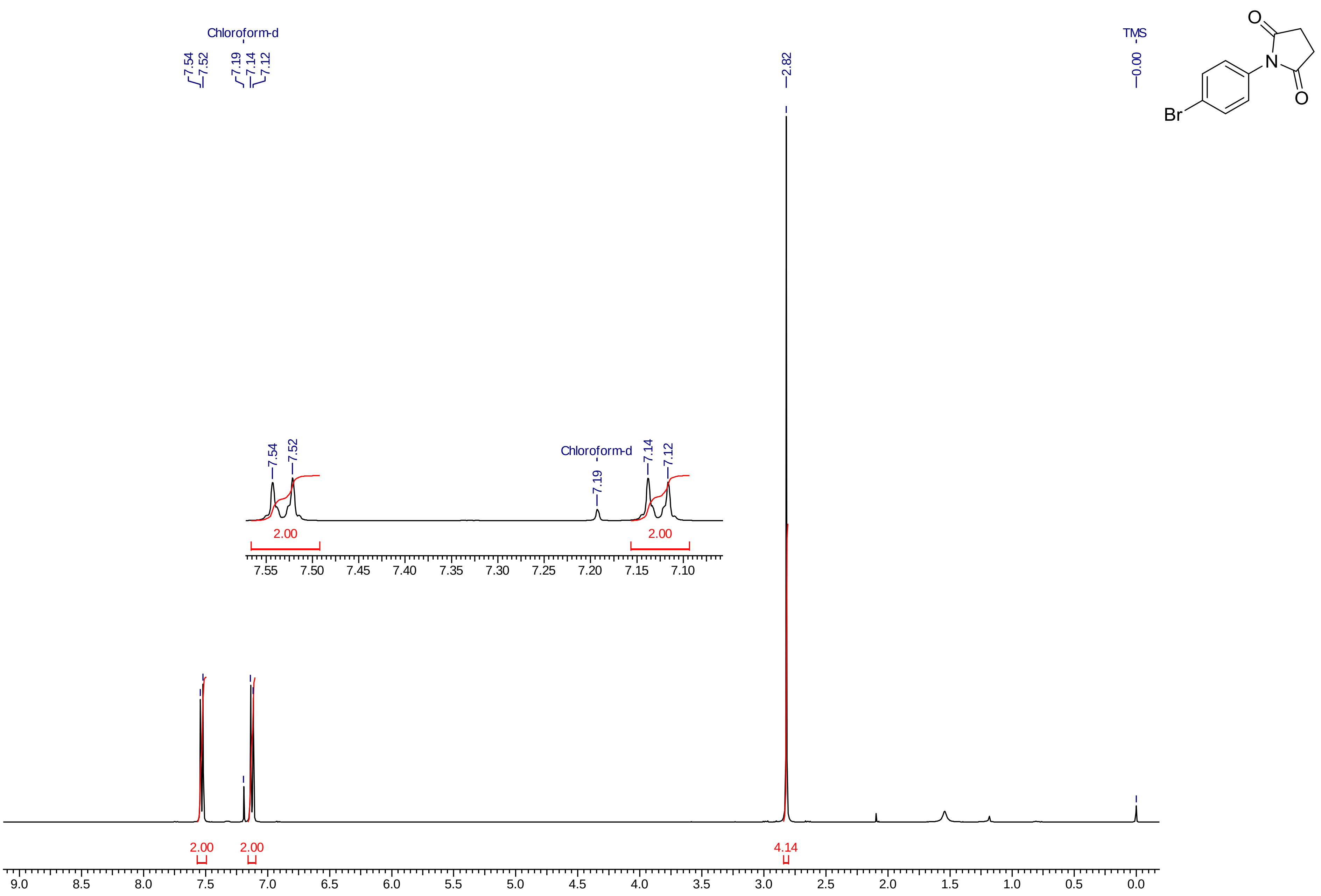

^{13}C NMR, 100 MHz

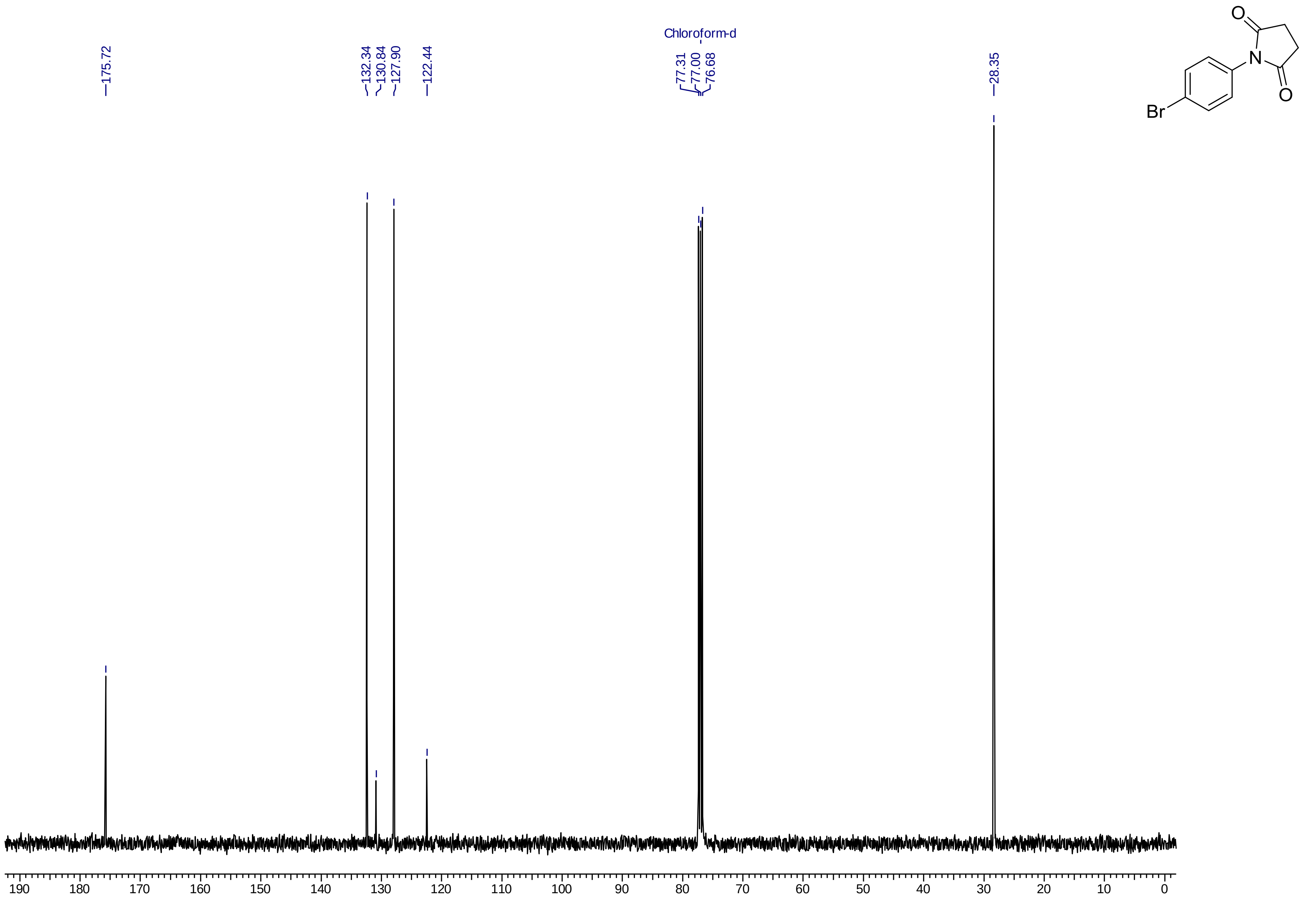

^{13}C NMR, DEPT, 100 MHz

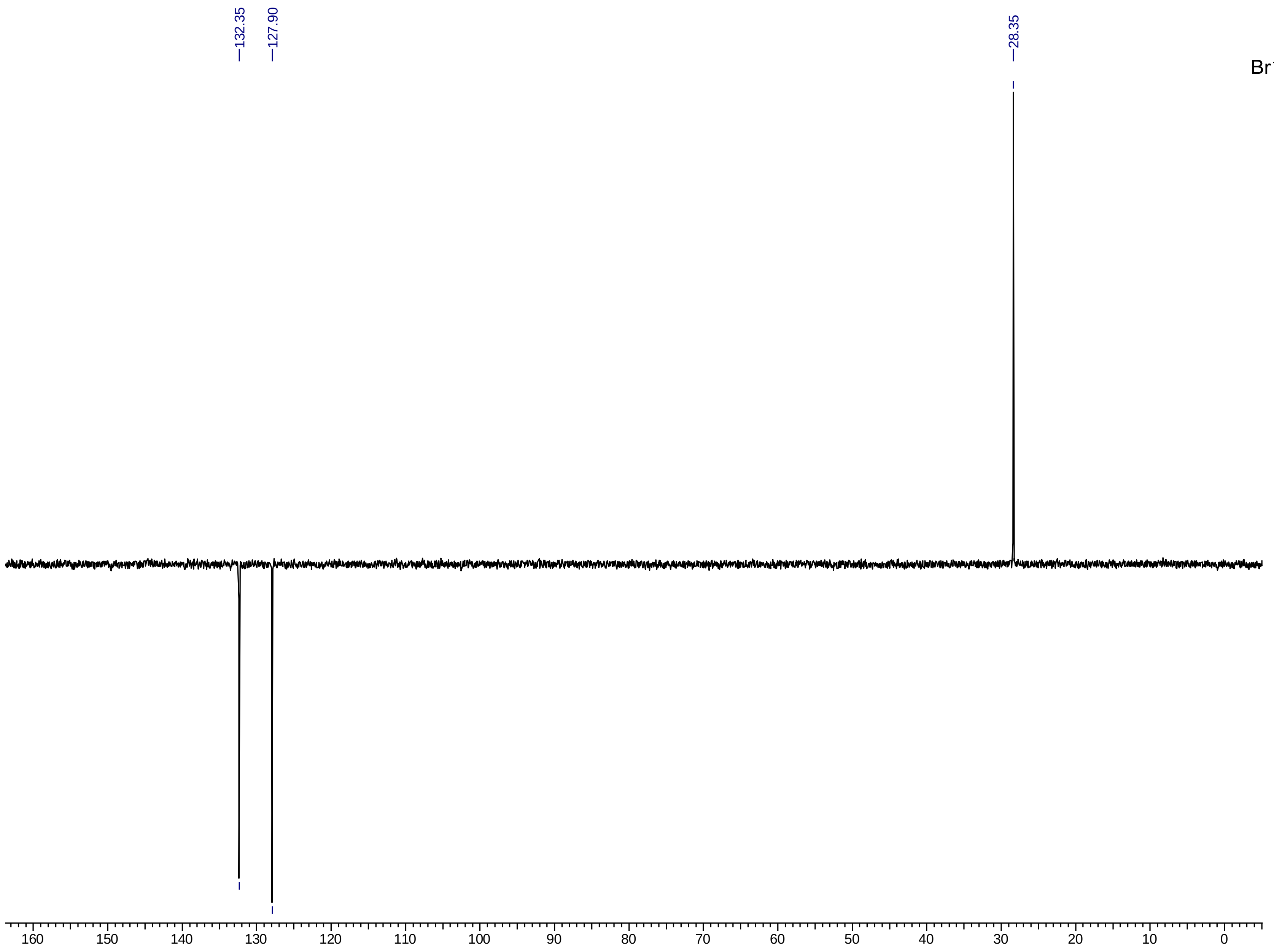

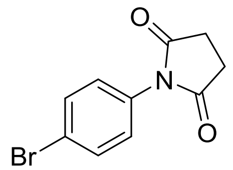

^{1}H NMR, 400 MHz

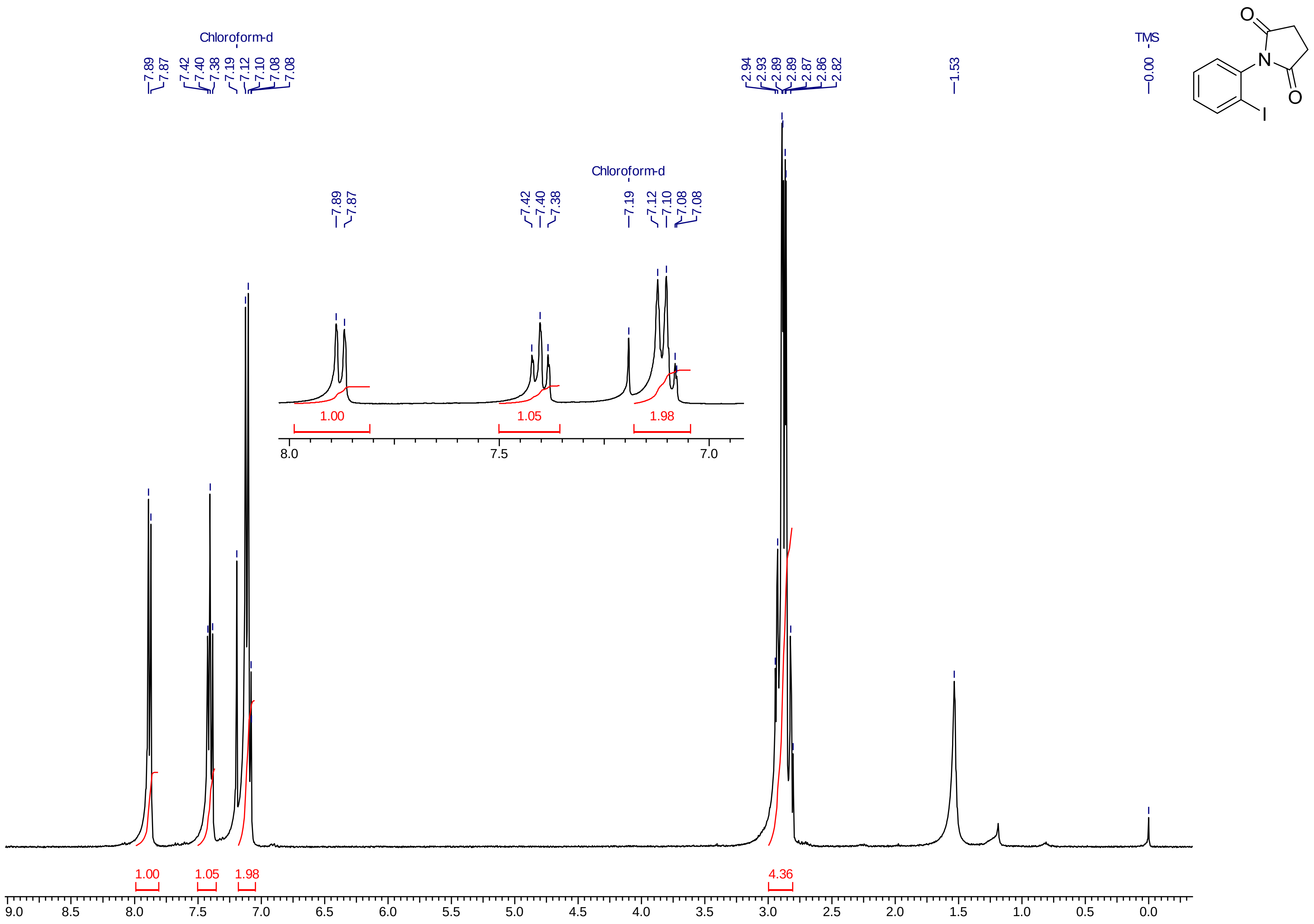

^{13}C NMR, 100 MHz

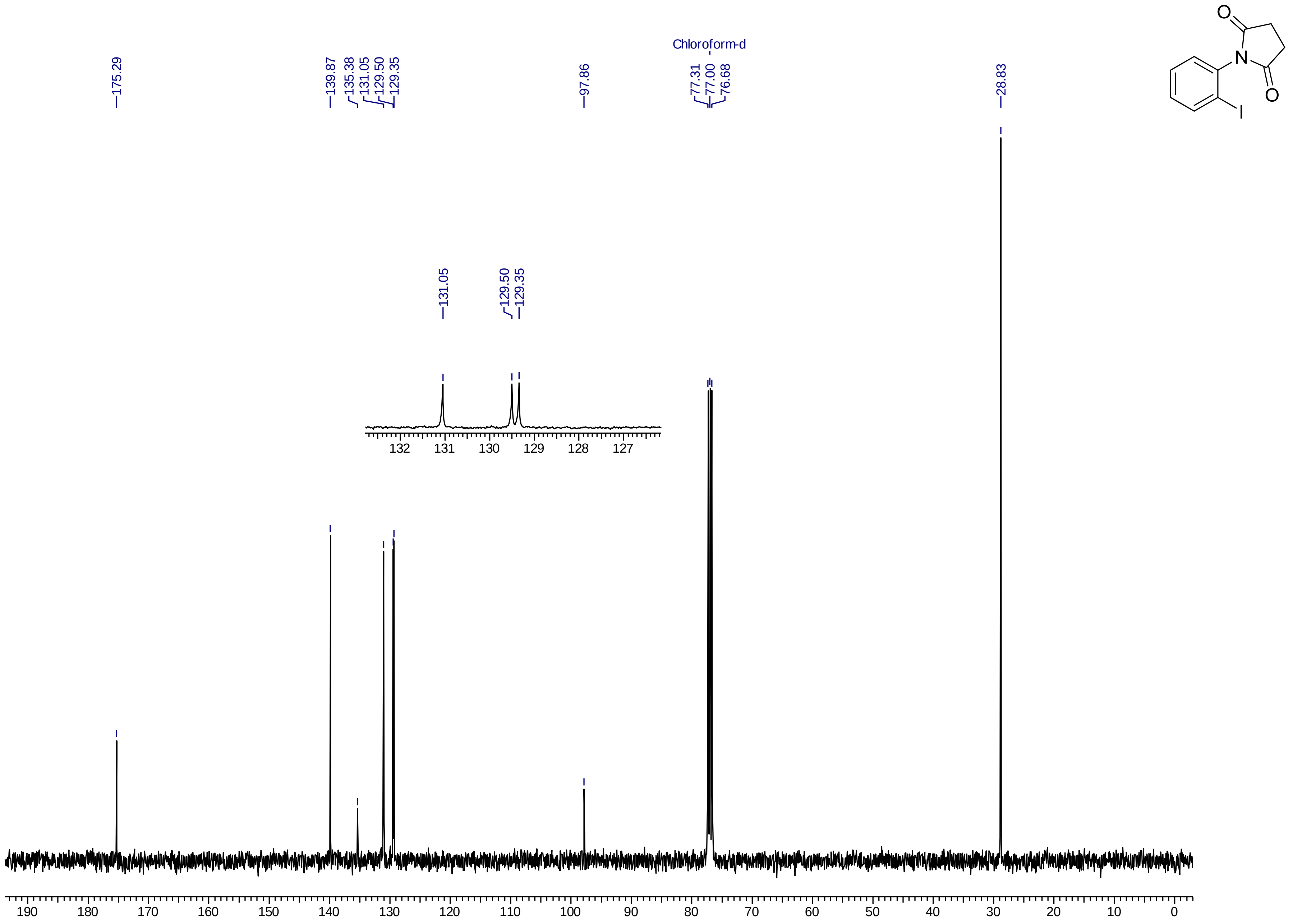

^{13}C NMR, DEPT, 100 MHz

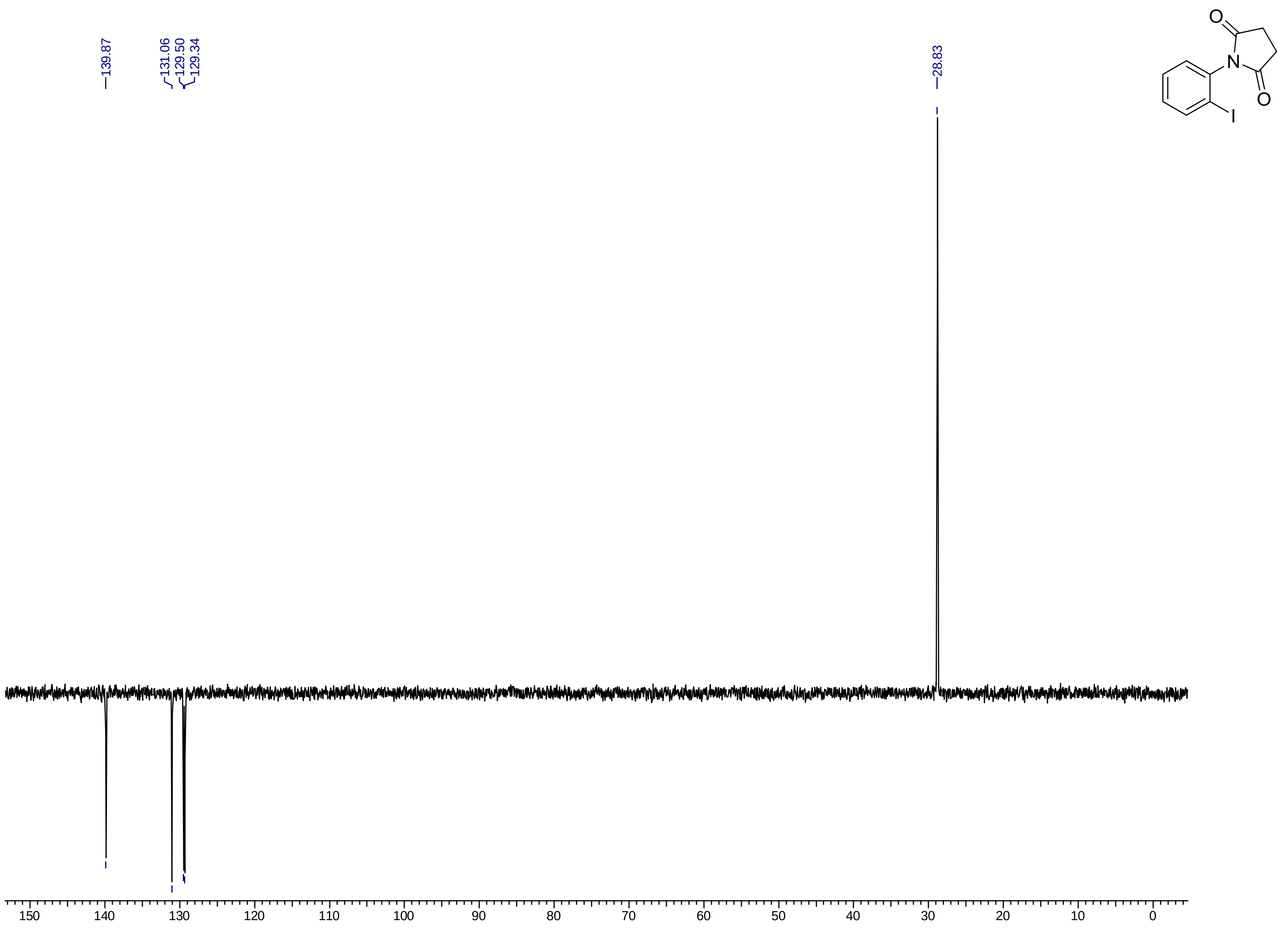

^{1}H NMR, 400 MHz

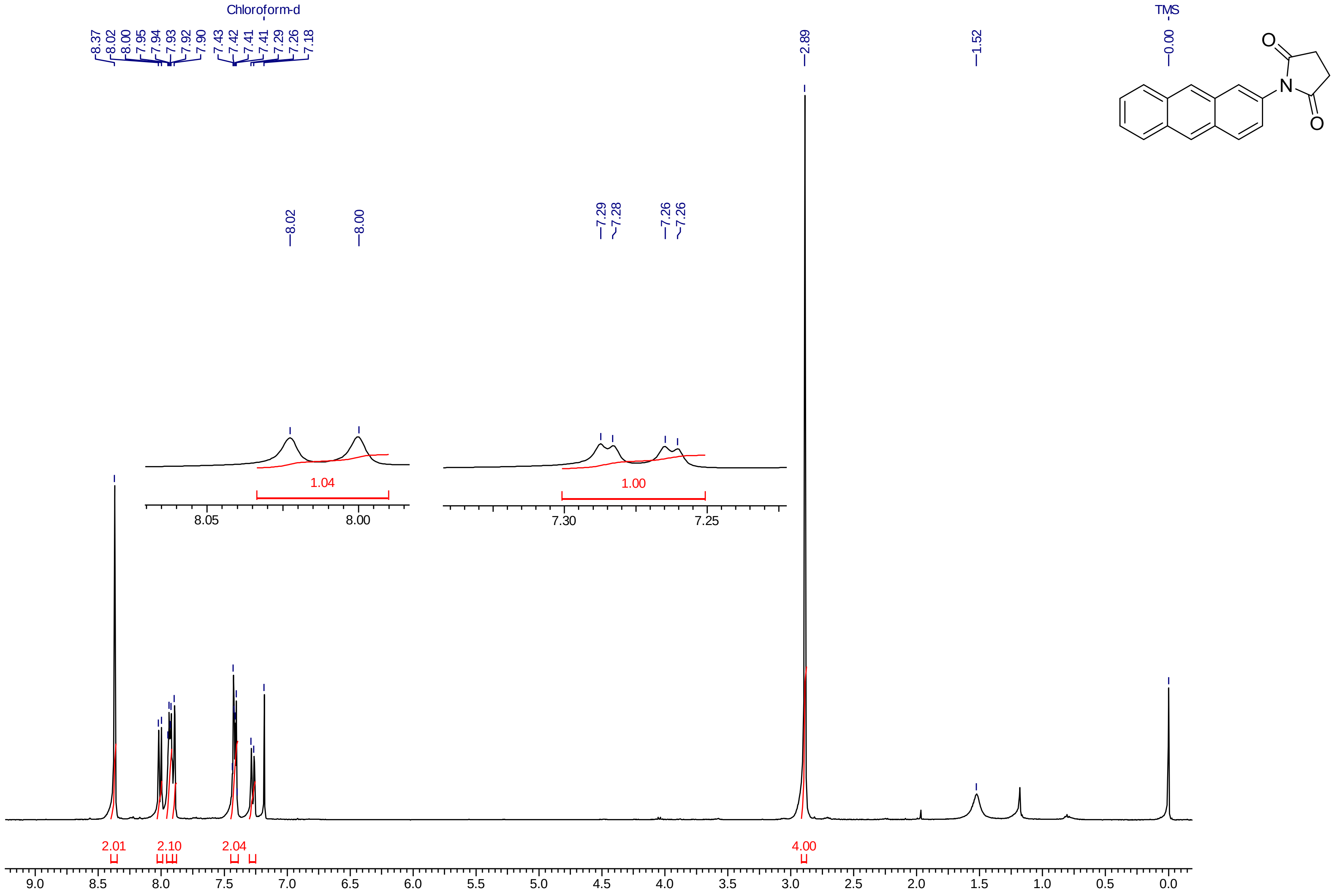

^{13}C NMR, 100 MHz

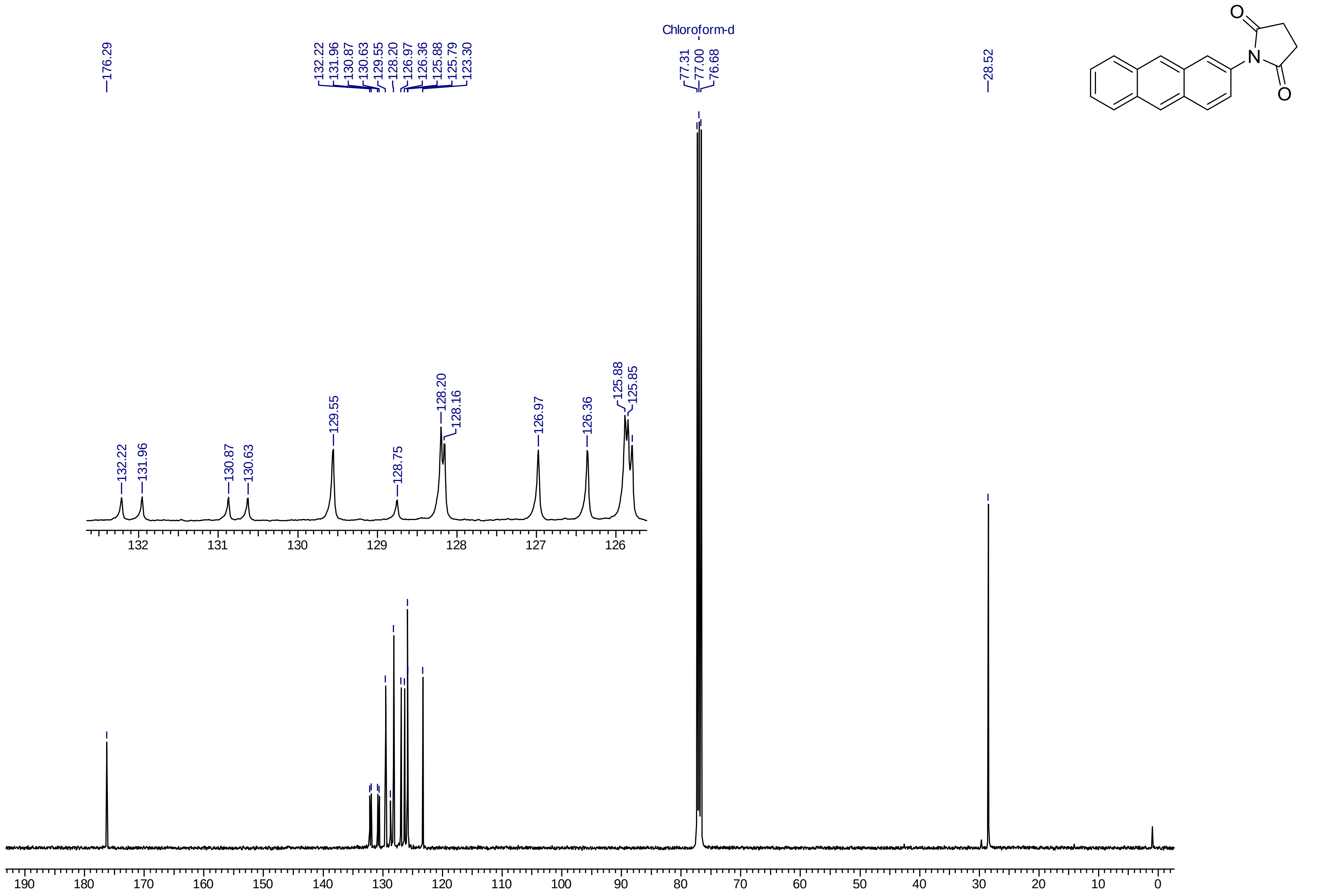

^{13}C NMR, DEPT, 100 MHz

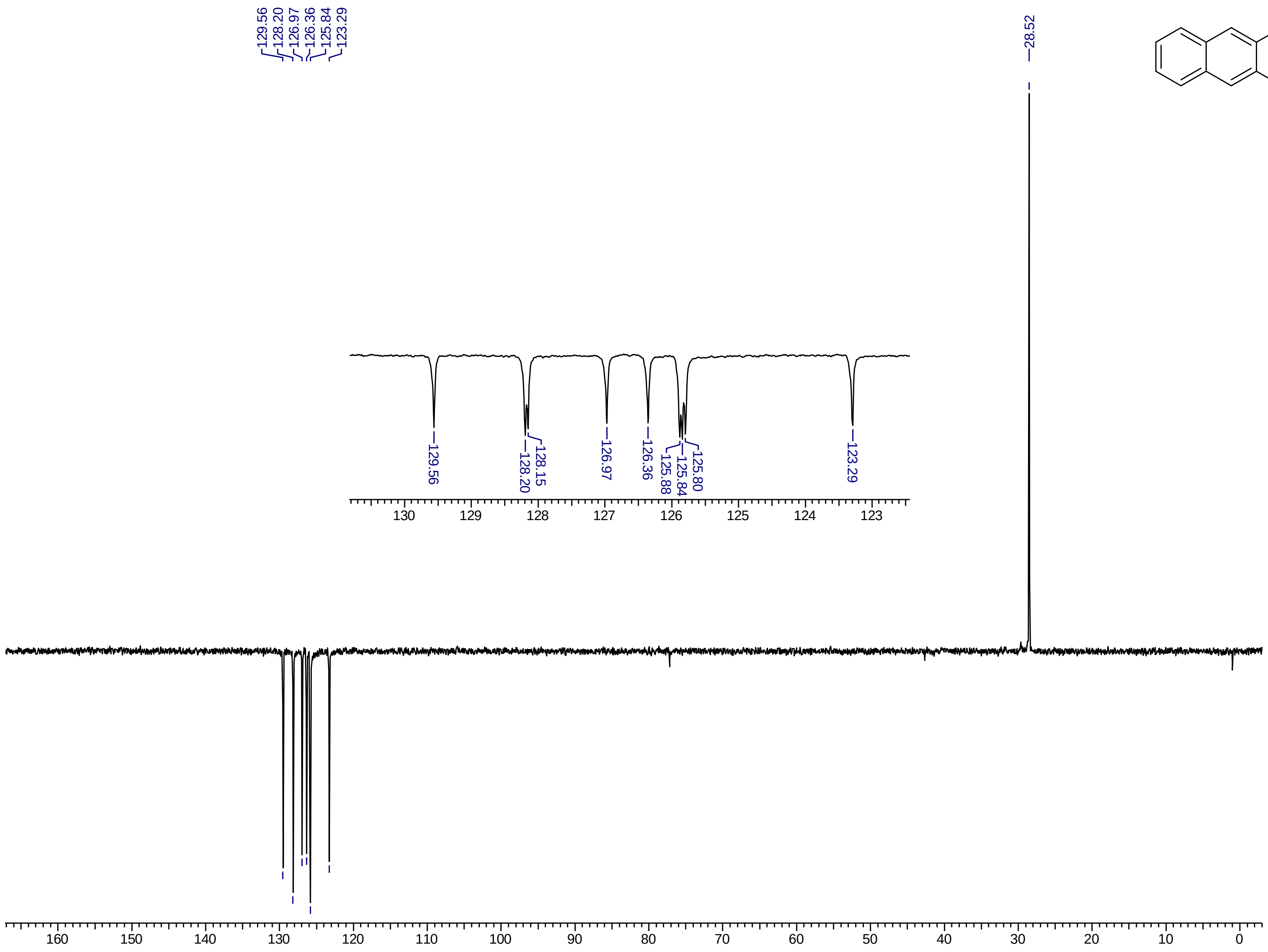

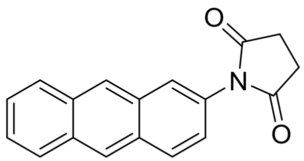

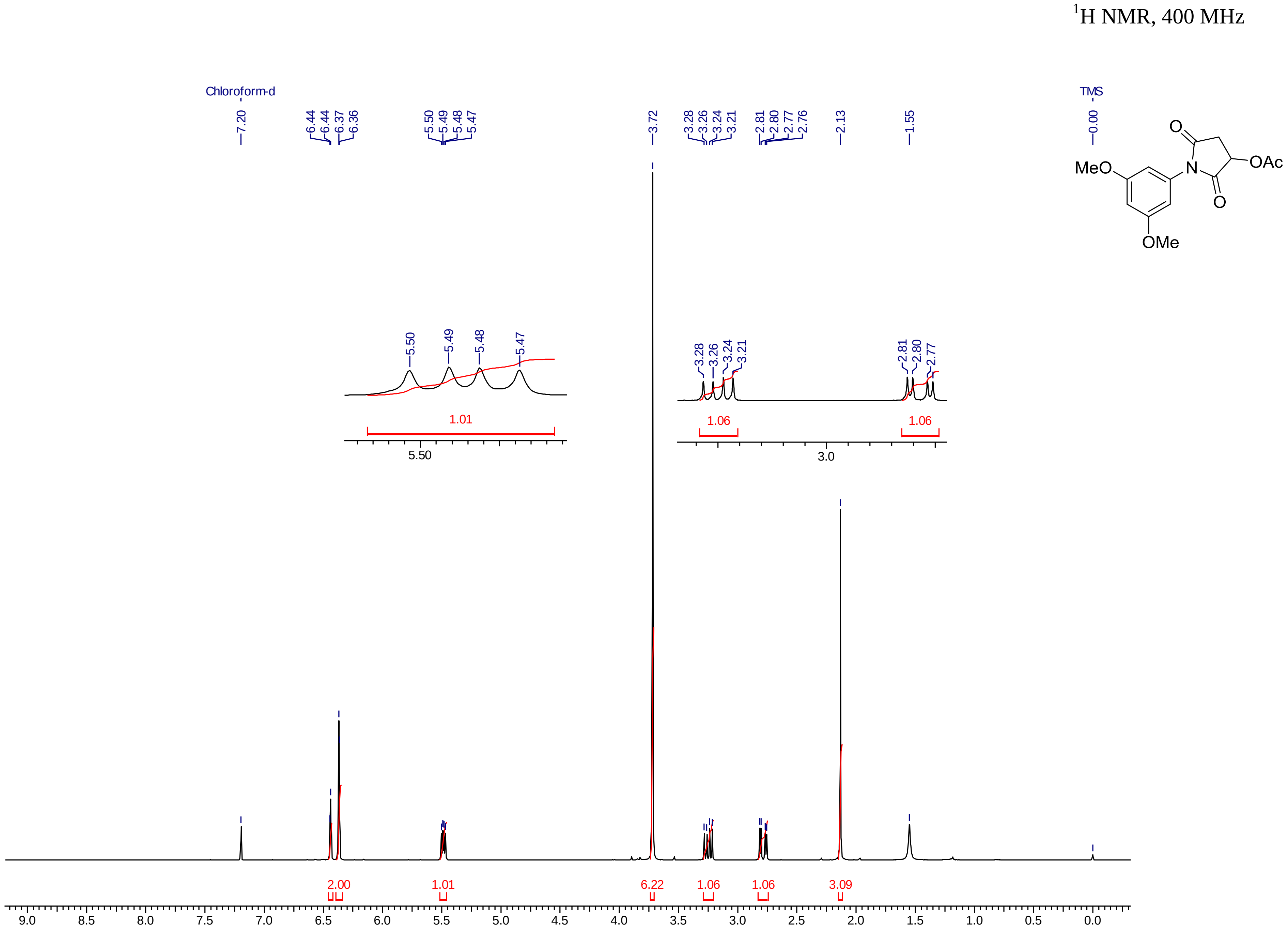

1H NMR, 400 MHz
Chloroform-d
7.20
6.44
6.44
6.37
6.36
5.50
5.49
5.48
5.47
3.72
3.28
3.26
3.24
3.21
2.81
2.80
2.77
2.76
2.13
1.55
TMS
0.00
MeO
OMe
N
O
O
OAc
1.01
1.06
1.06
2.00
1.01
6.22
1.06
1.06
3.09

^{13}C NMR, 100 MHz

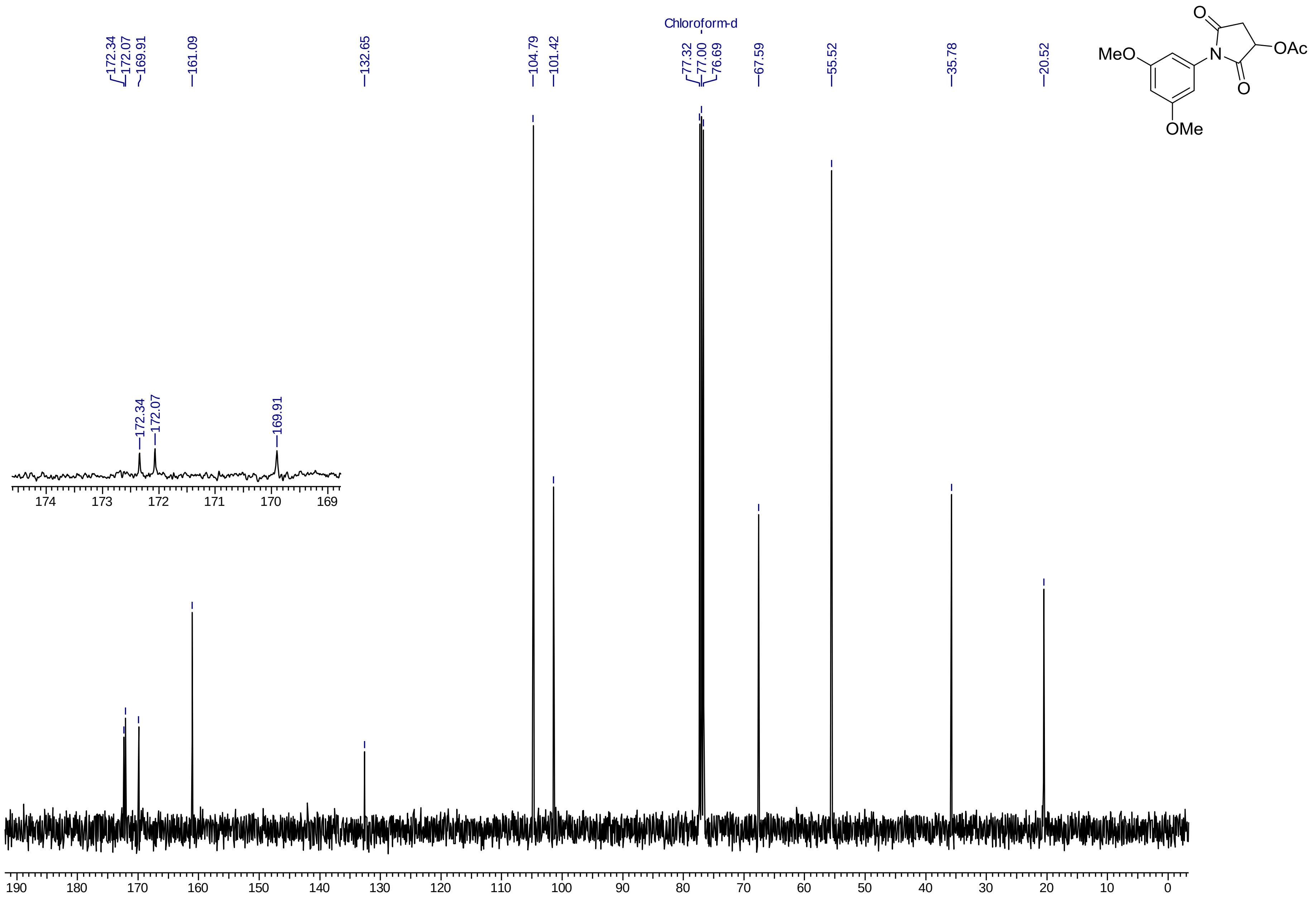

^{13}C NMR, DEPT, 100 MHz

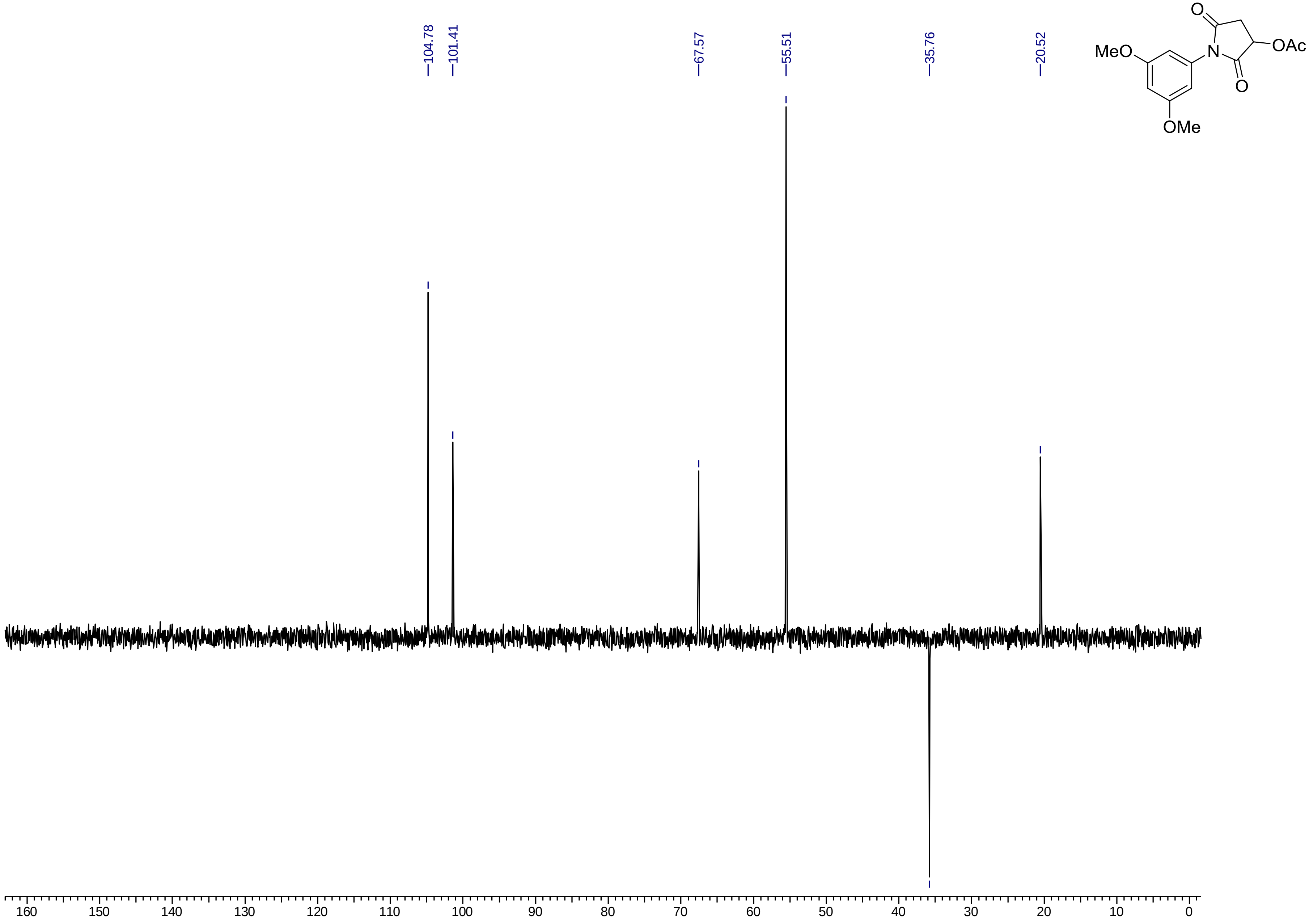

^{1}H NMR, 400 MHz

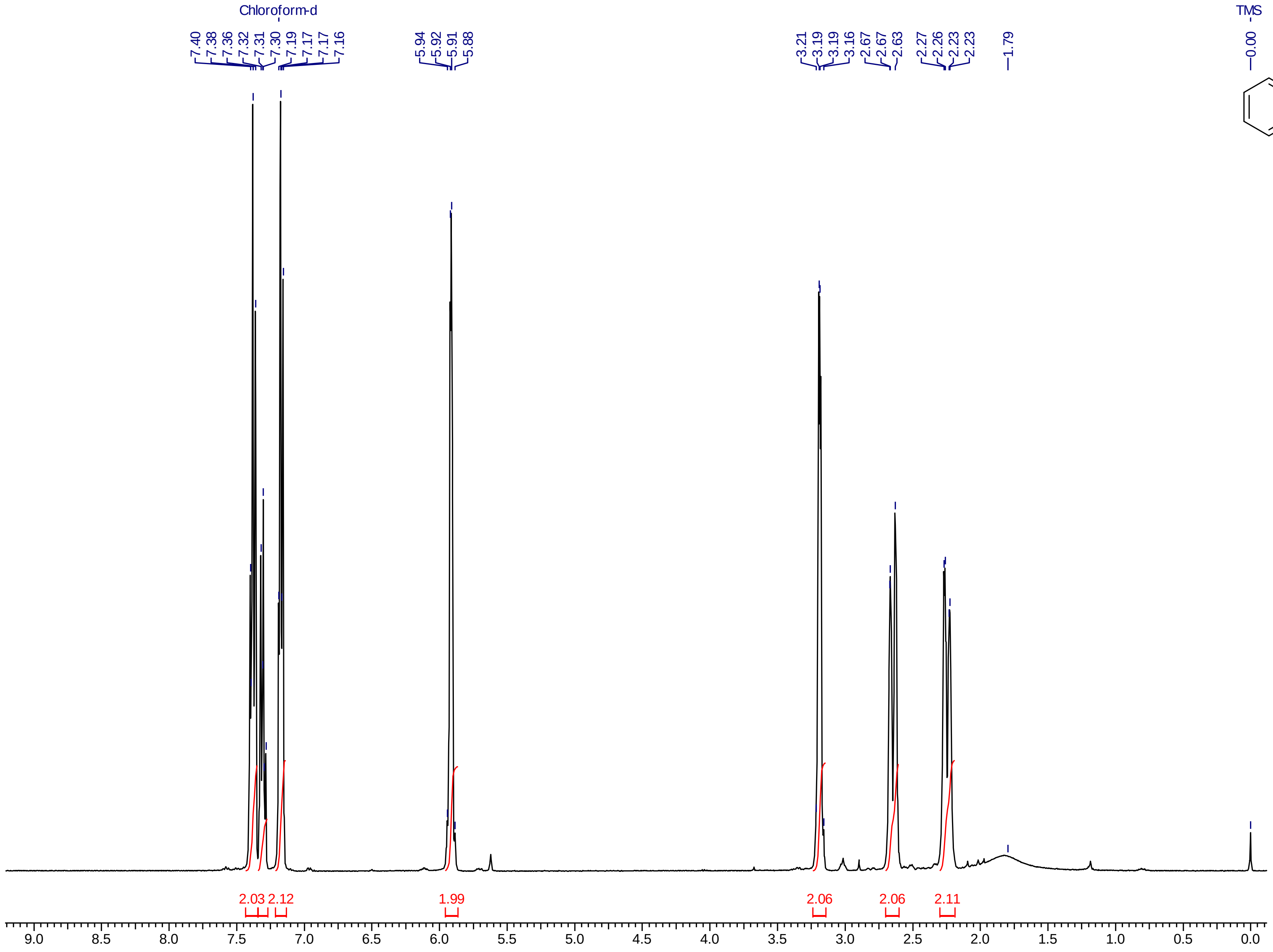

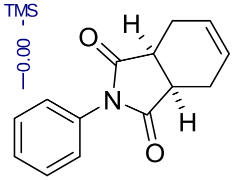

^{13}C NMR, 100 MHz

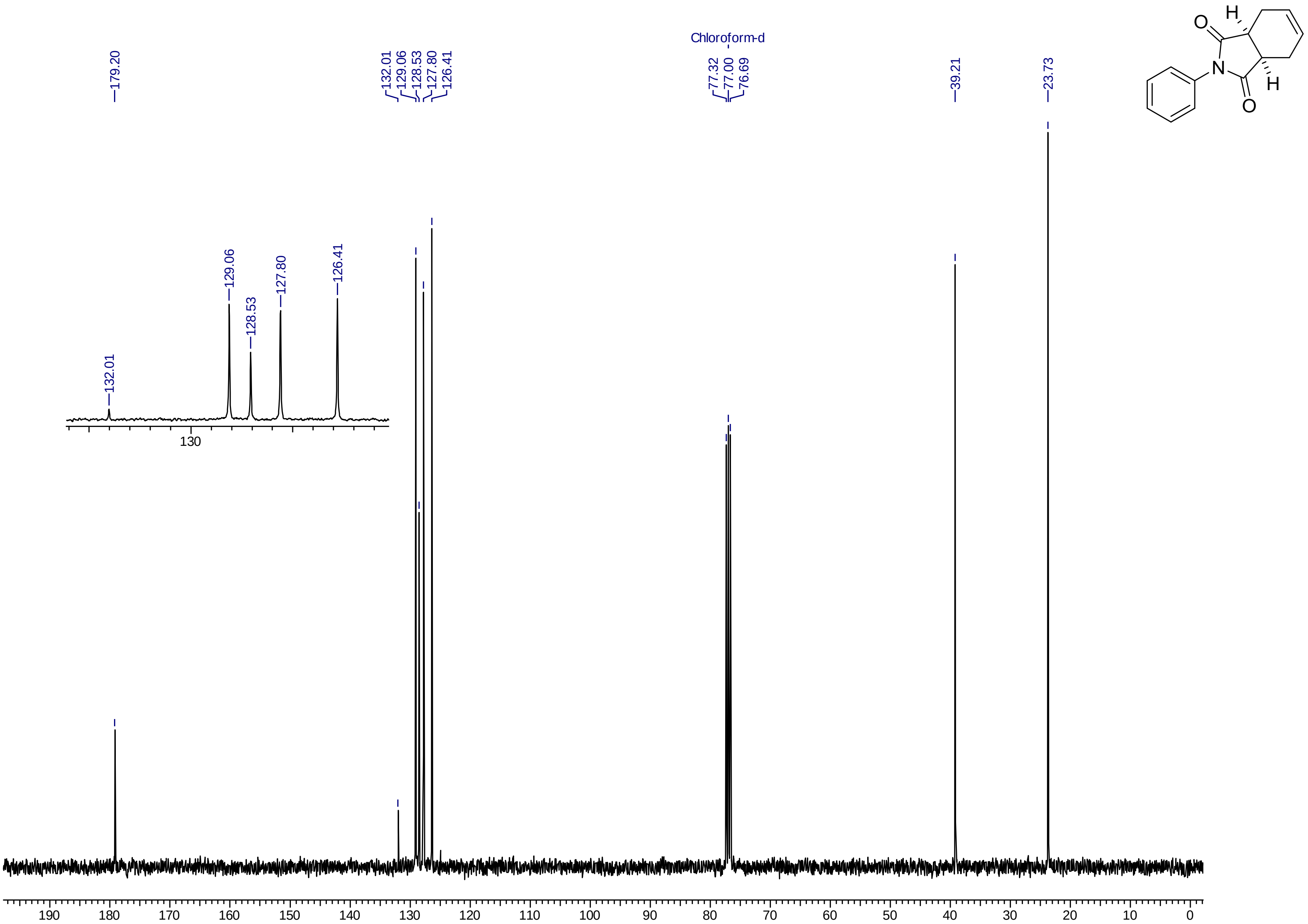

^{13}C NMR, DEPT, 100 MHz

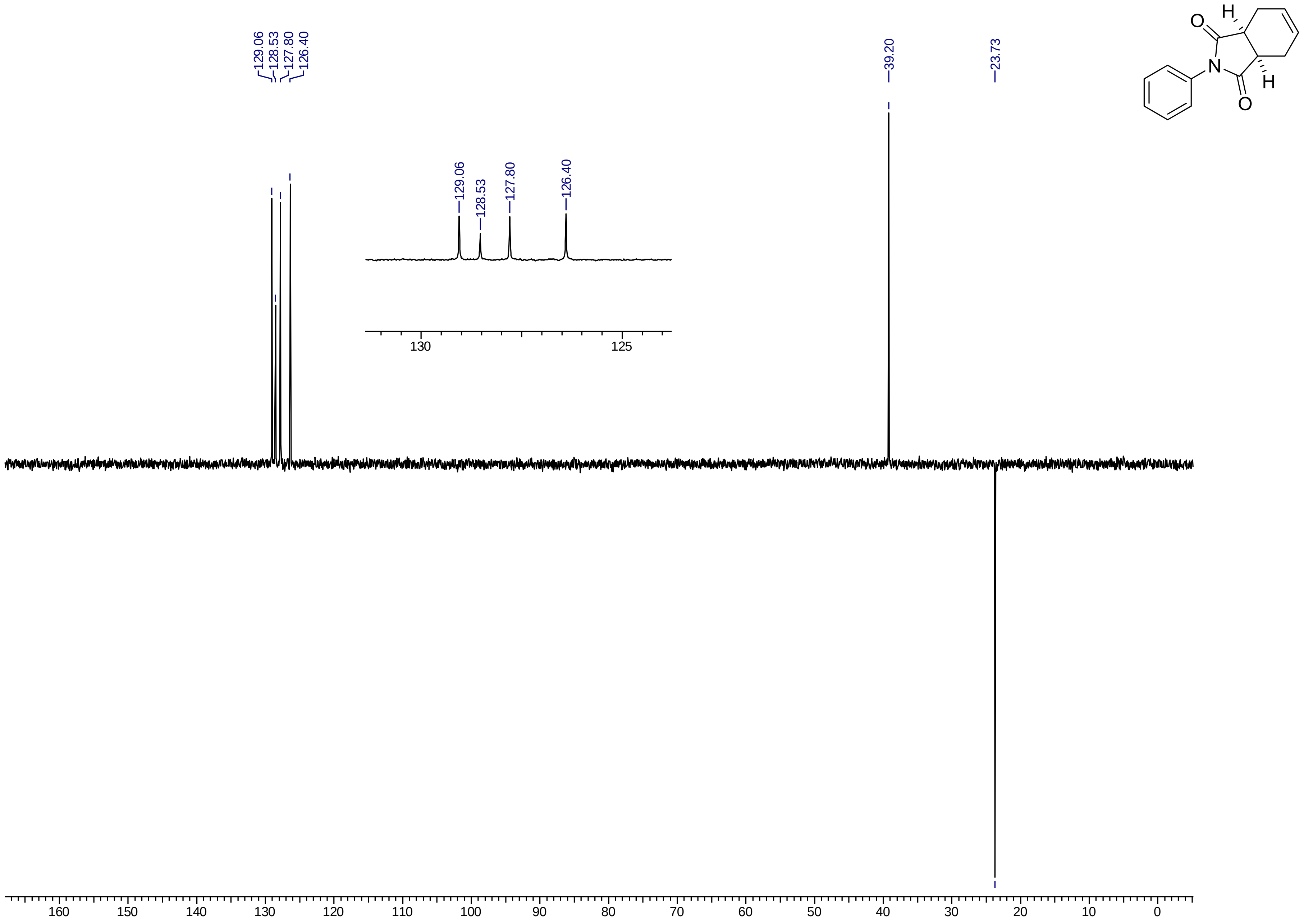

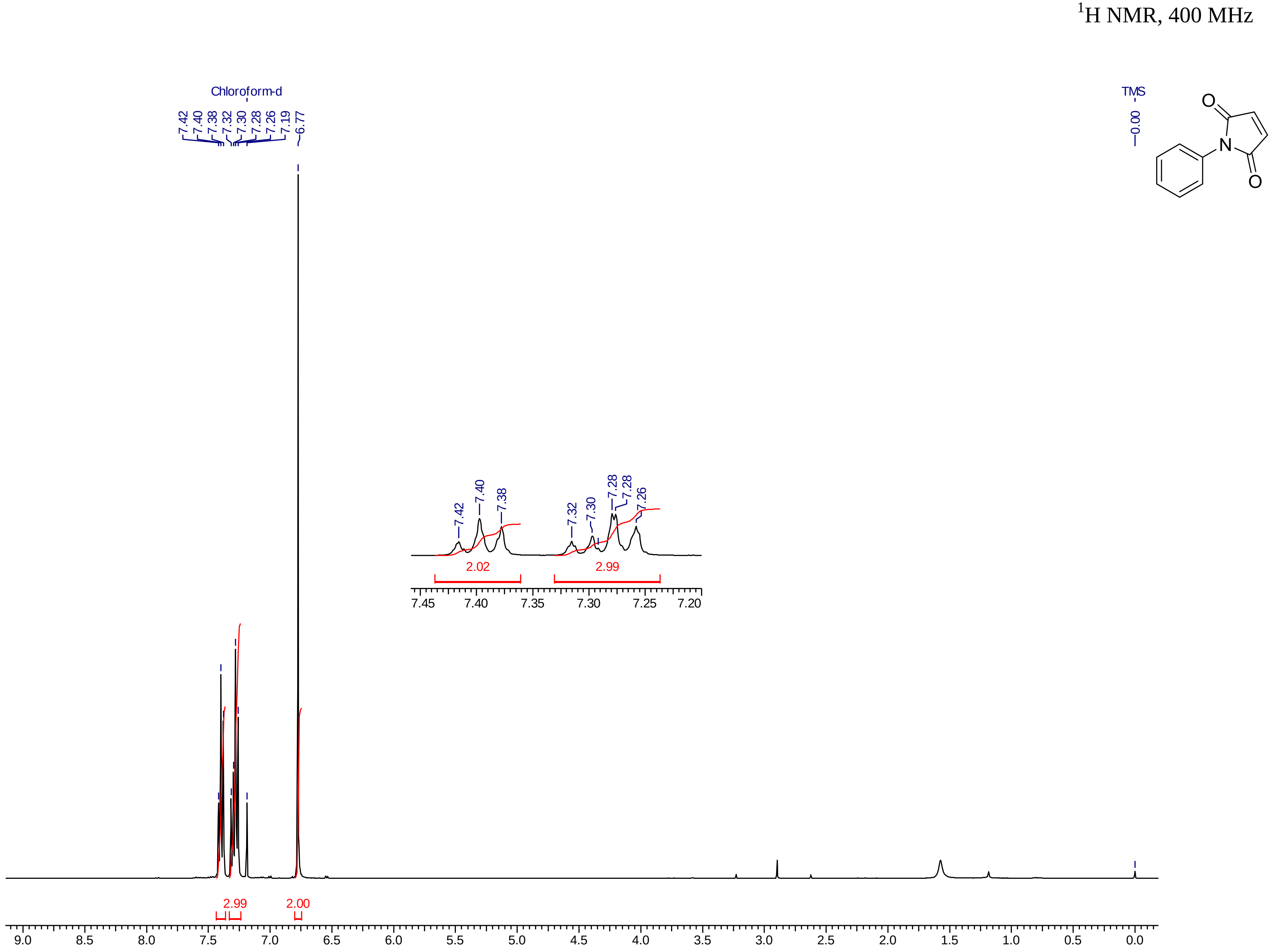

1H NMR, 400 MHz
Chloroform-d
7.42
7.40
7.38
7.32
7.30
7.28
7.26
7.19
6.77
TMS
0.00
2.02
2.99
2.00

^{13}C NMR, 100 MHz

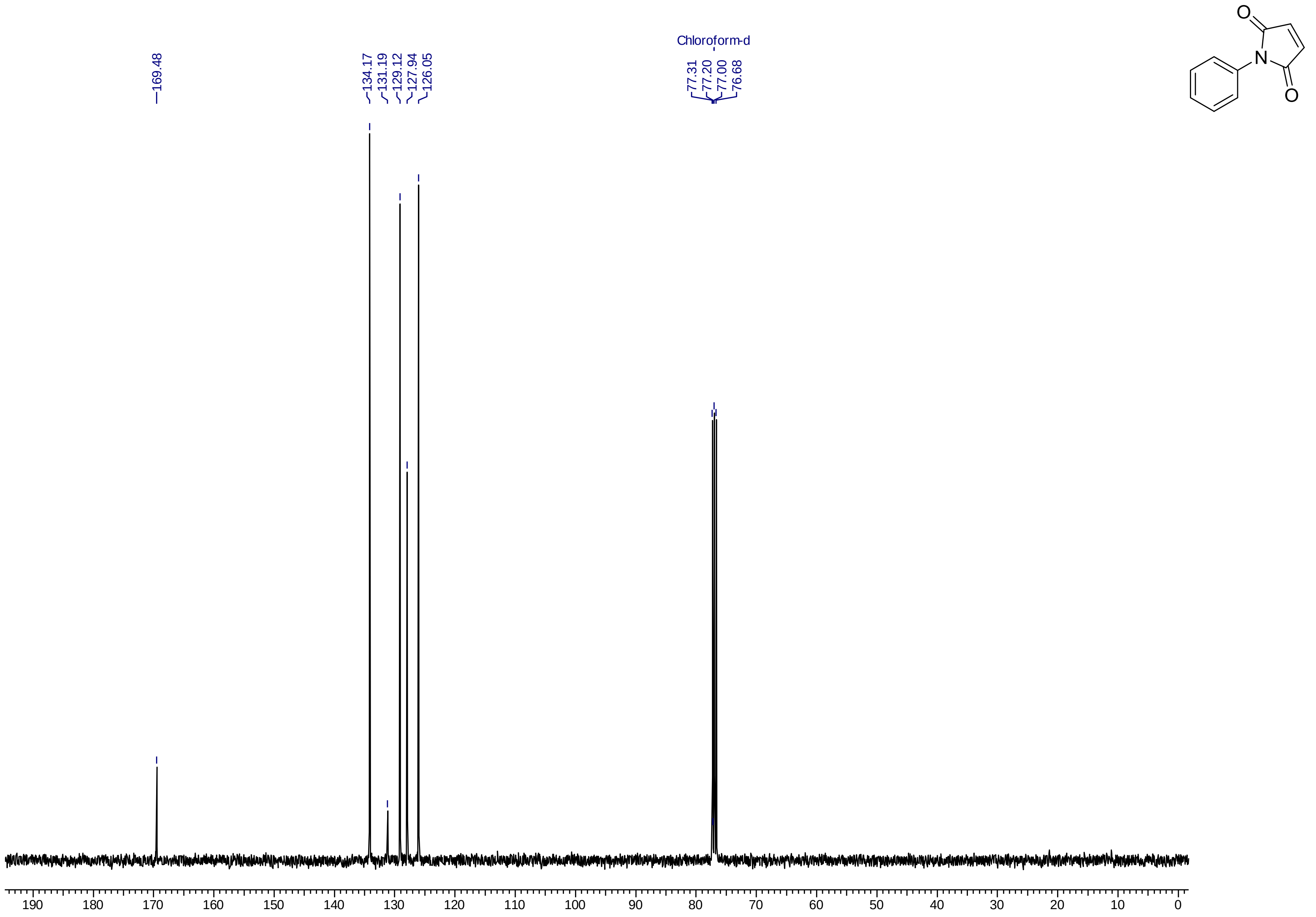

^{13}C NMR, DEPT, 100 MHz

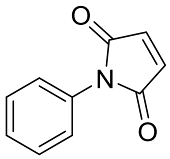

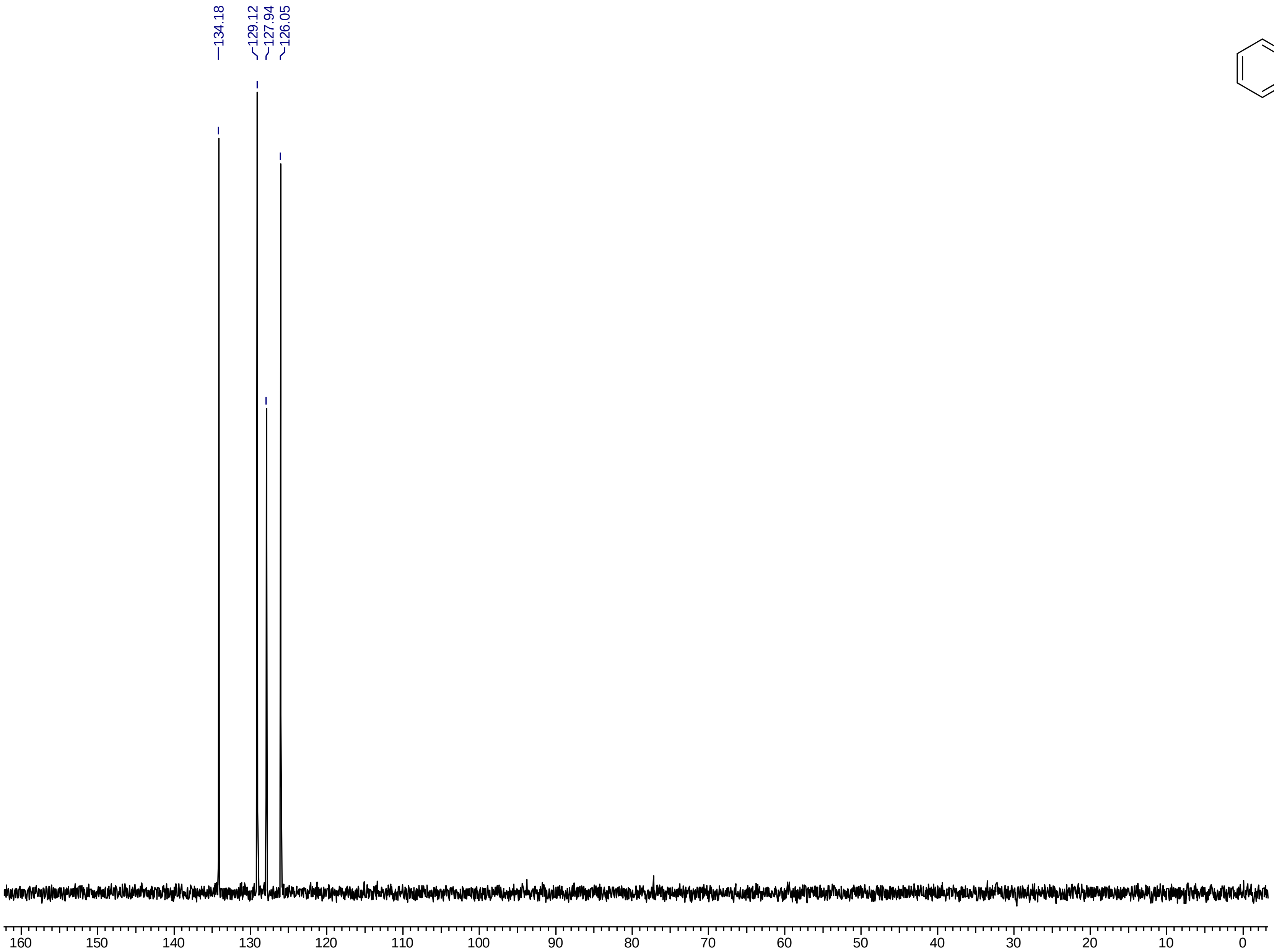

^{1}H NMR, 400 MHz

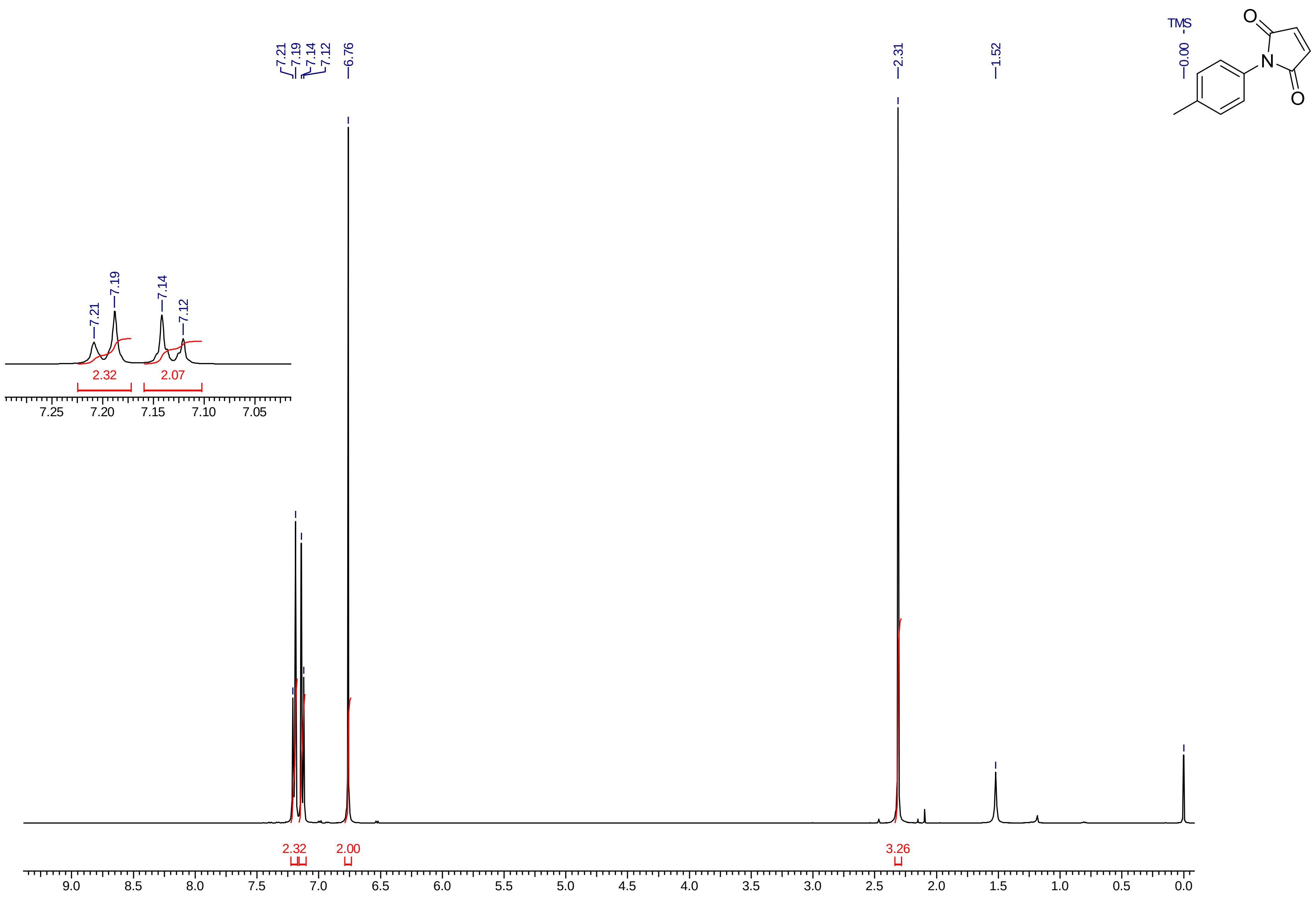

^{13}C NMR, 100 MHz

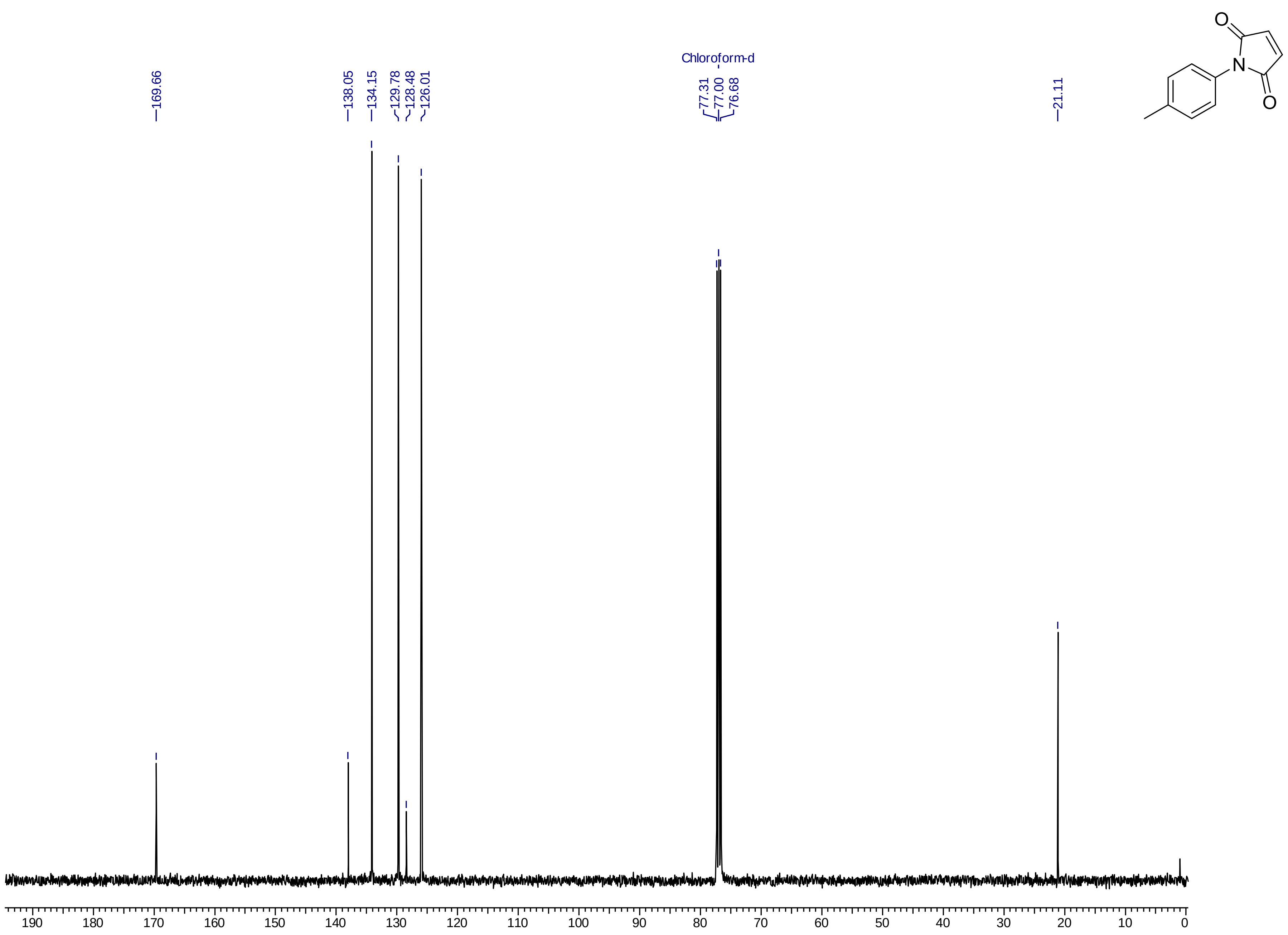

^{13}C NMR, DEPT, 100 MHz

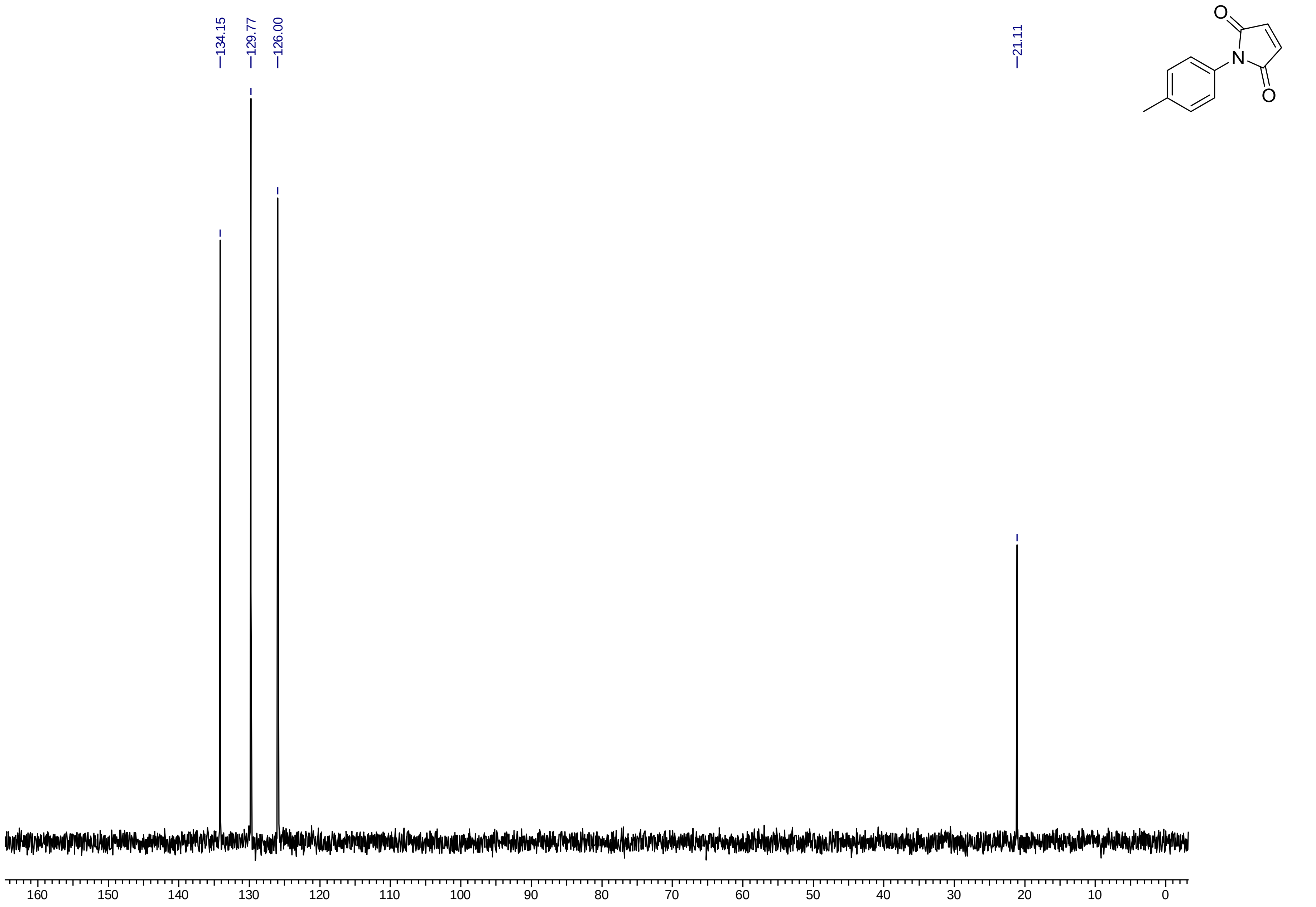

^{1}H NMR, 500 MHz

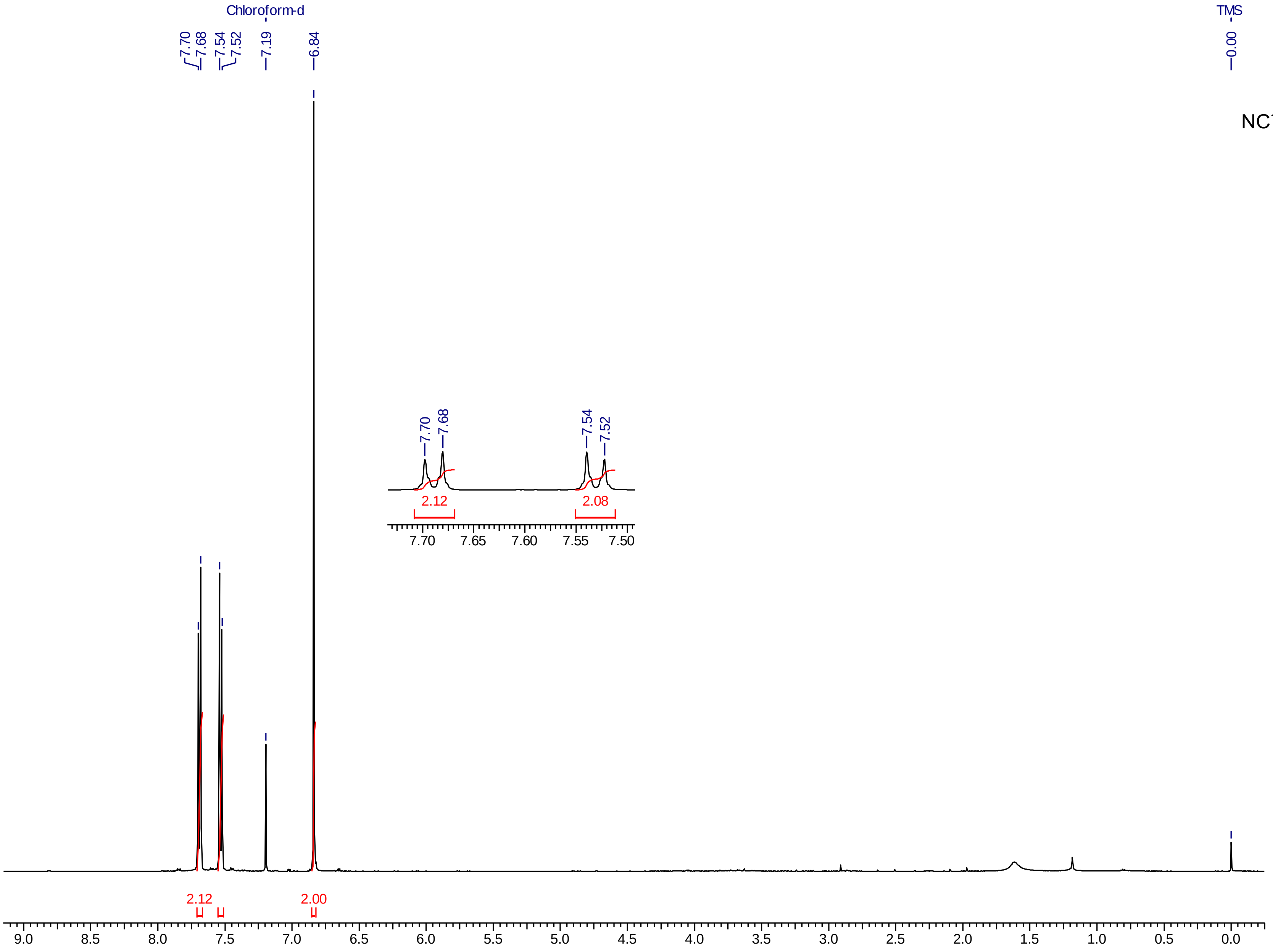

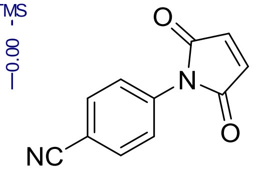

^{13}C NMR, 125 MHz

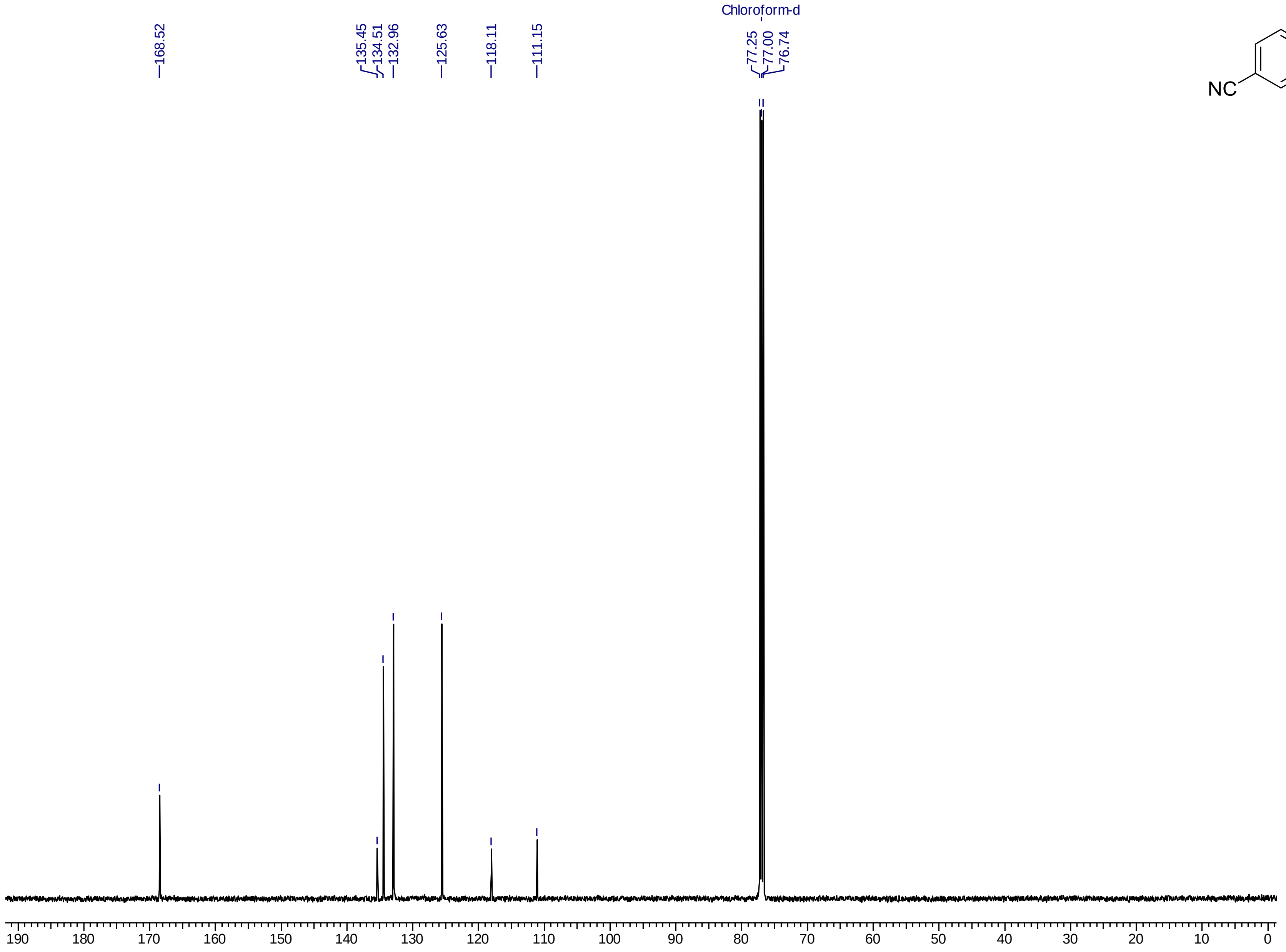

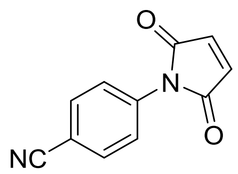

^{13}C NMR, DEPT, 125 MHz

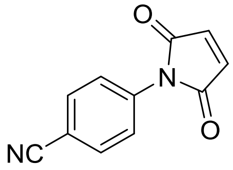

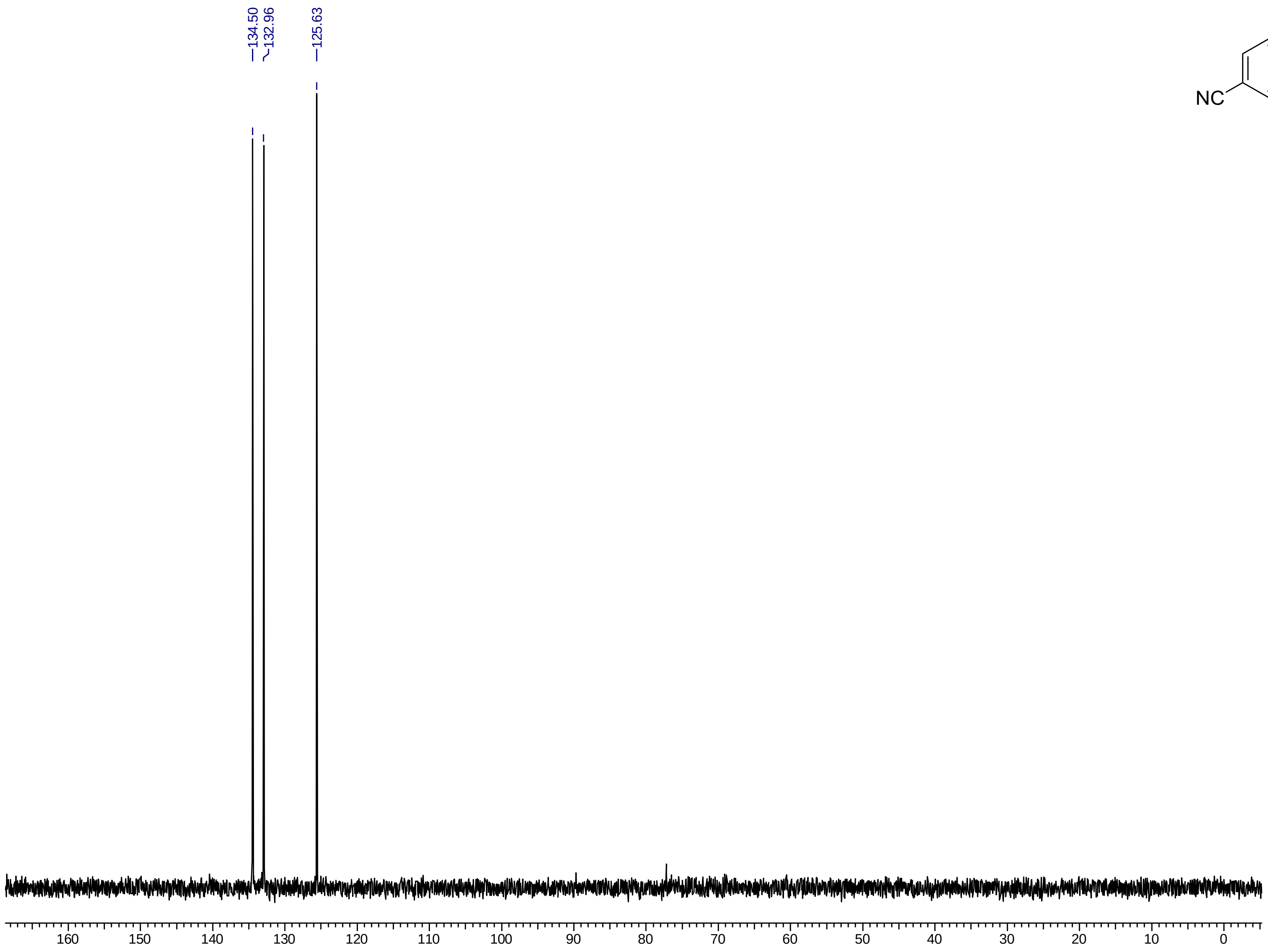

^{1}H NMR, 400 MHz

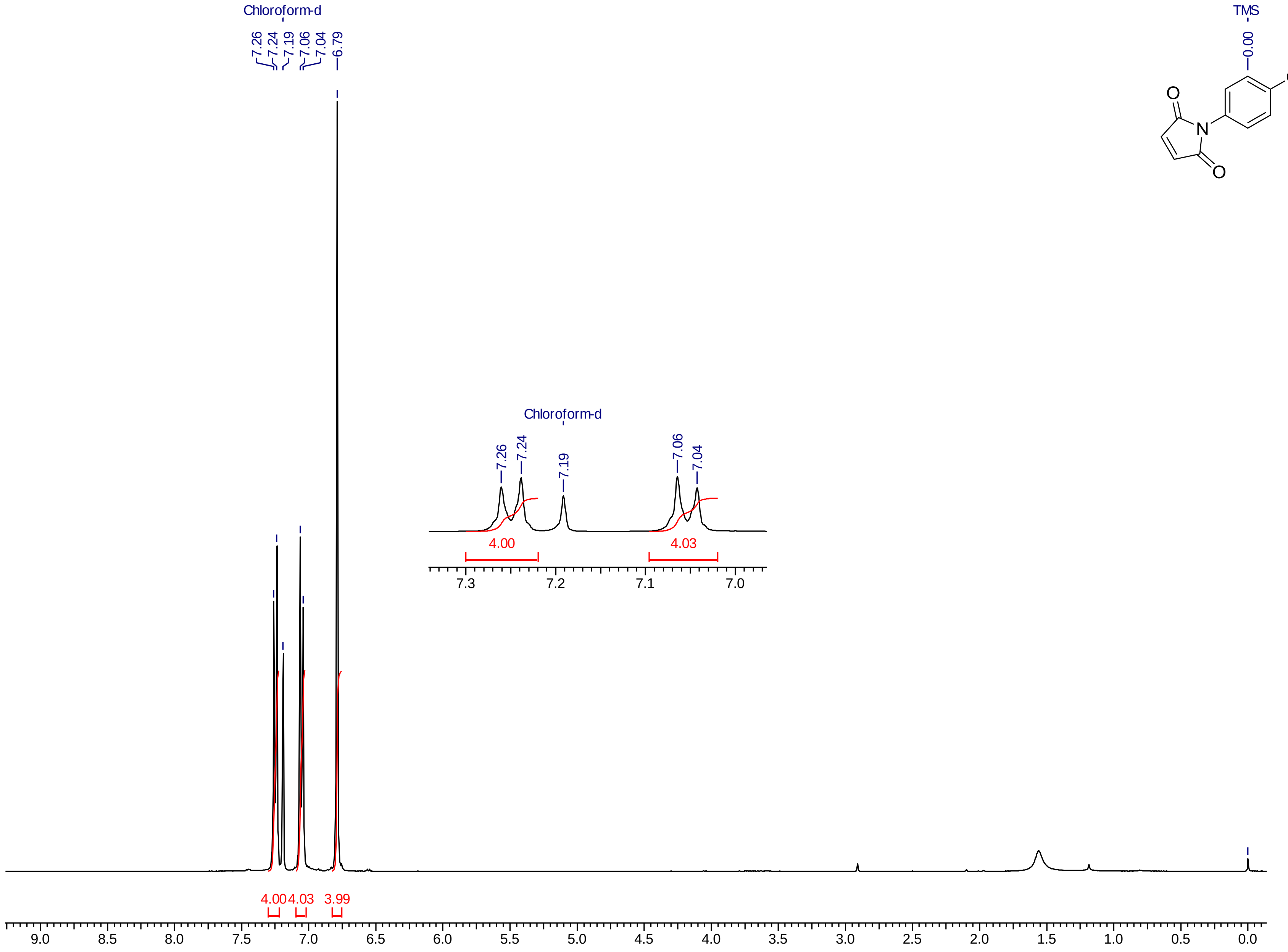

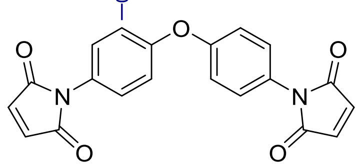

^{13}C NMR, 100 MHz

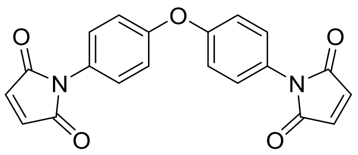

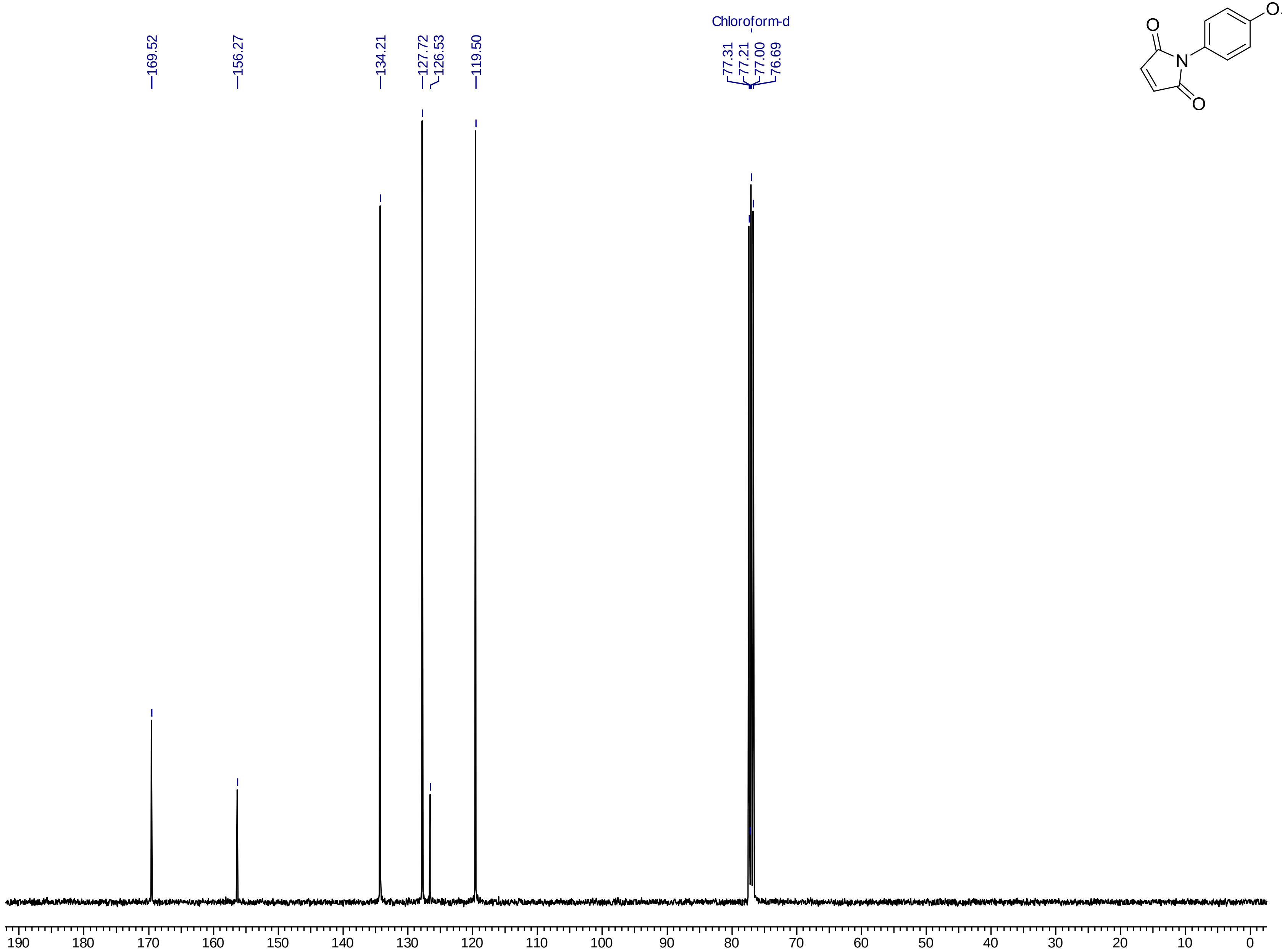

^{13}C NMR, DEPT, 100 MHz

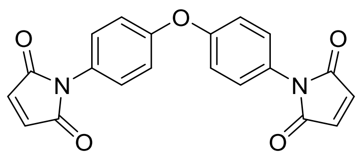

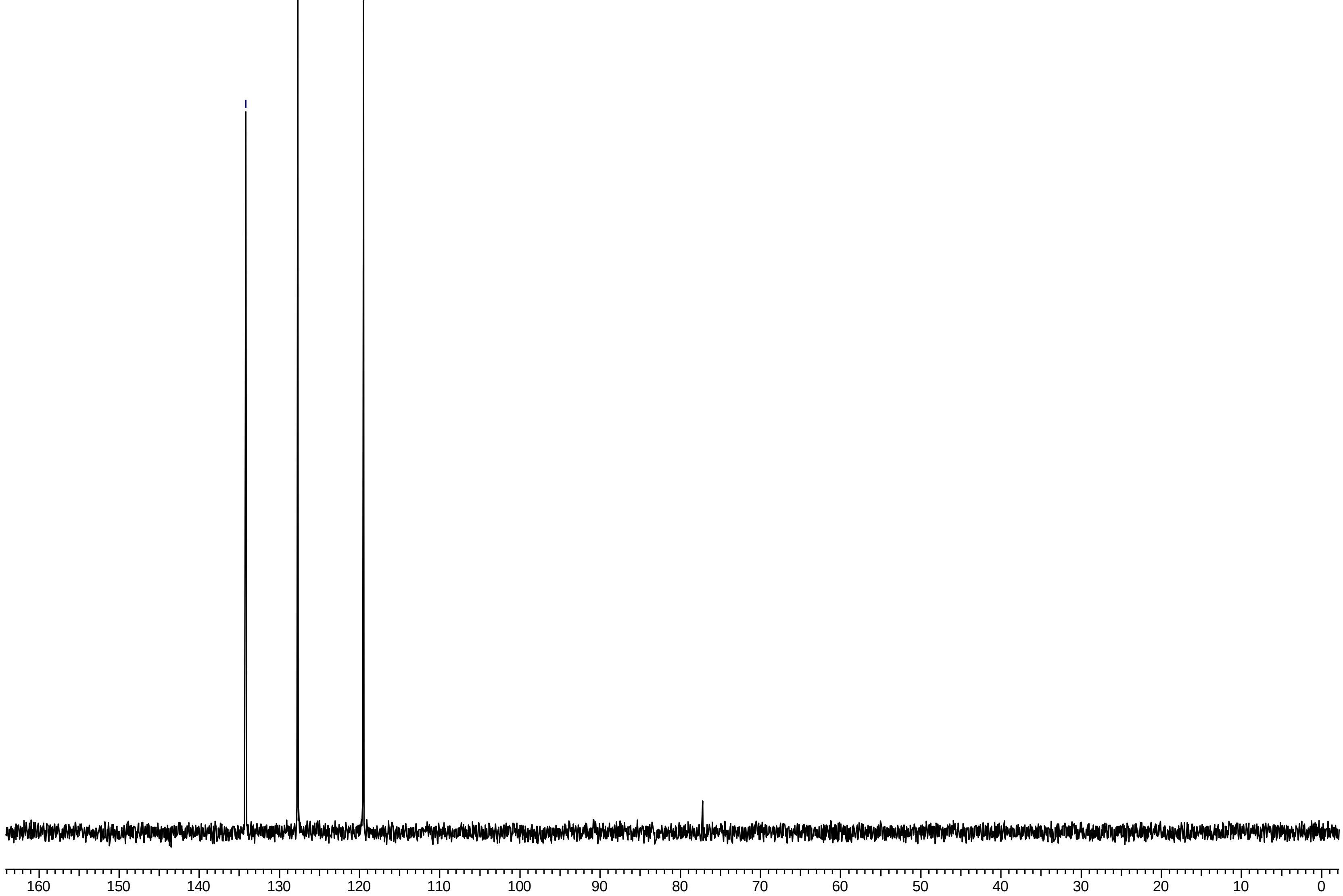

^{1}H NMR, 500 MHz

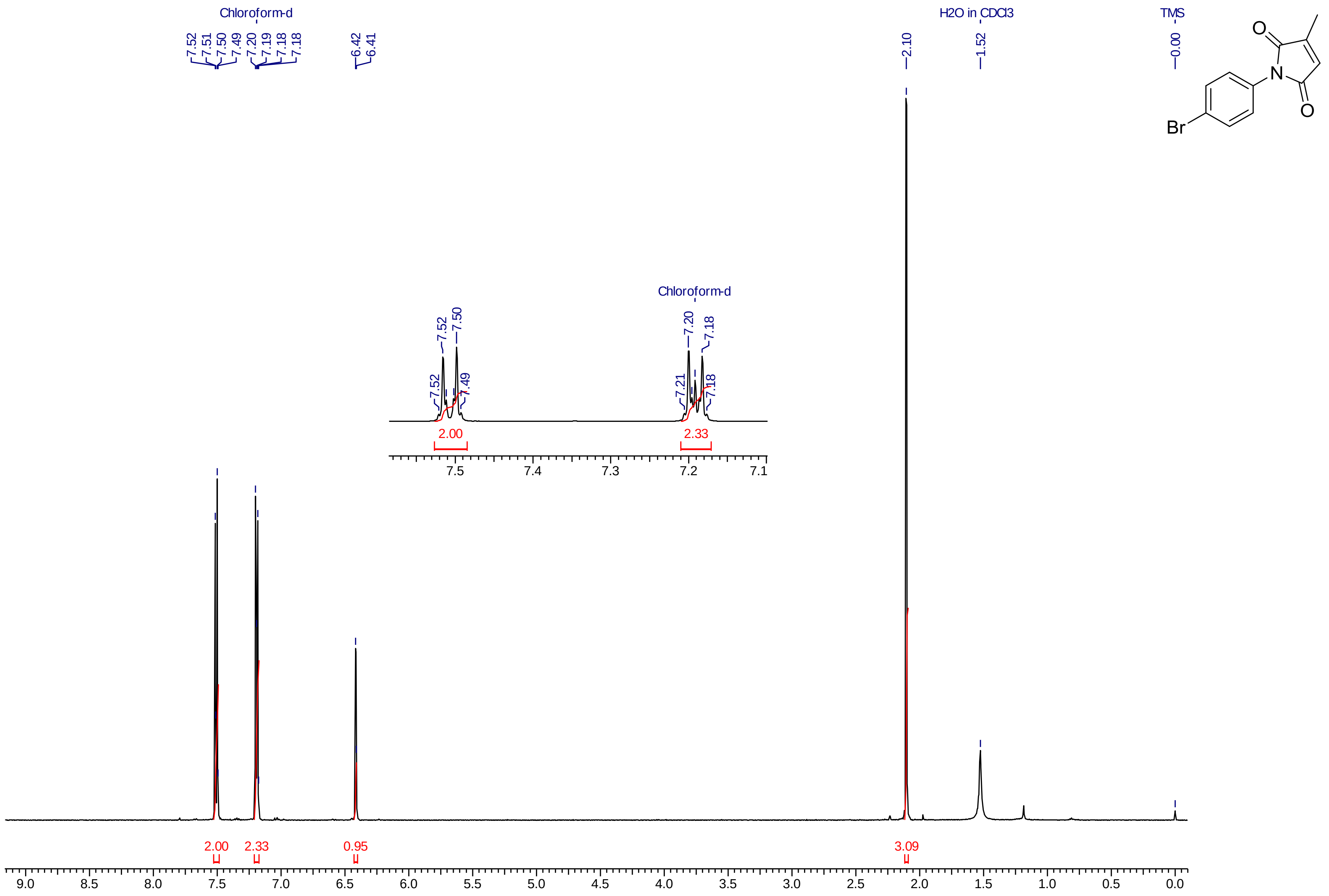

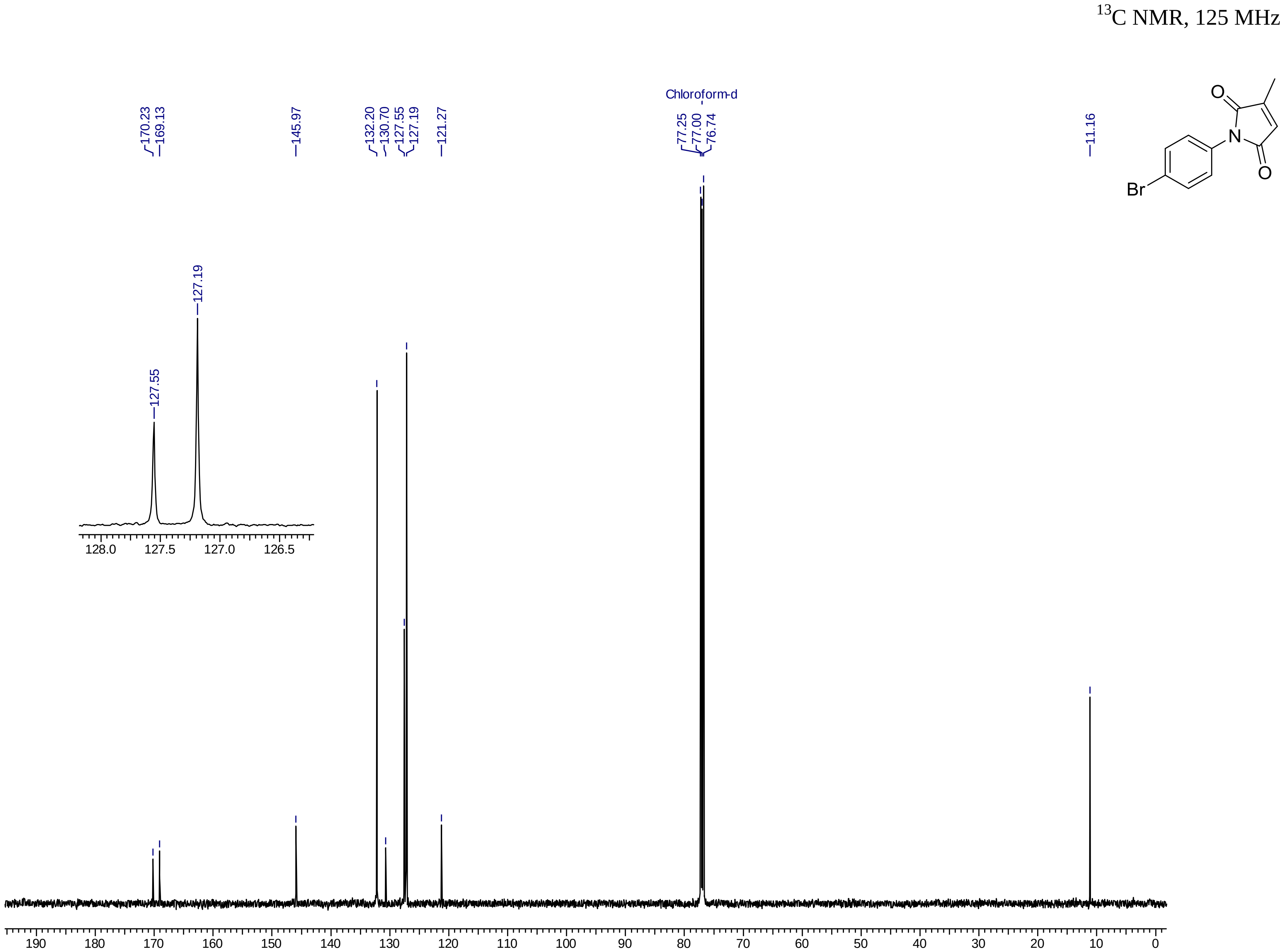

^{13}C NMR, 125 MHz
170.23
169.13
145.97
132.20
130.70
127.55
127.19
121.27
Chloroform-d
77.25
77.00
76.74
11.16
127.55
127.19
128.0
127.5
127.0
126.5
O
N
O
Br
190
180
170
160
150
140
130
120
110
100
90
80
70
60
50
40
30
20
10
0

^{13}C NMR, DEPT, 125 MHz

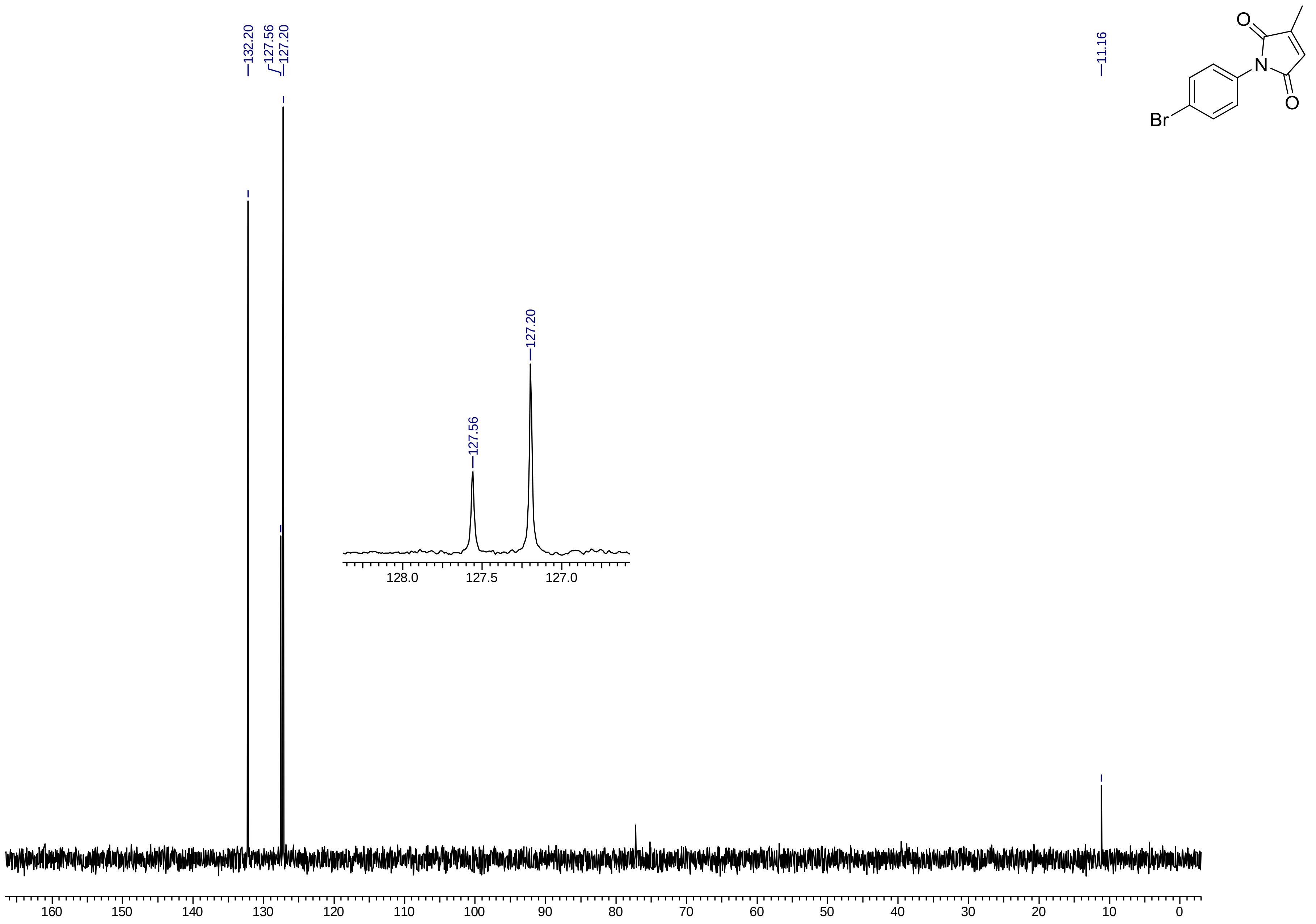

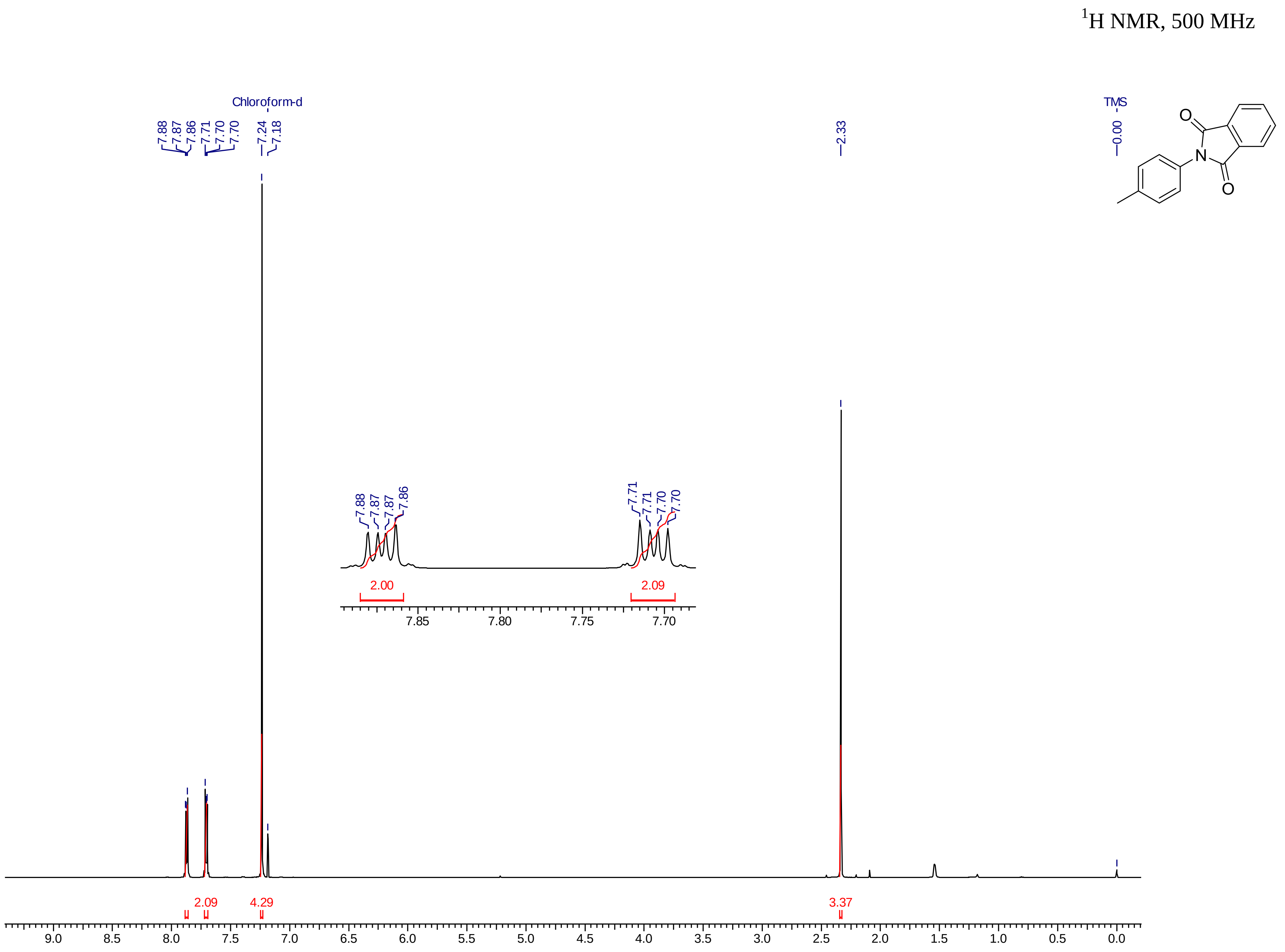

^{1}H NMR, 500 MHz
Chloroform-d
TMS
7.88
7.87
7.86
7.71
7.70
7.70
7.24
7.18
2.33
0.00
2.09
4.29
3.37
2.00
2.09
O
N
O

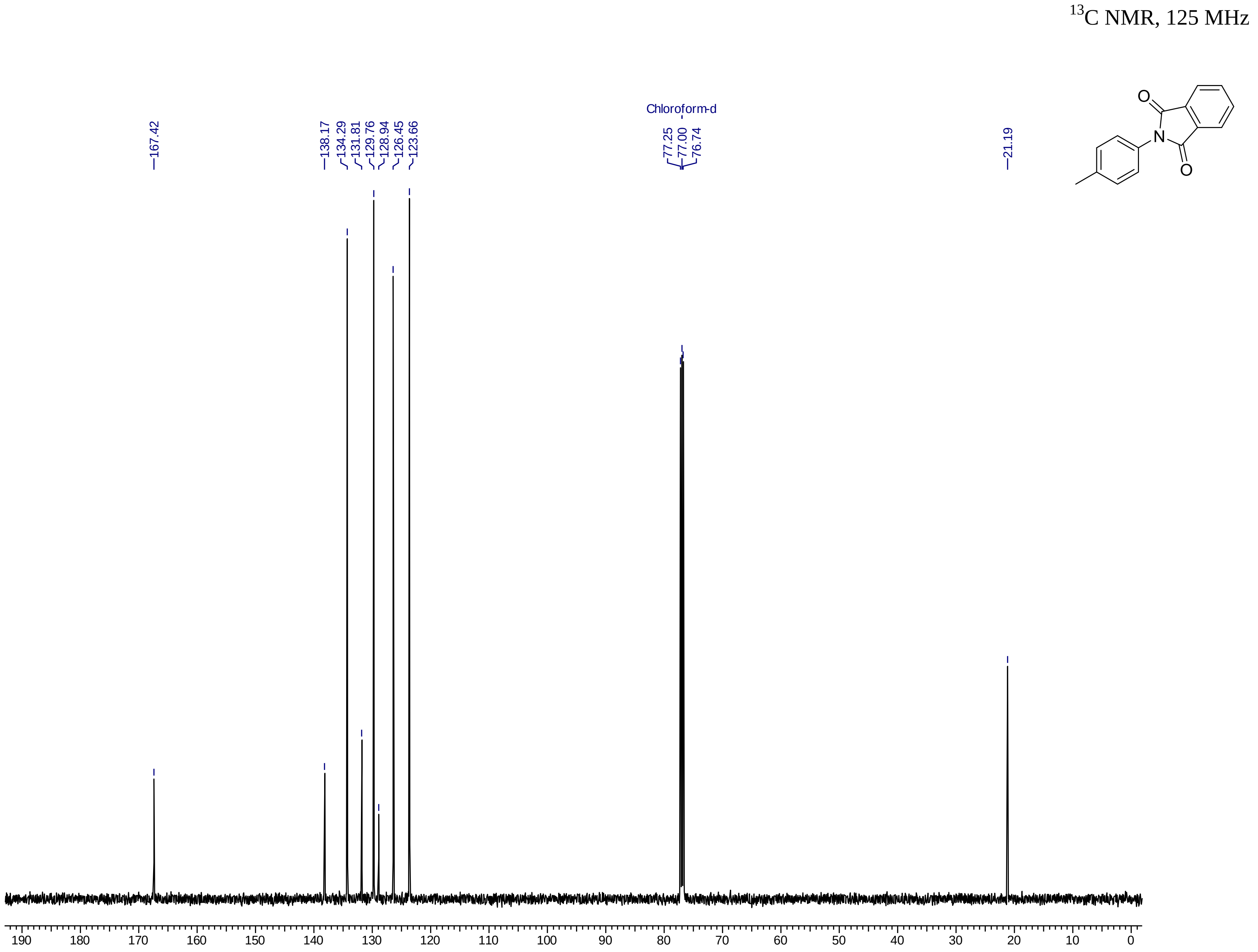
13C NMR, 125 MHz
Chloroform-d
167.42
138.17
134.29
131.81
129.76
128.94
126.45
123.66
77.25
77.00
76.74
21.19
190
180
170
160
150
140
130
120
110
100
90
80
70
60
50
40
30
20
10
0

^{13}C NMR, DEPT, 125 MHz

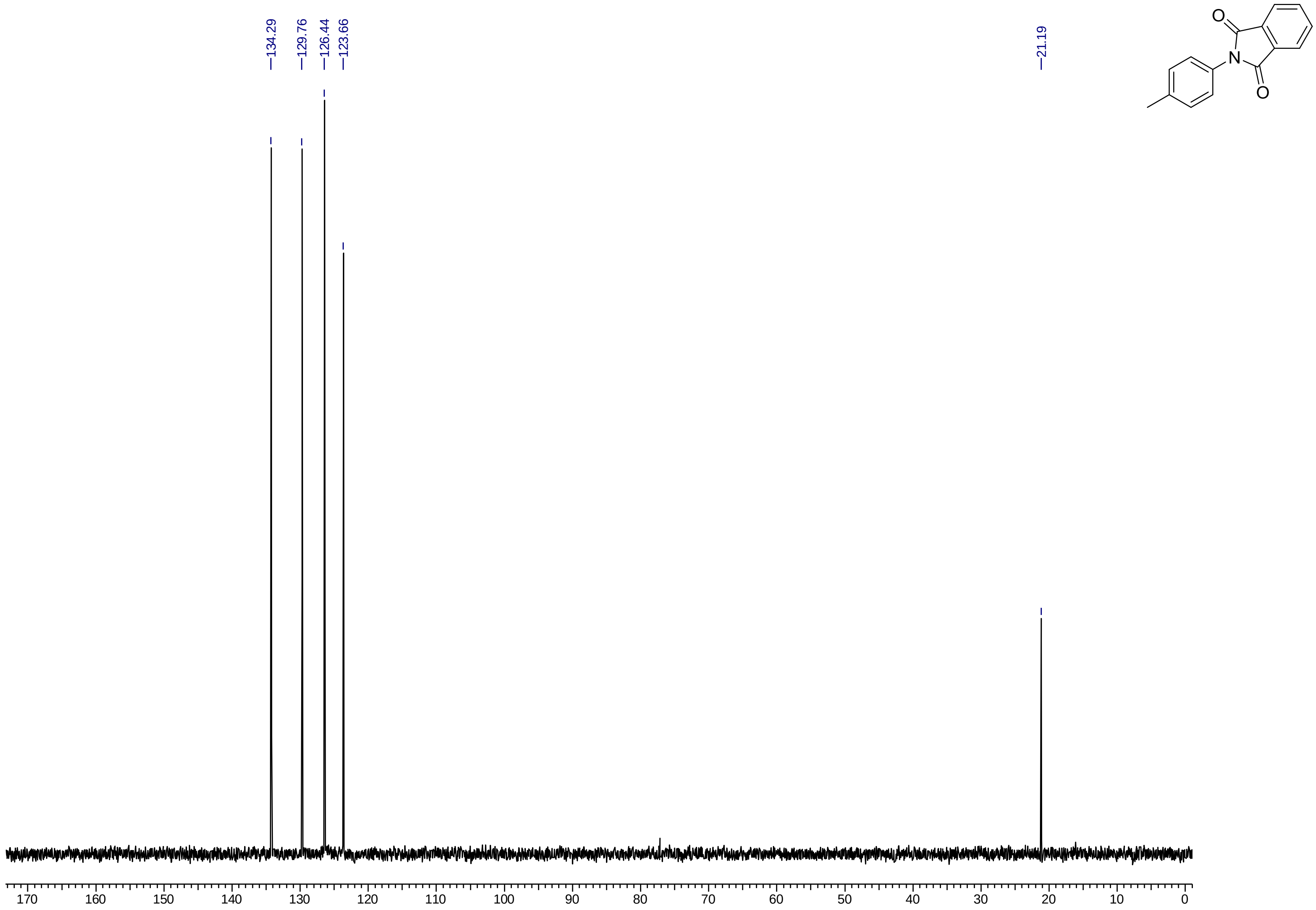

^{1}H NMR, 400 MHz

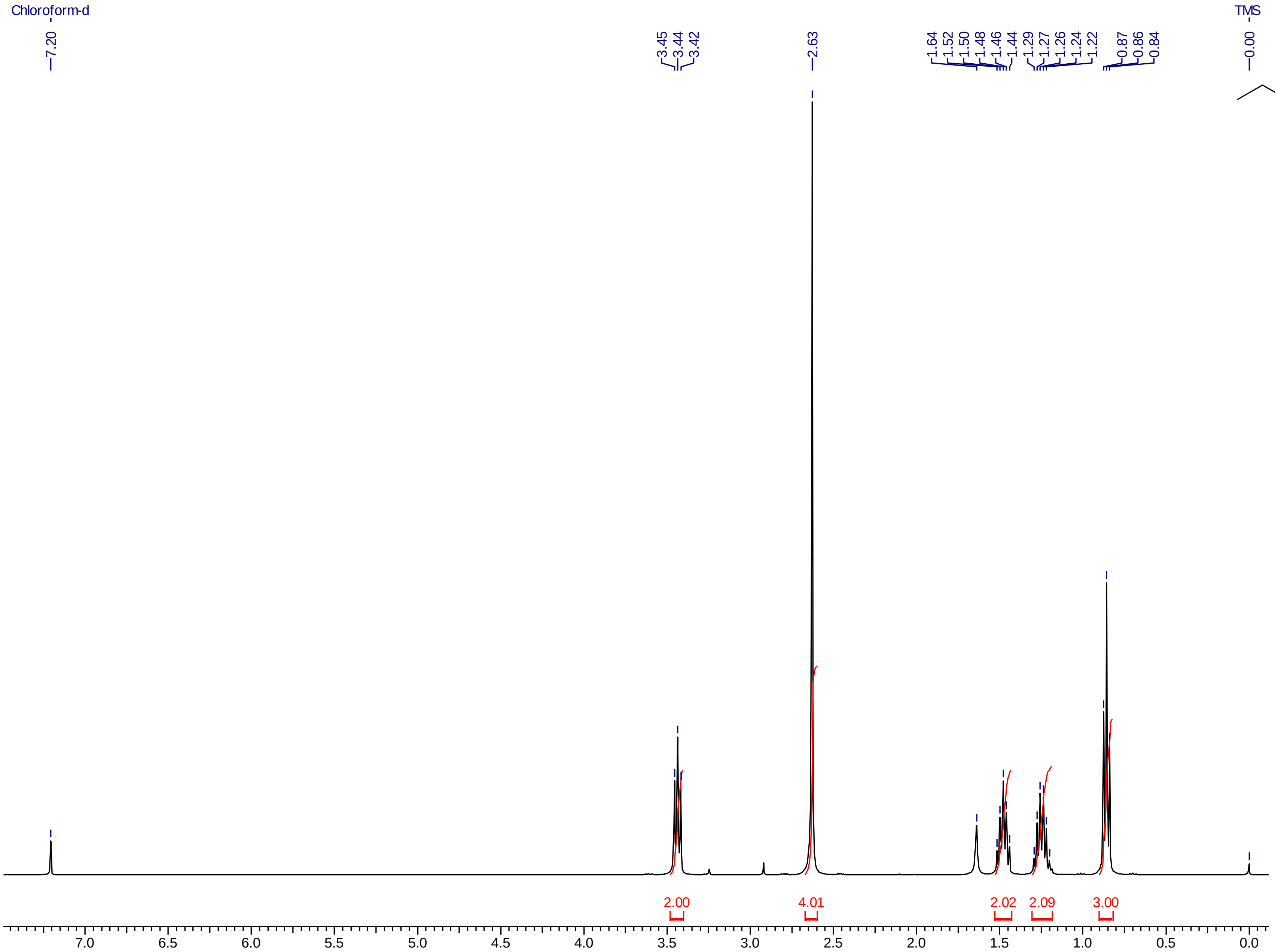

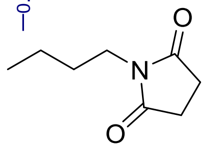

^{13}C NMR, 100 MHz

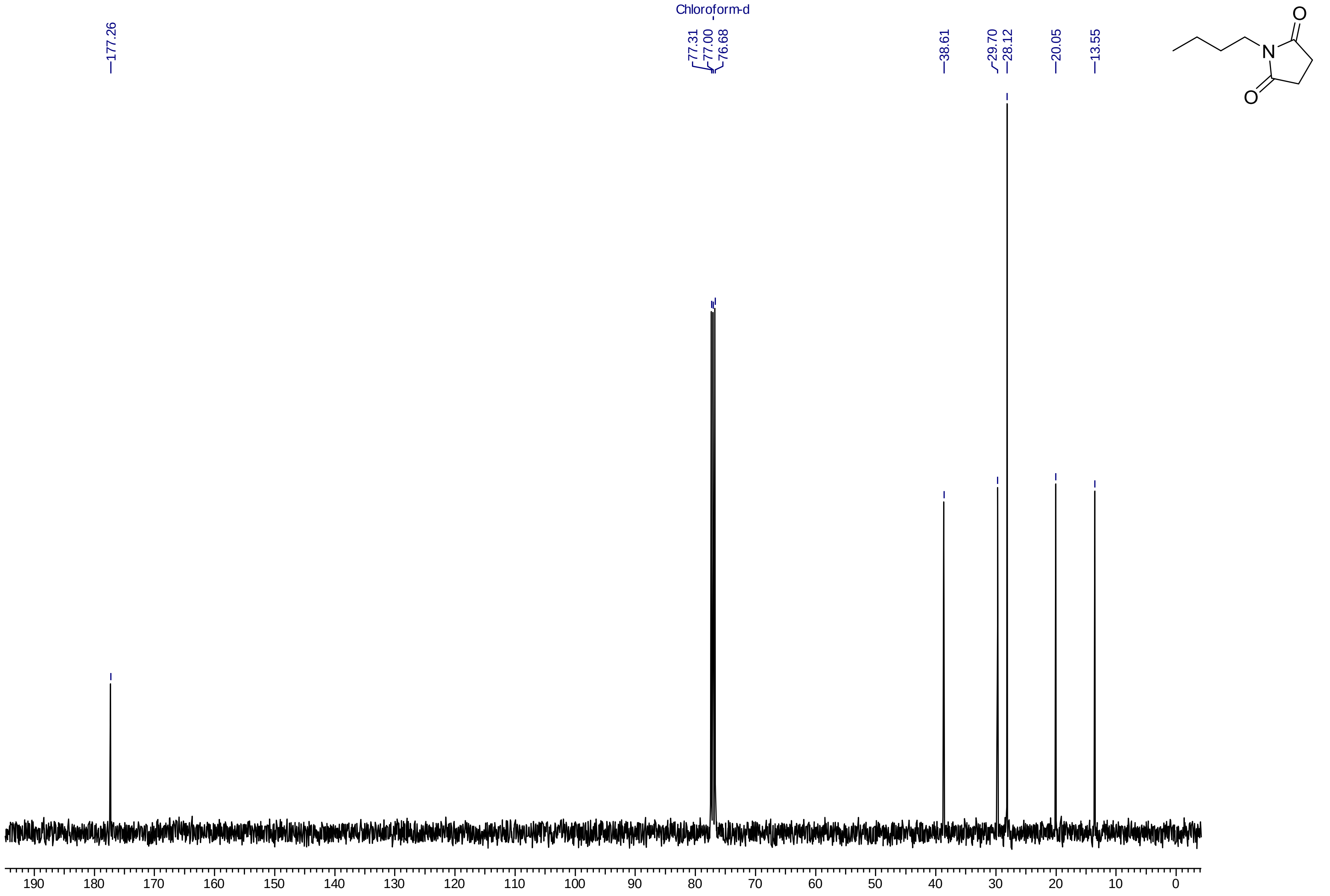

^{13}C NMR, DEPT, 100 MHz

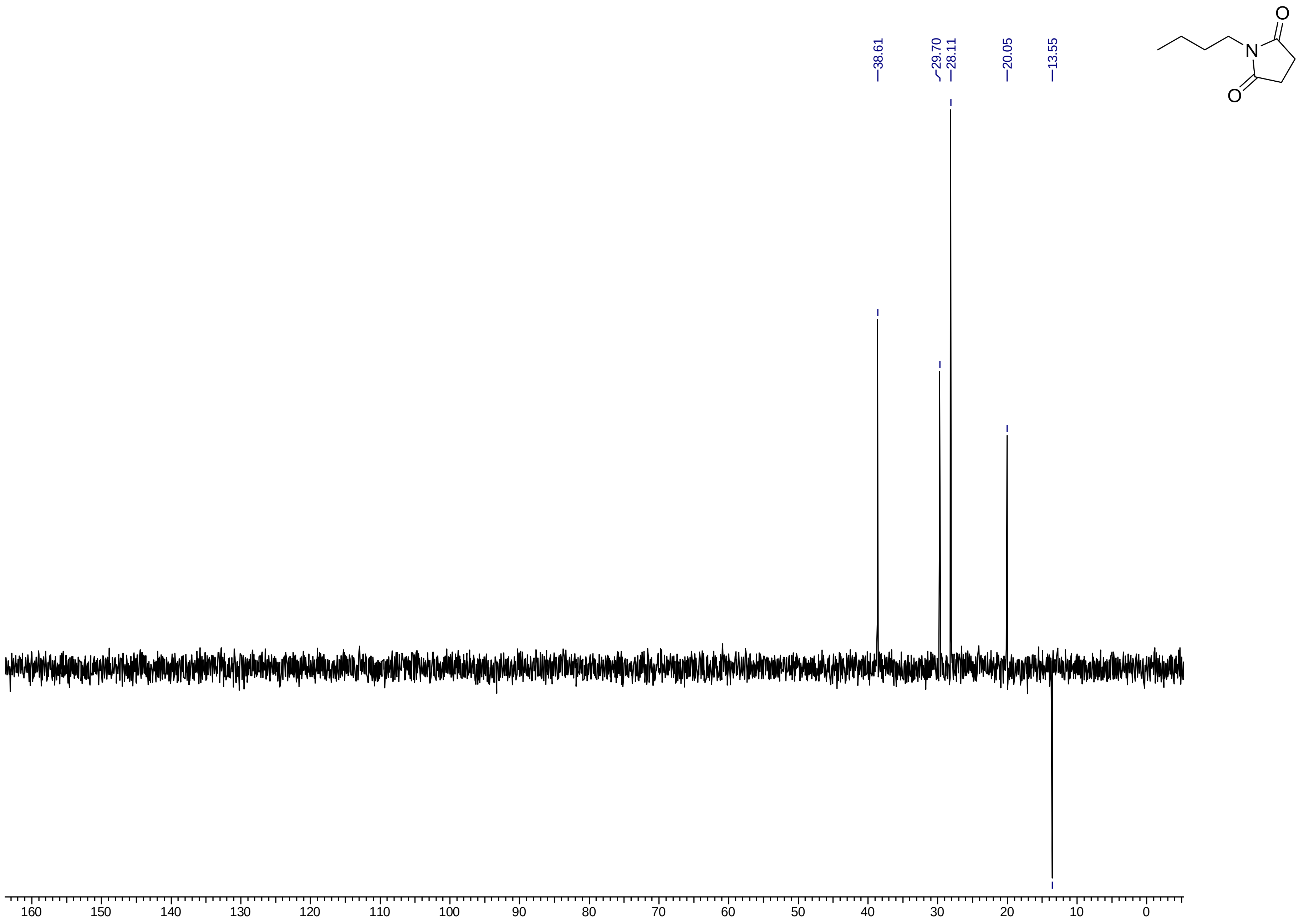

^{1}H NMR, 500 MHz

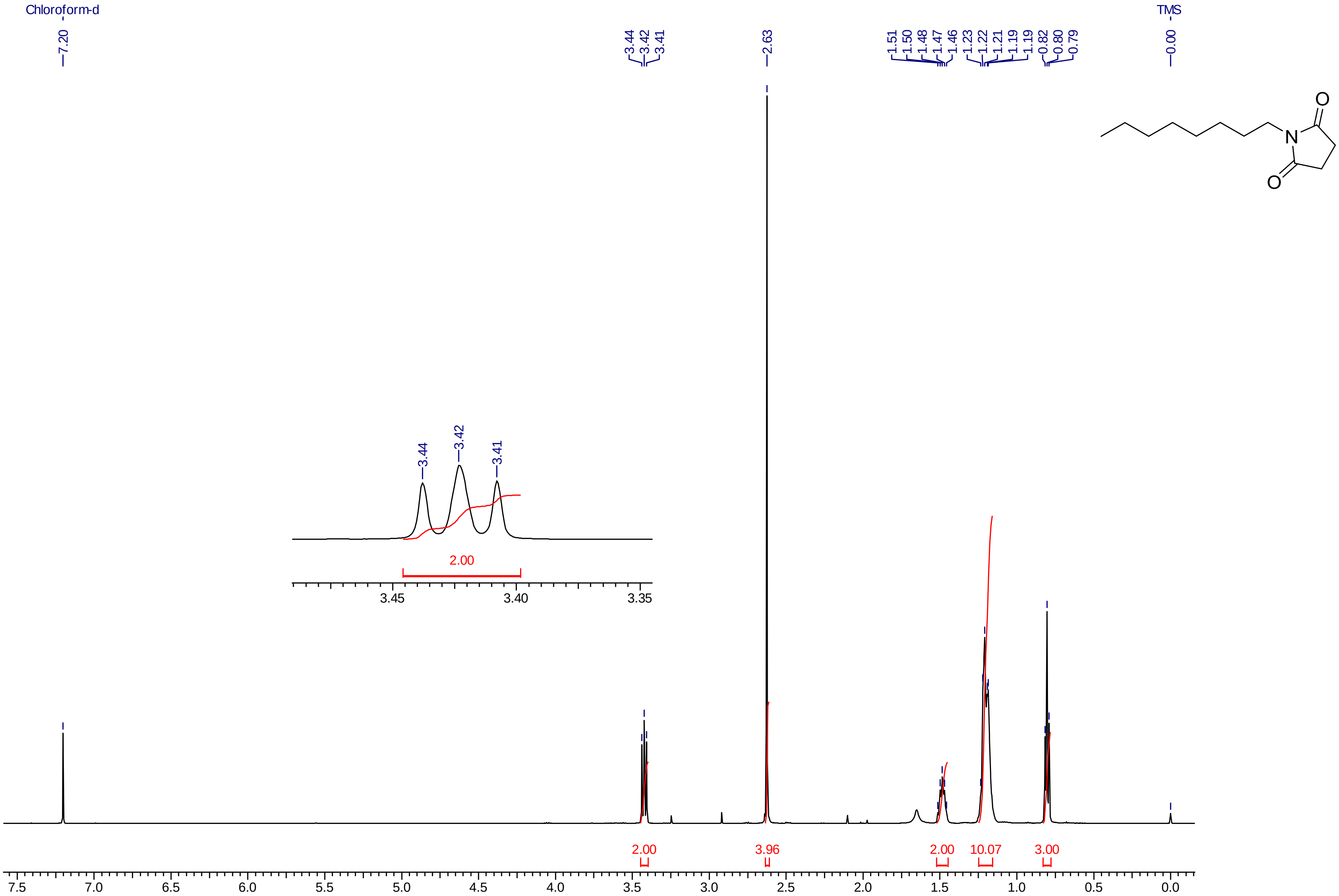

^{13}C NMR, 125 MHz

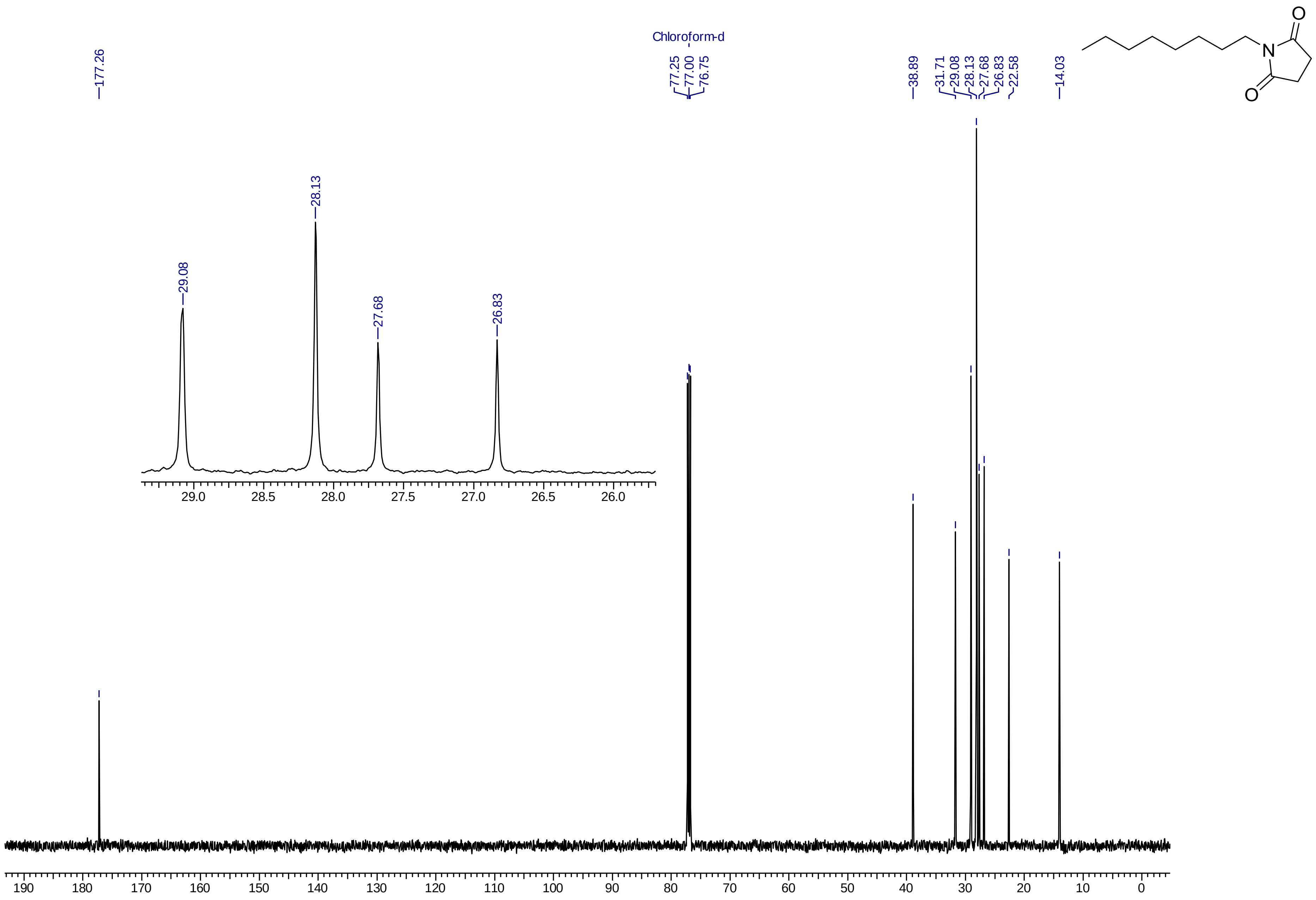

^{13}C NMR, DEPT, 125 MHz

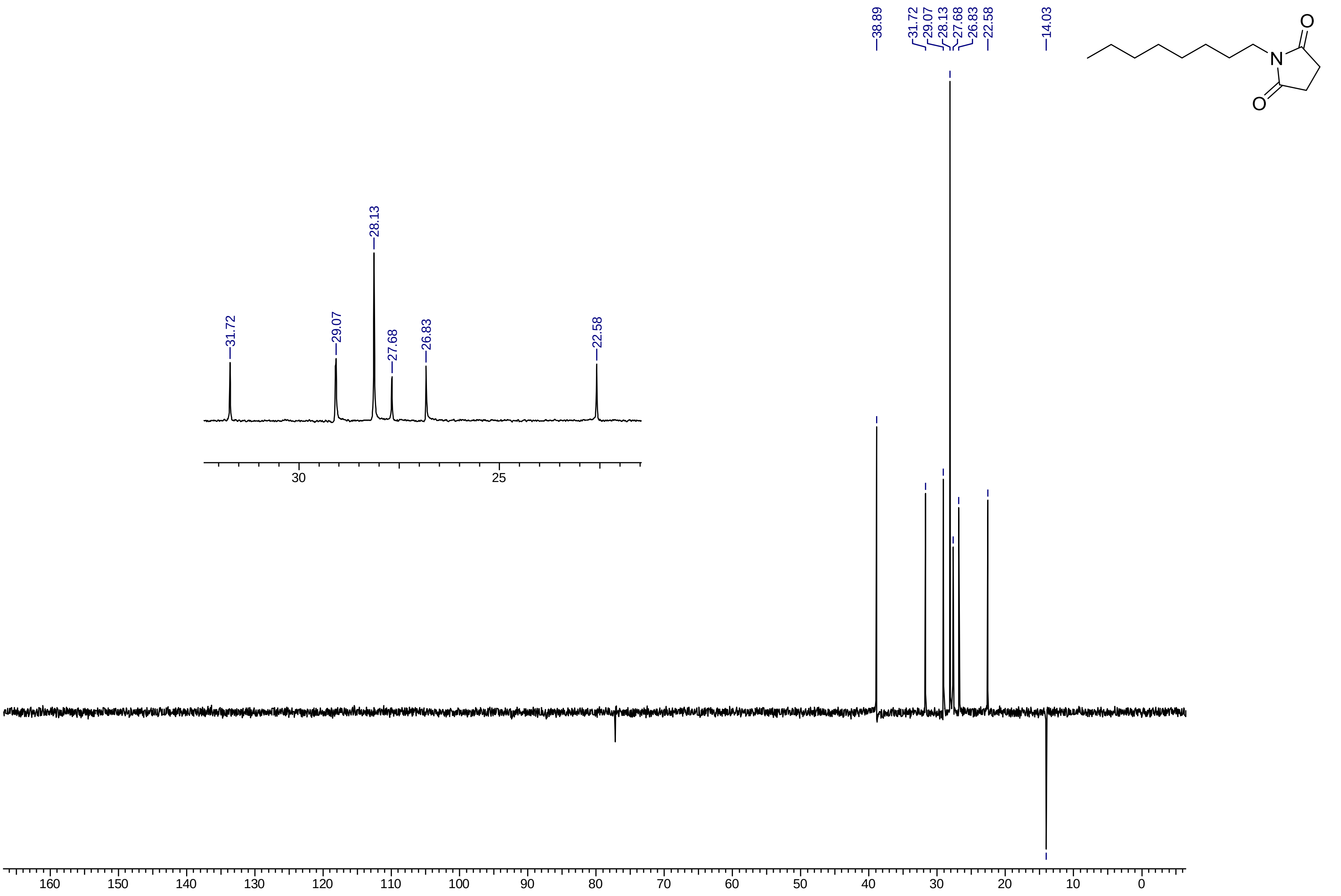

^{1}H NMR, 400 MHz

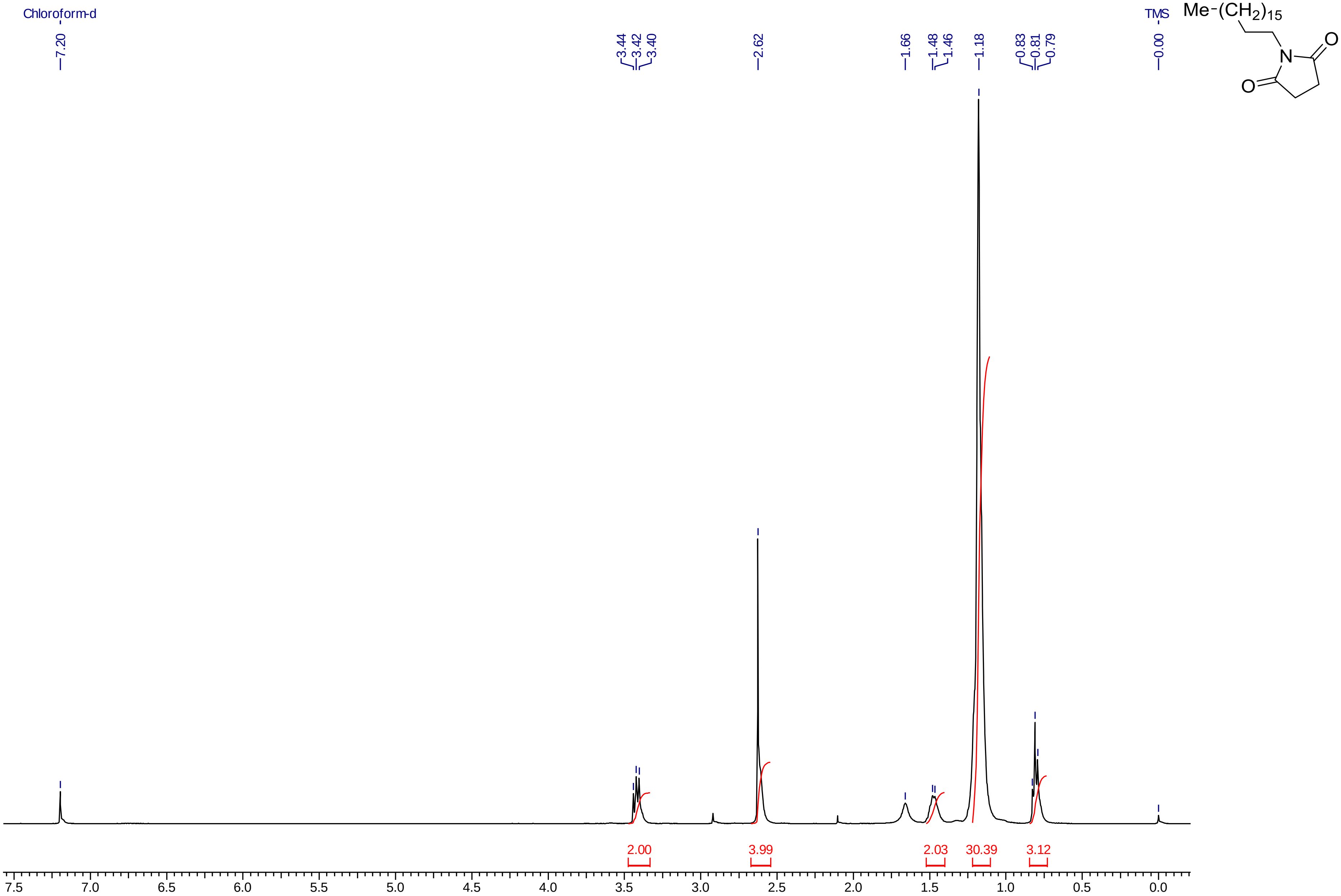

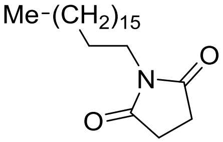
Me-(CH2)15
N
O
O

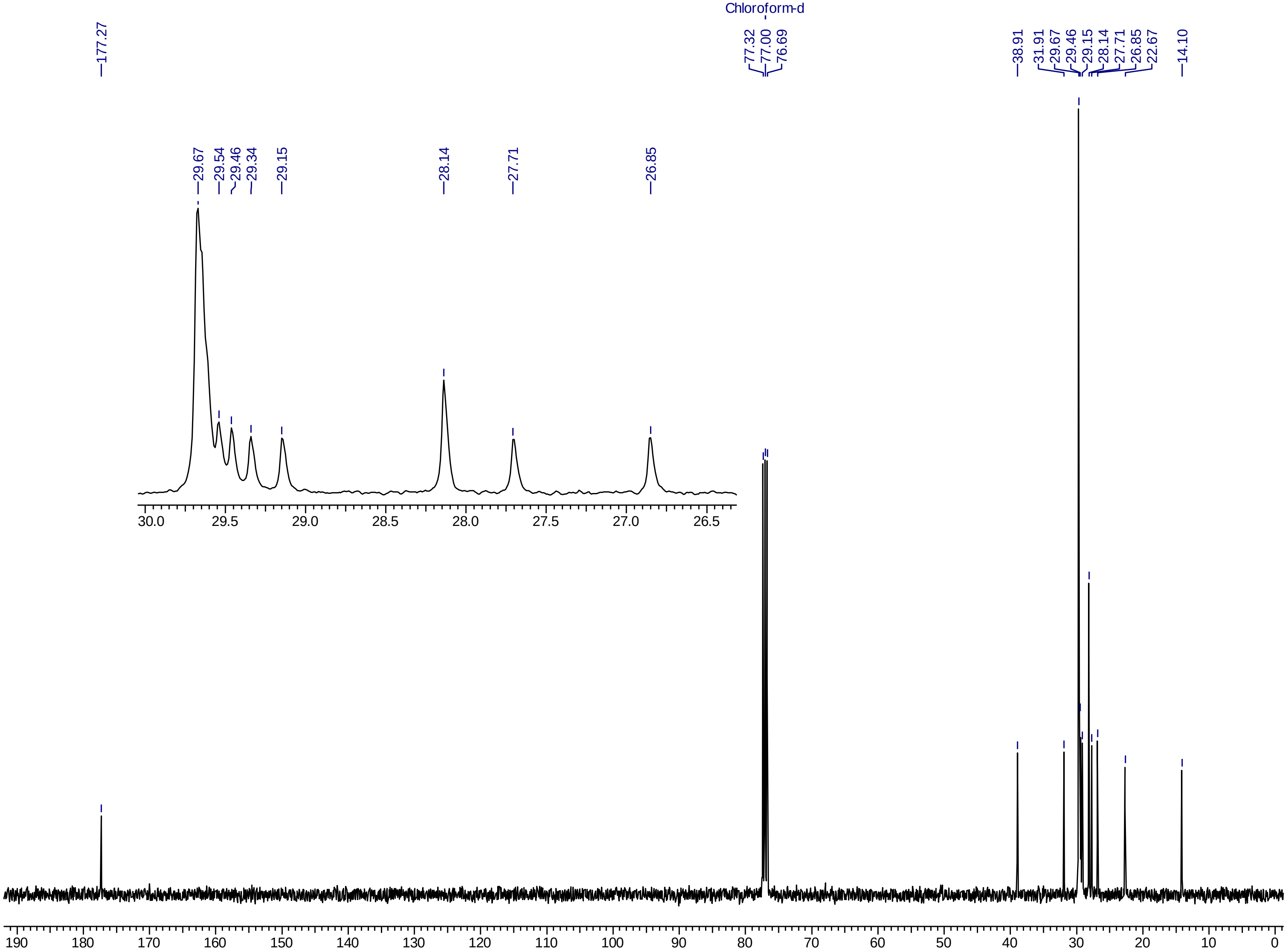
177.27
Chloroform-d
77.32
77.00
76.69
38.91
31.91
29.67
29.46
29.15
28.14
27.71
26.85
22.67
14.10
29.67
29.54
29.46
29.34
29.15
28.14
27.71
26.85
30.0 29.5 29.0 28.5 28.0 27.5 27.0 26.5
190 180 170 160 150 140 130 120 110 100 90 80 70 60 50 40 30 20 10 0

^{13}C NMR, DEPT, 100 MHz

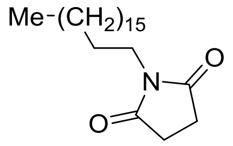

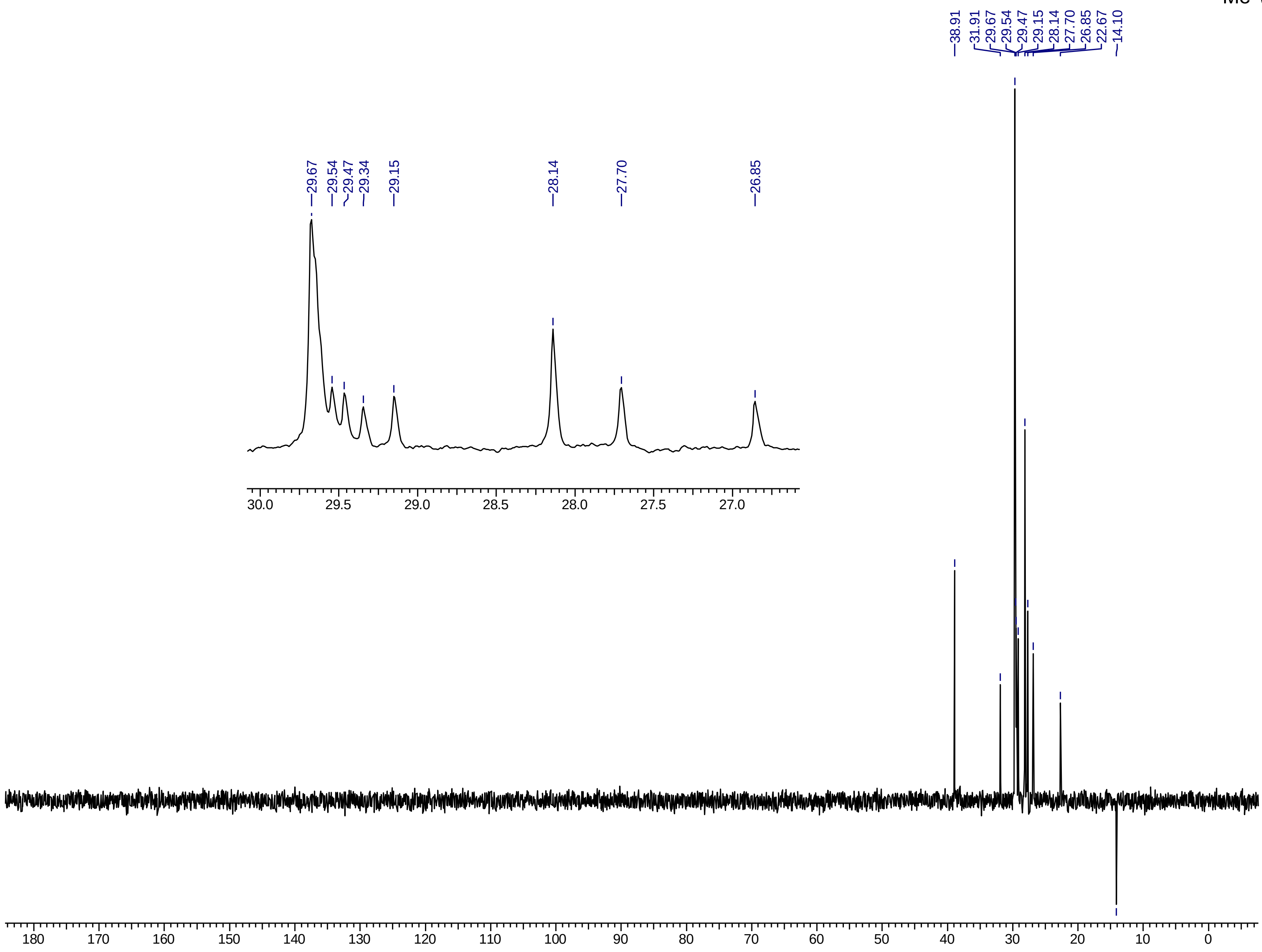

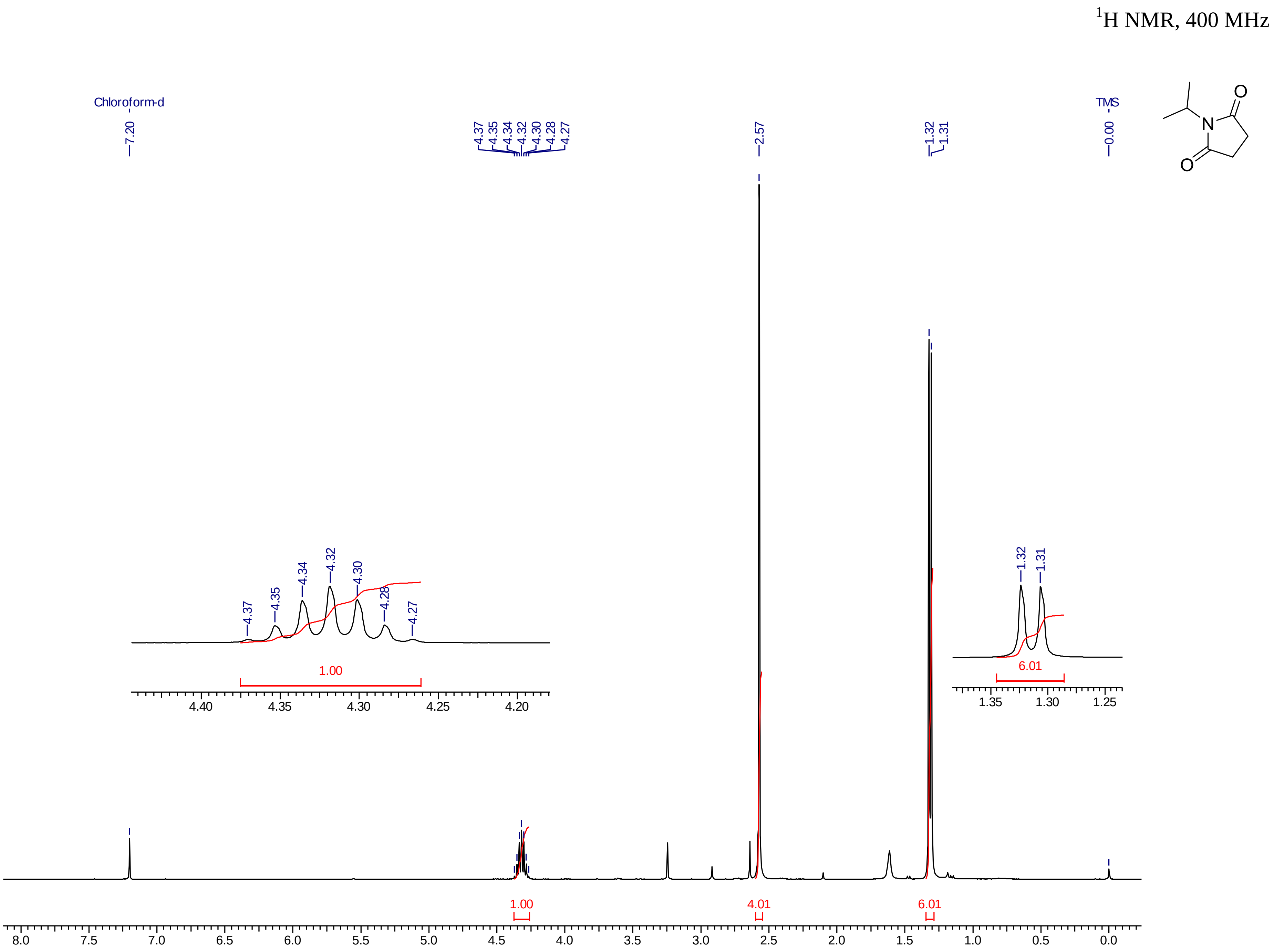

1H NMR, 400 MHz
Chloroform-d
7.20
4.37
4.35
4.34
4.32
4.30
4.28
4.27
2.57
1.32
1.31
TMS
0.00
O
N
O
1.00
4.01
6.01
8.0
7.5
7.0
6.5
6.0
5.5
5.0
4.5
4.0
3.5
3.0
2.5
2.0
1.5
1.0
0.5
0.0
4.40
4.35
4.30
4.25
4.20
1.35
1.30
1.25

^{13}C NMR, 100 MHz

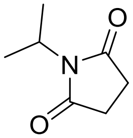

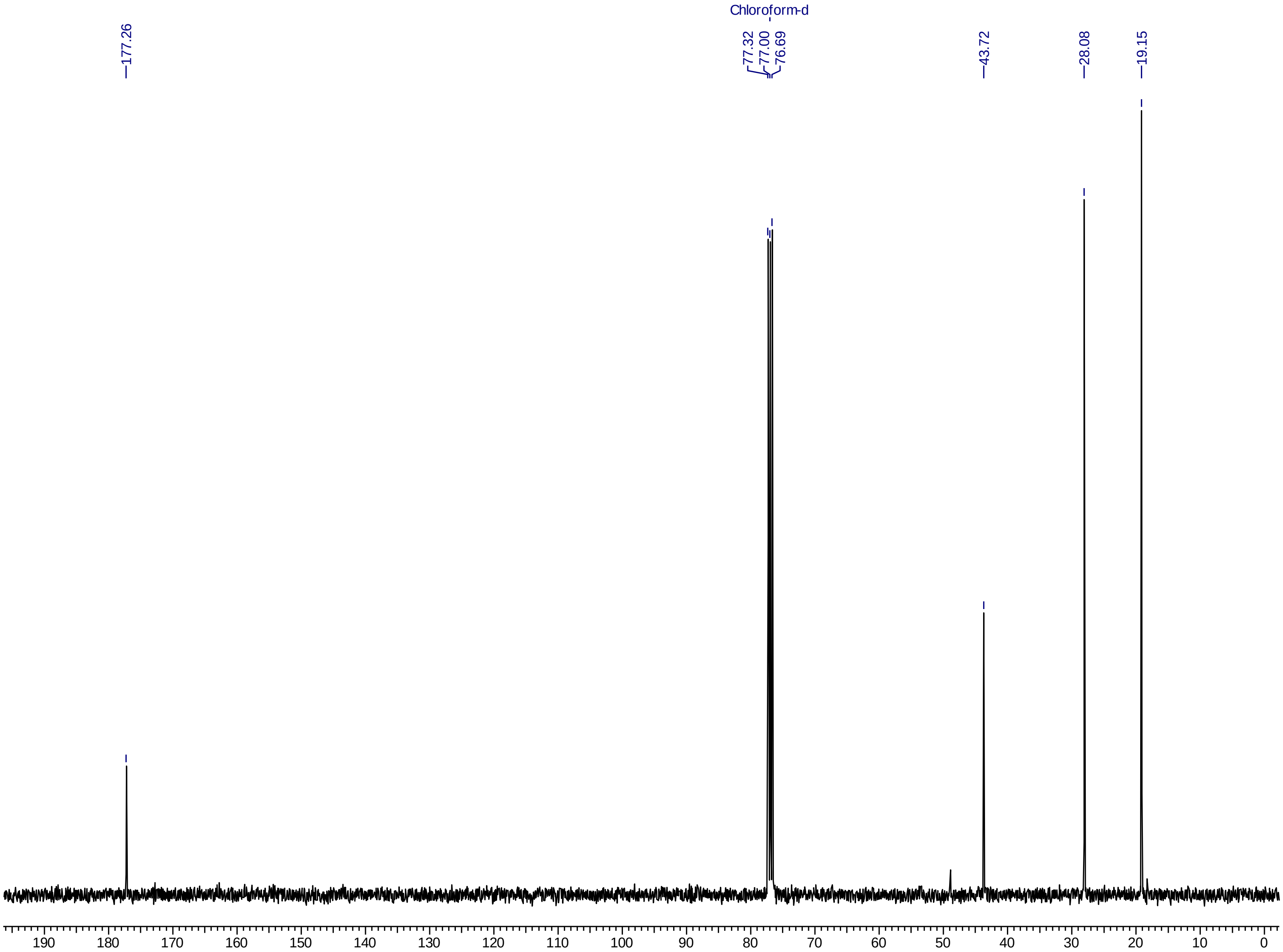

^{13}C NMR. DEPT. 100 MHz

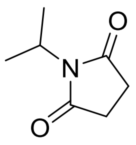

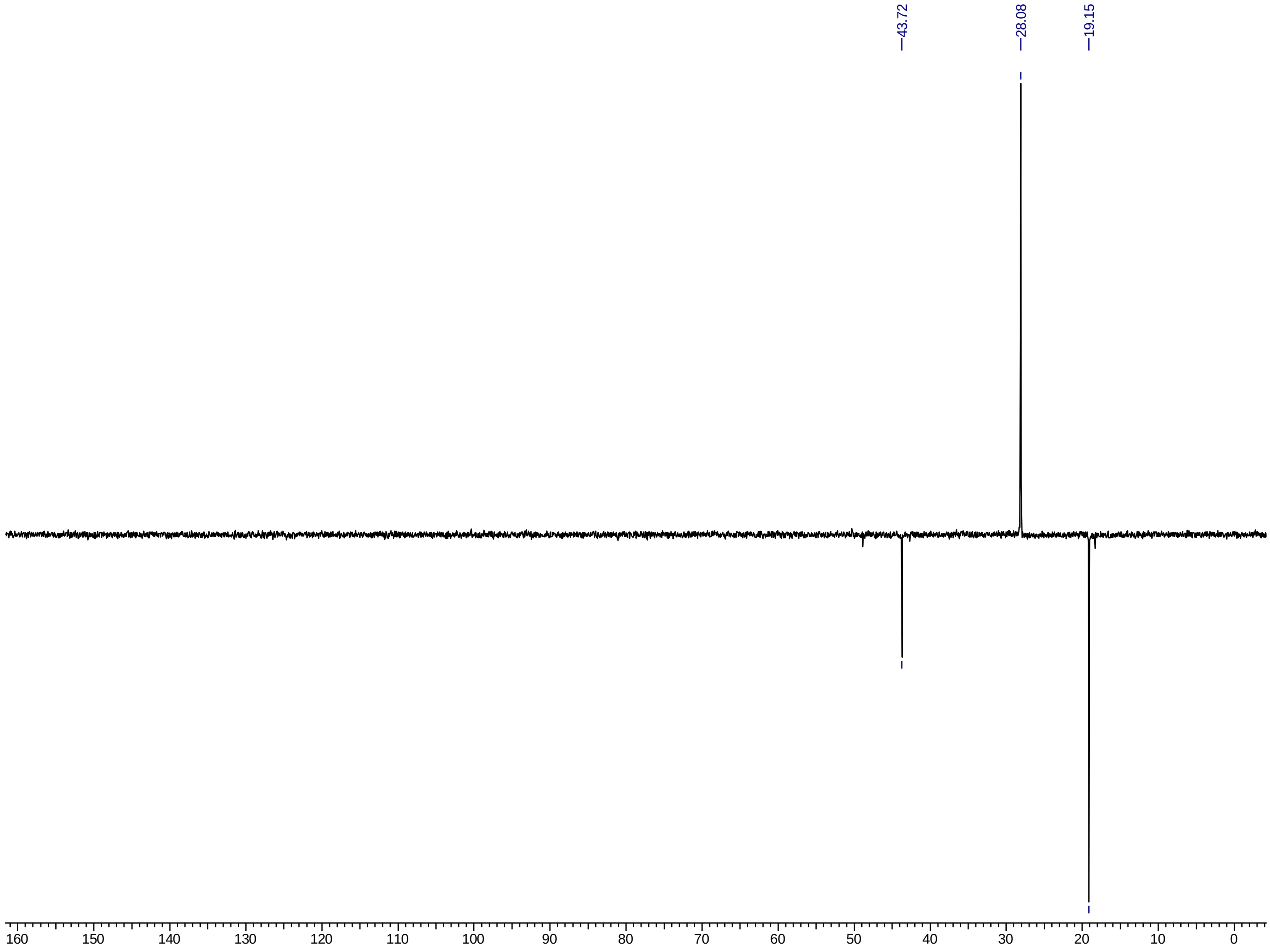

^{1}H NMR, 500 MHz

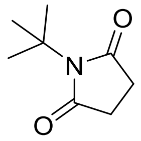

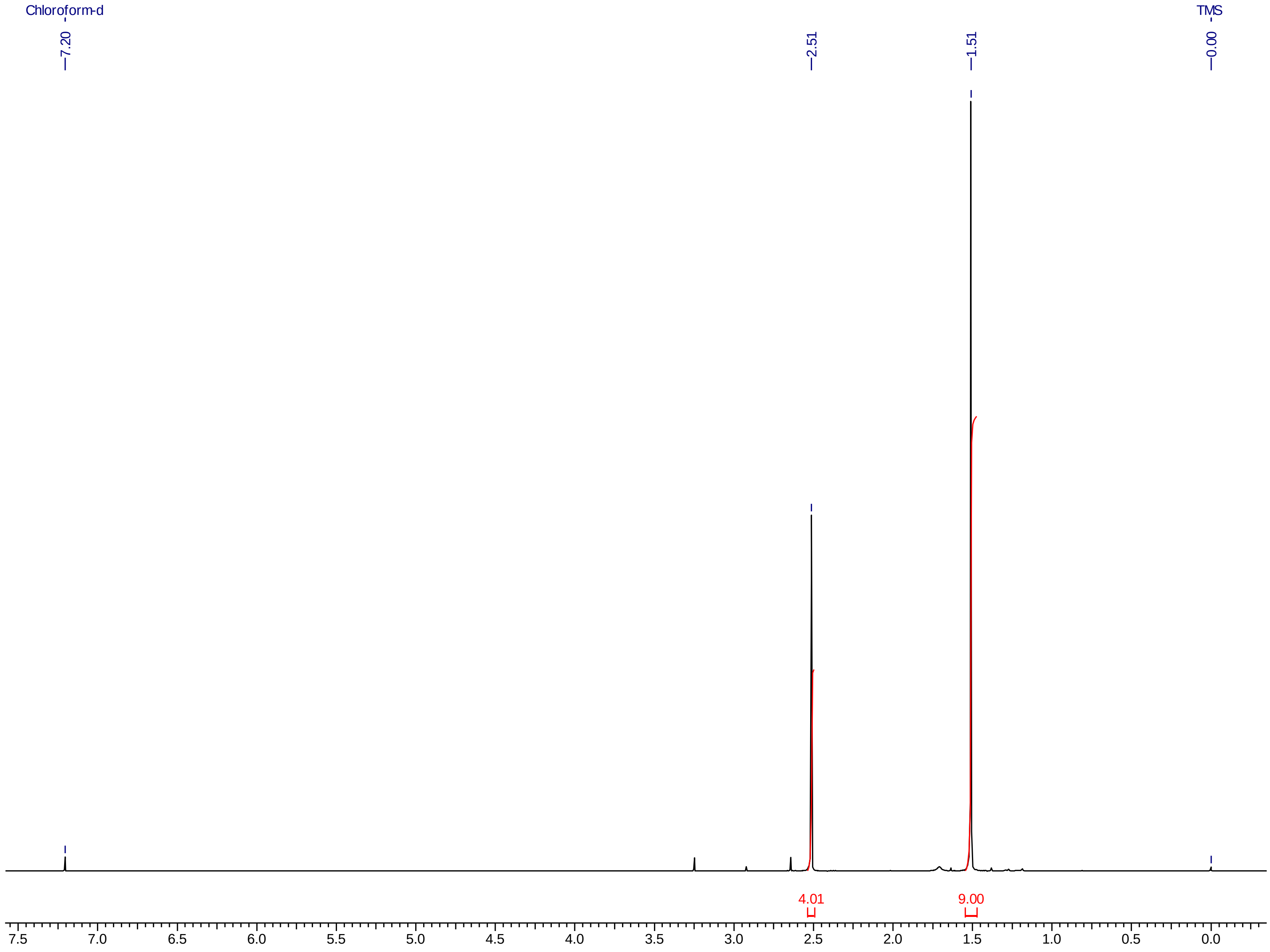

^{13}C NMR, 125 MHz

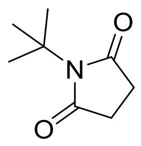

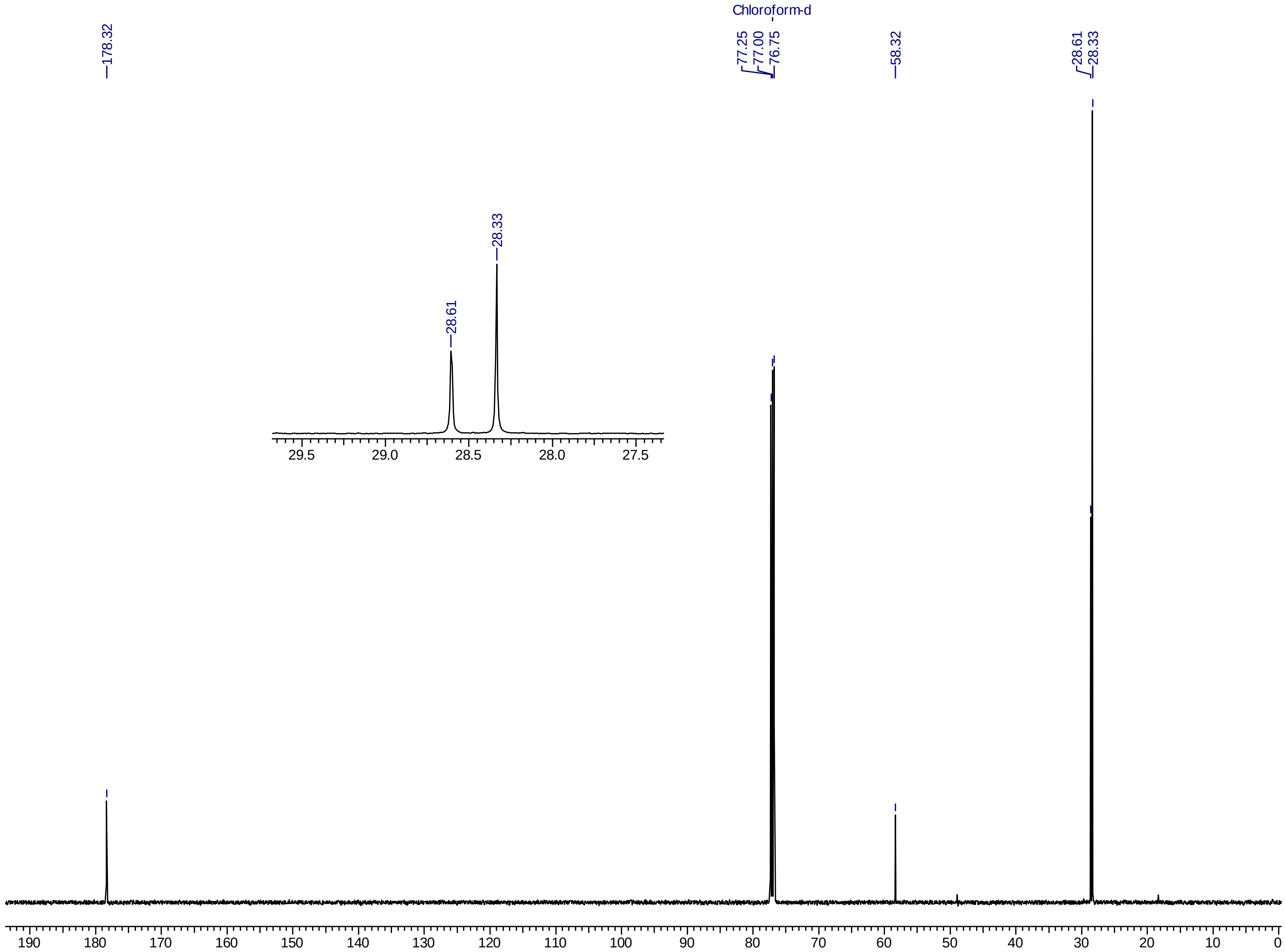

^{13}C NMR, DEPT, 125 MHz

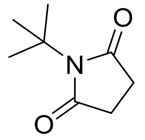

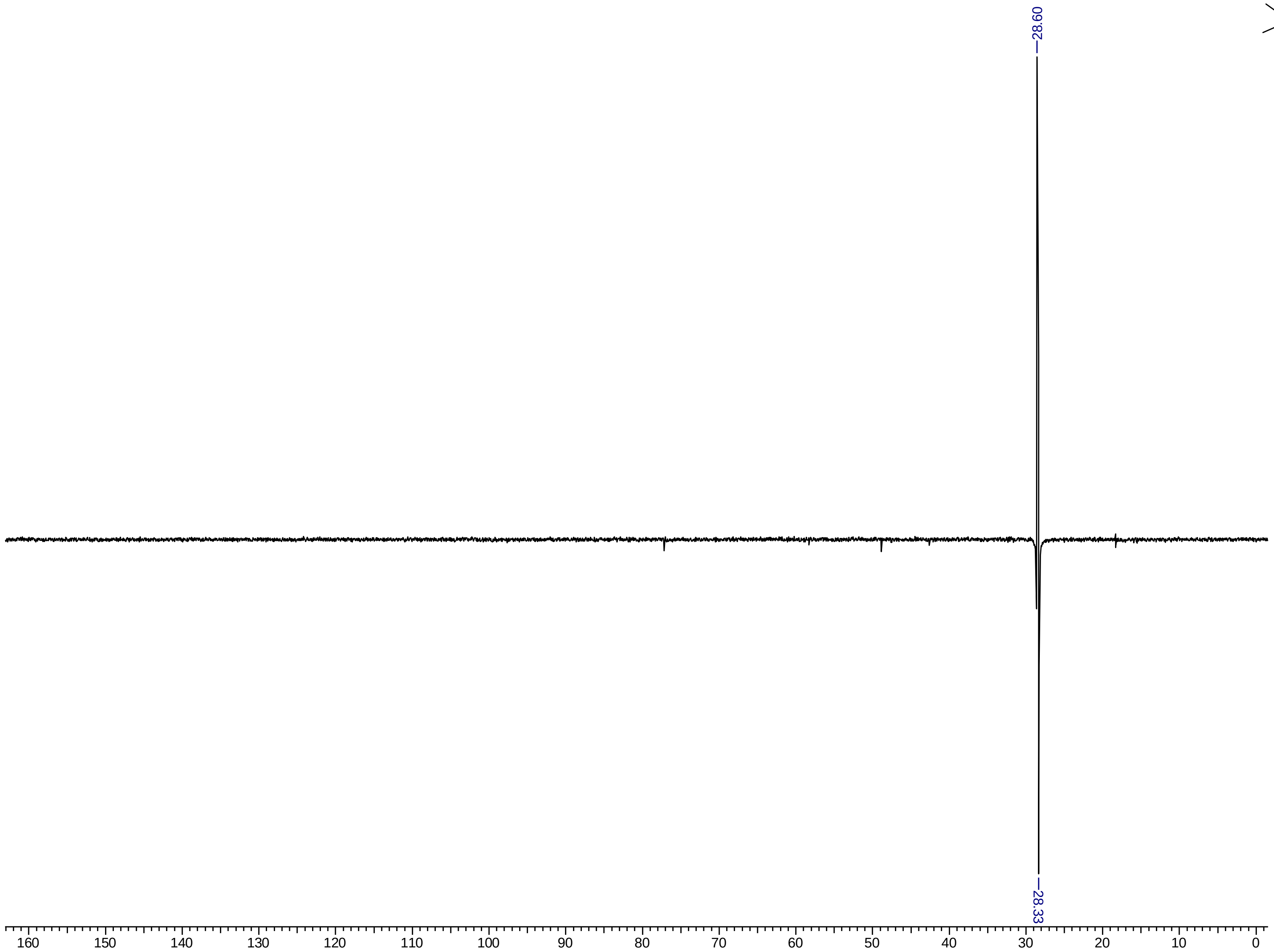

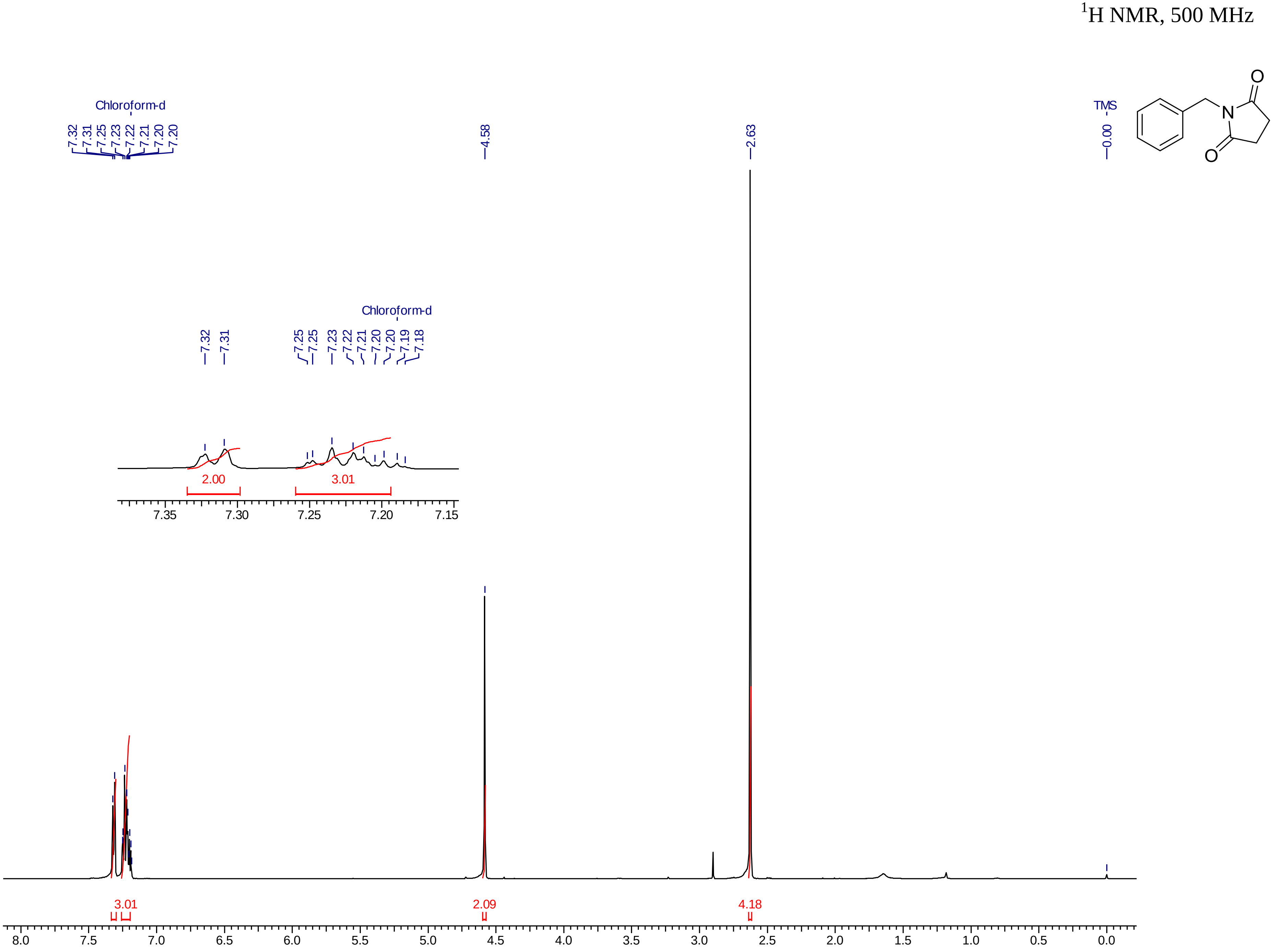
1H NMR, 500 MHz
Chloroform-d
7.32
7.31
7.25
7.23
7.22
7.21
7.20
7.20
4.58
2.63
TMS
0.00
N
O
O
Chloroform-d
7.32
7.31
7.25
7.25
7.23
7.22
7.21
7.20
7.20
7.19
7.18
2.00
3.01
7.35
7.30
7.25
7.20
7.15
3.01
2.09
4.18
8.0
7.5
7.0
6.5
6.0
5.5
5.0
4.5
4.0
3.5
3.0
2.5
2.0
1.5
1.0
0.5
0.0

^{13}C NMR, 125 MHz

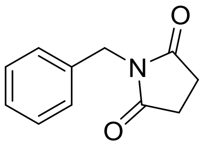

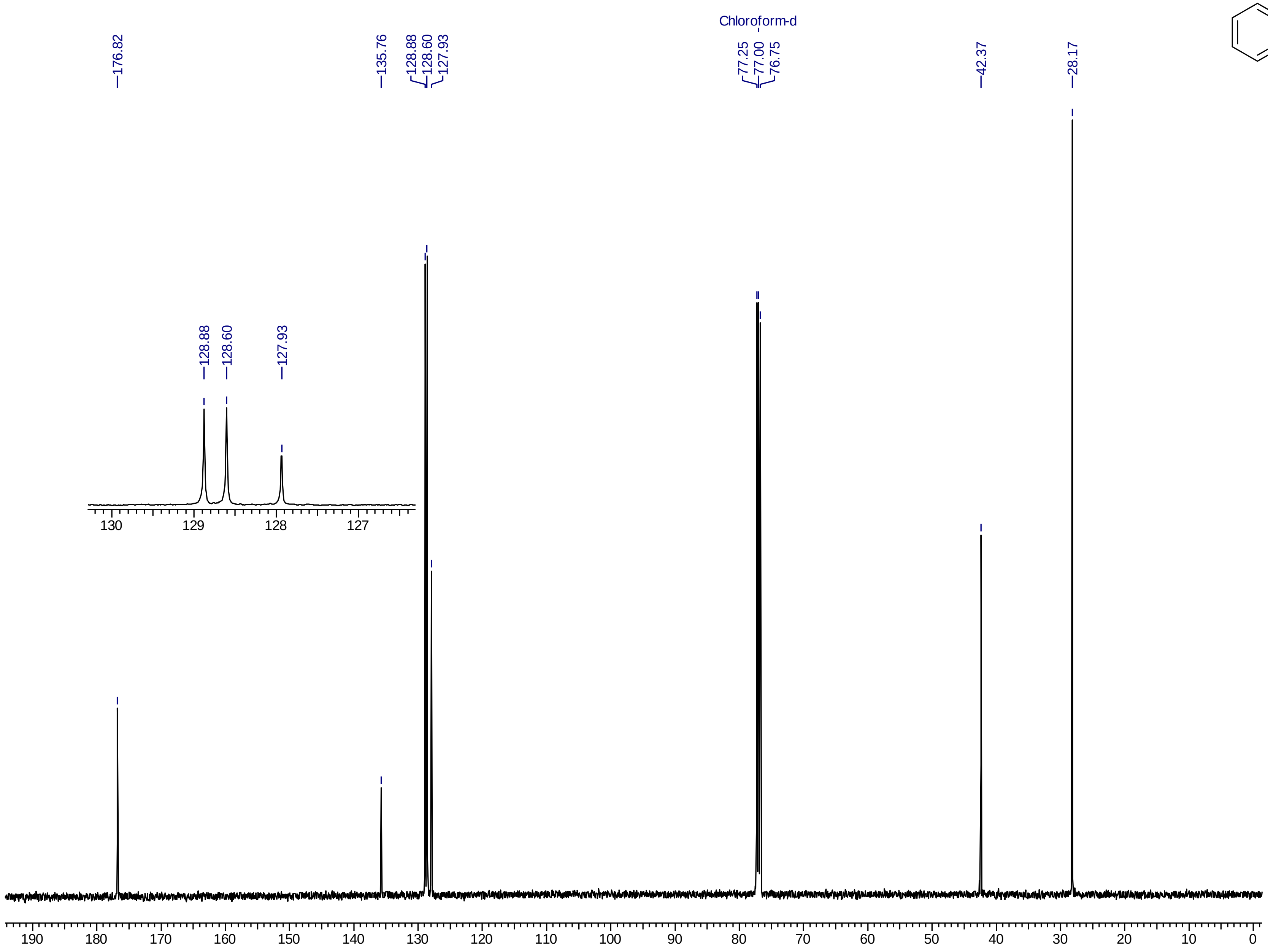

^{13}C NMR, DEPT, 125 MHz

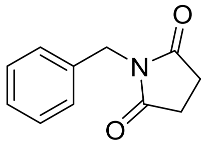

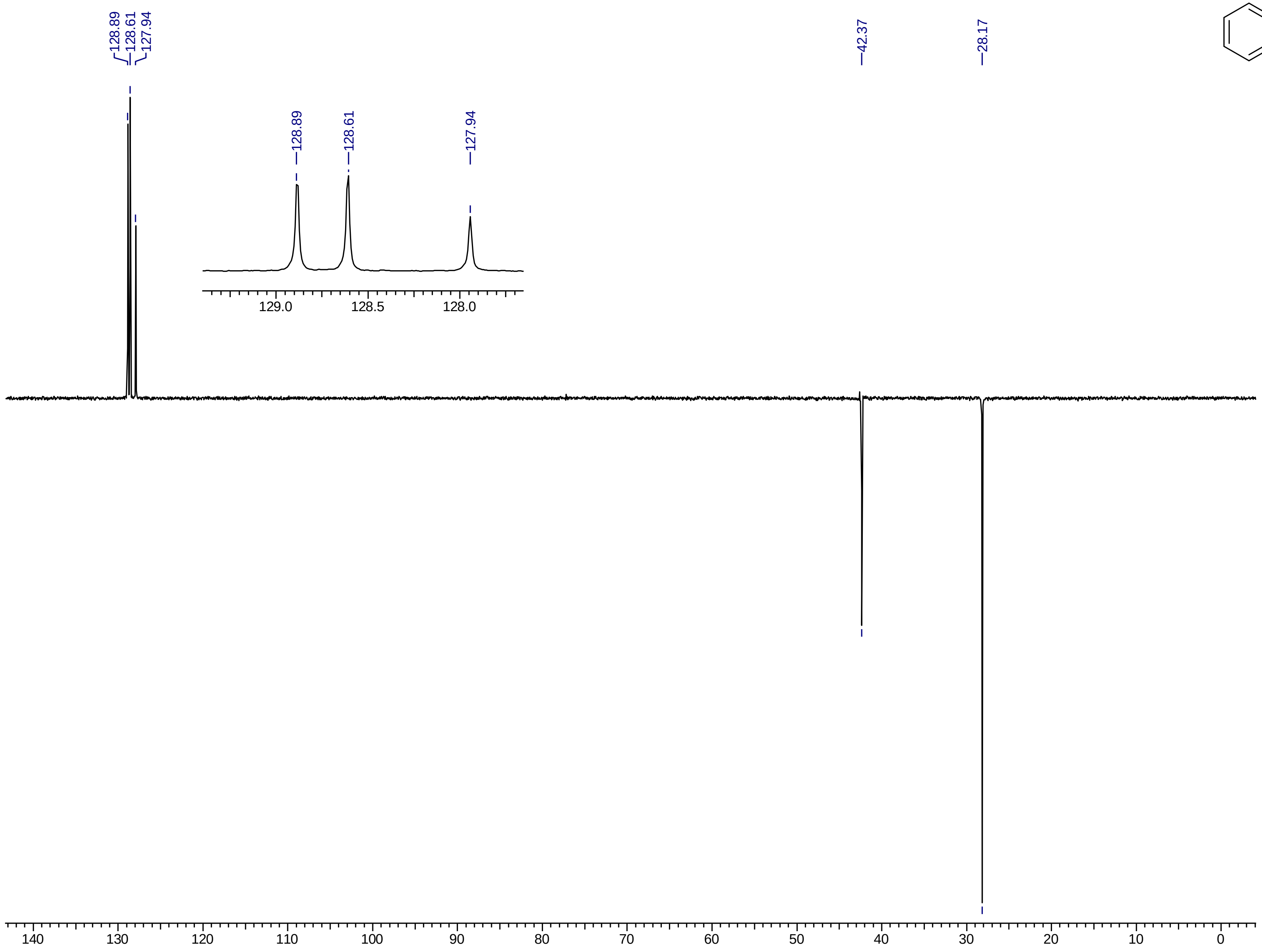

^{1}H NMR, 400 MHz

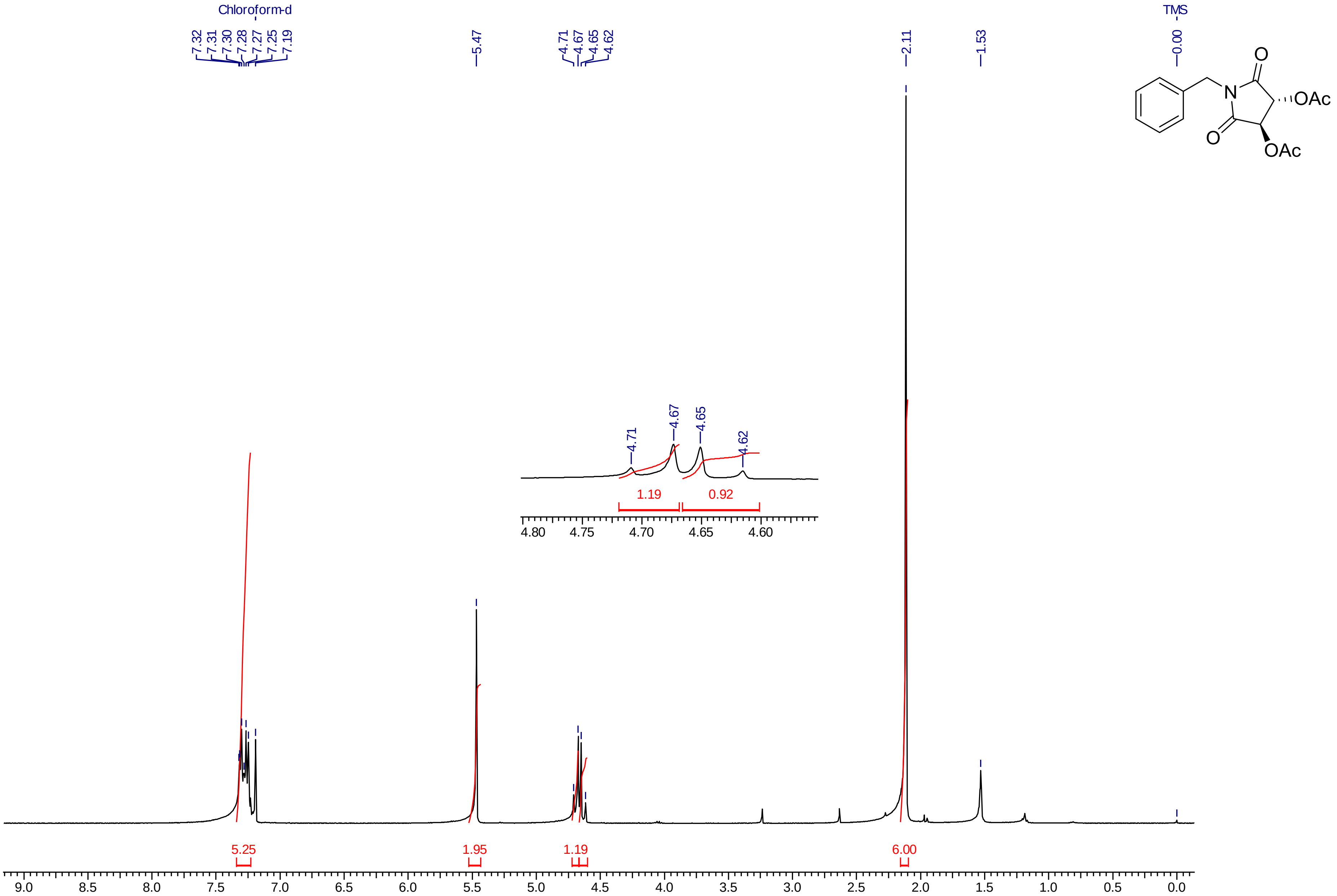

^{13}C NMR, 100 MHz

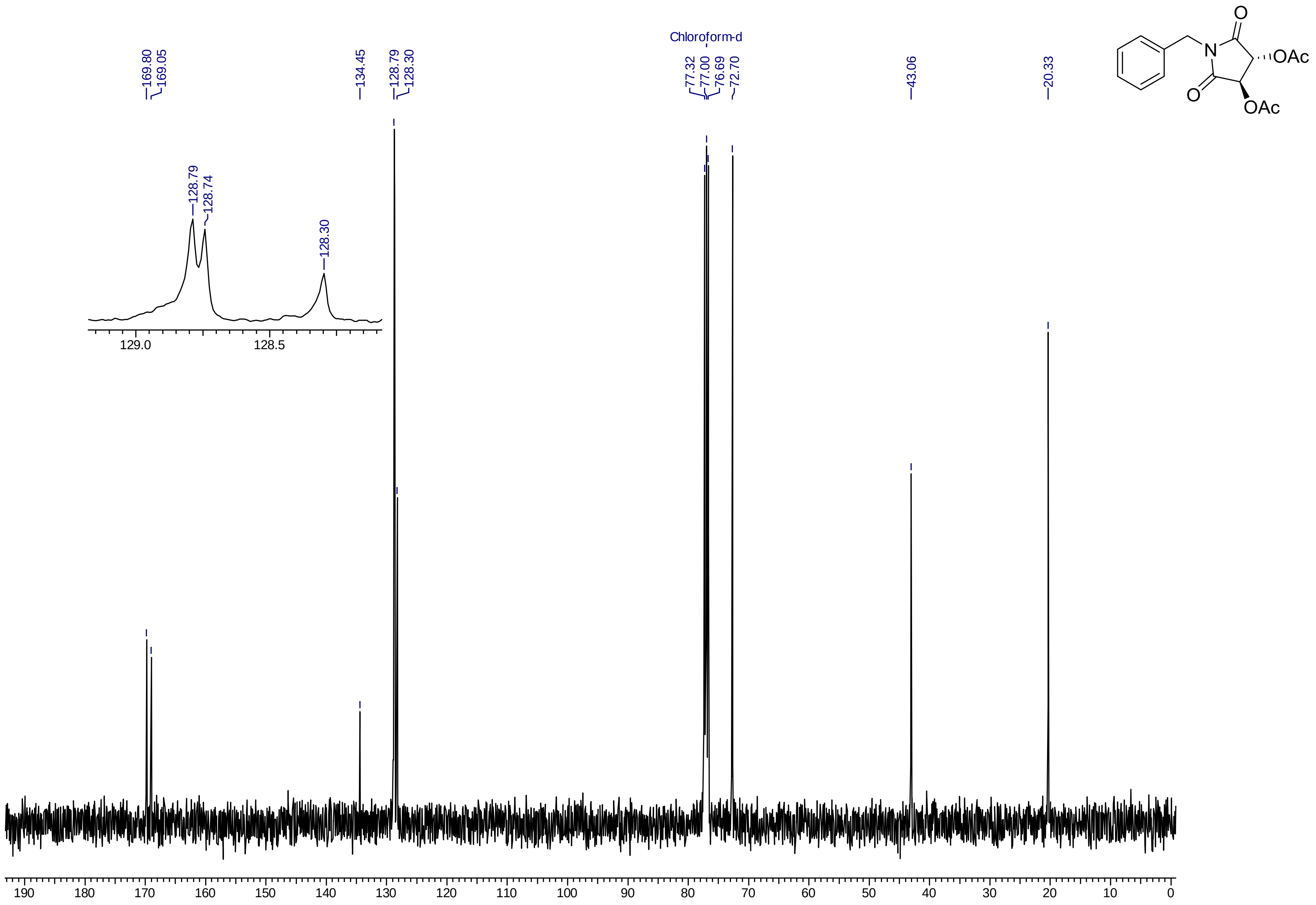

^{13}C NMR, DEPT, 100 MHz

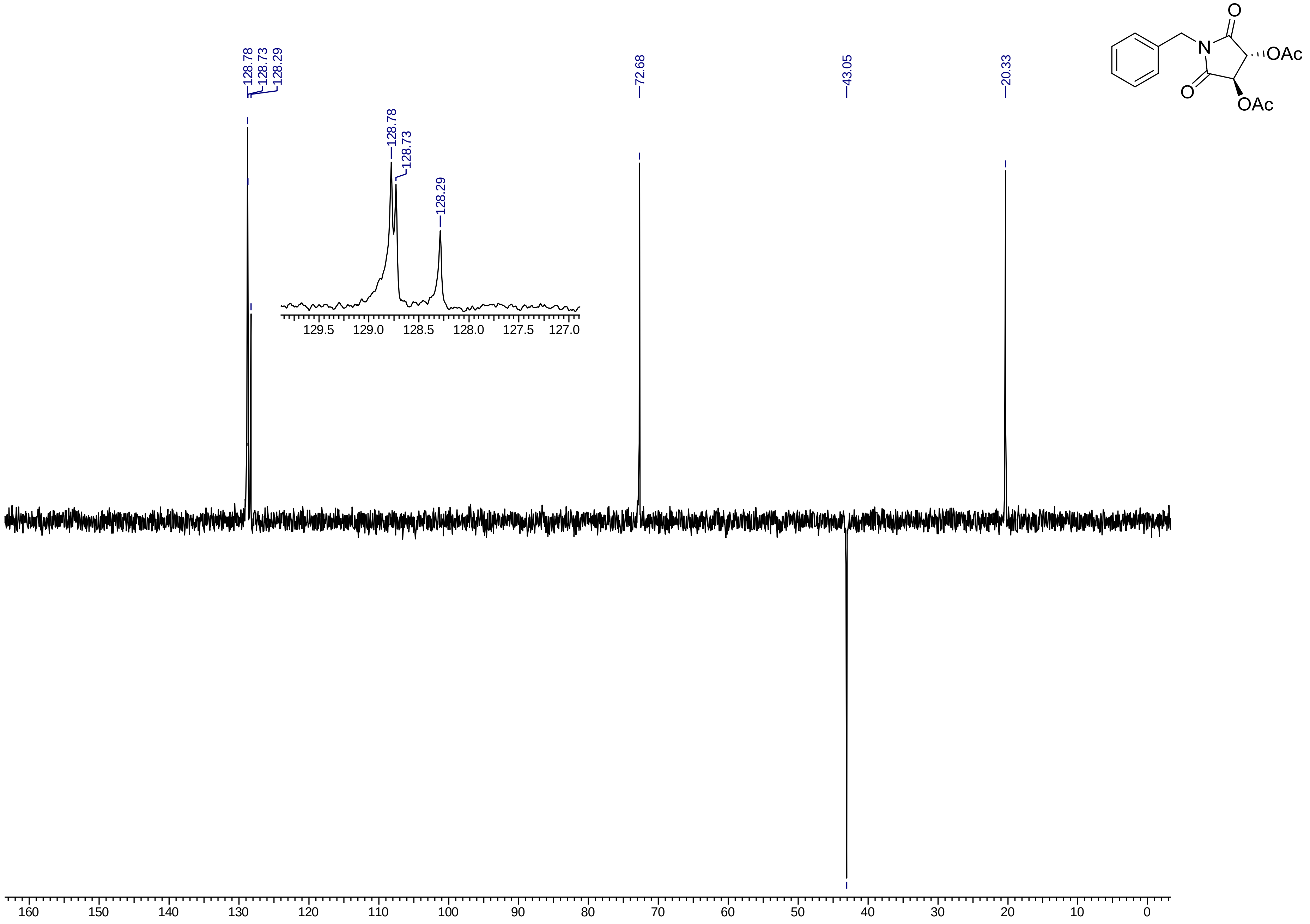

^{1}H NMR, 400 MHz

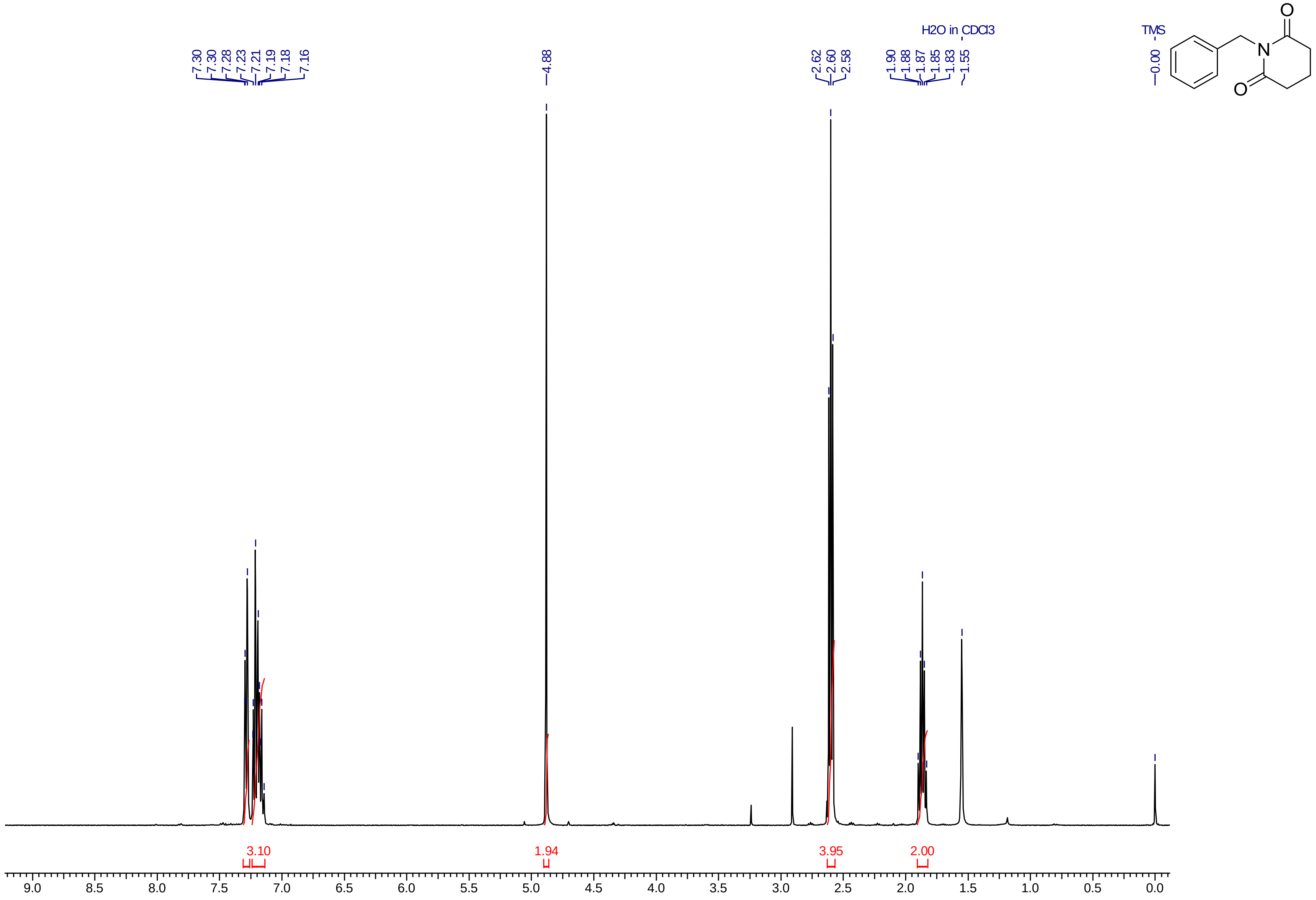

^{13}C NMR, 100 MHz

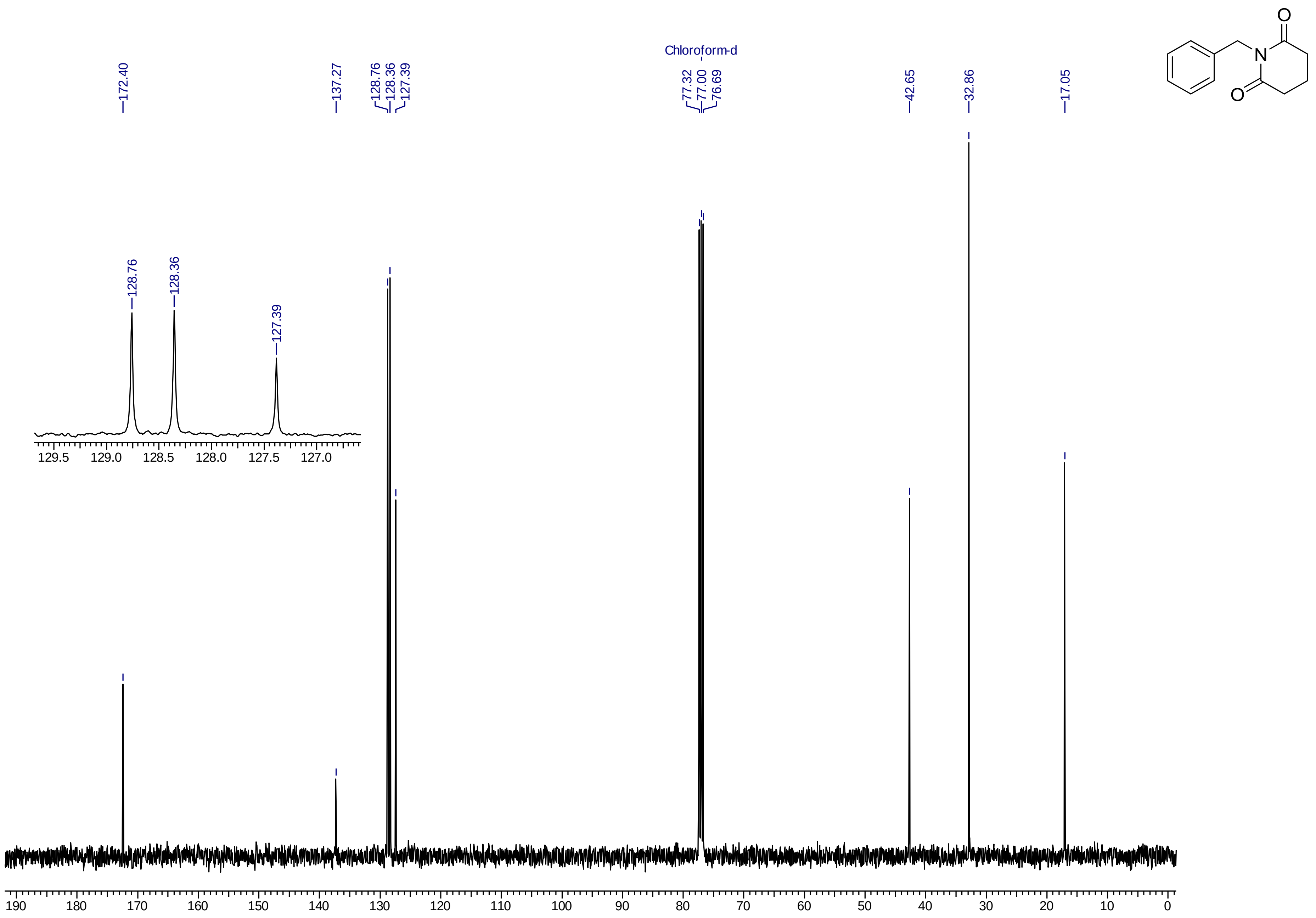

^{13}C NMR, DEPT, 100 MHz

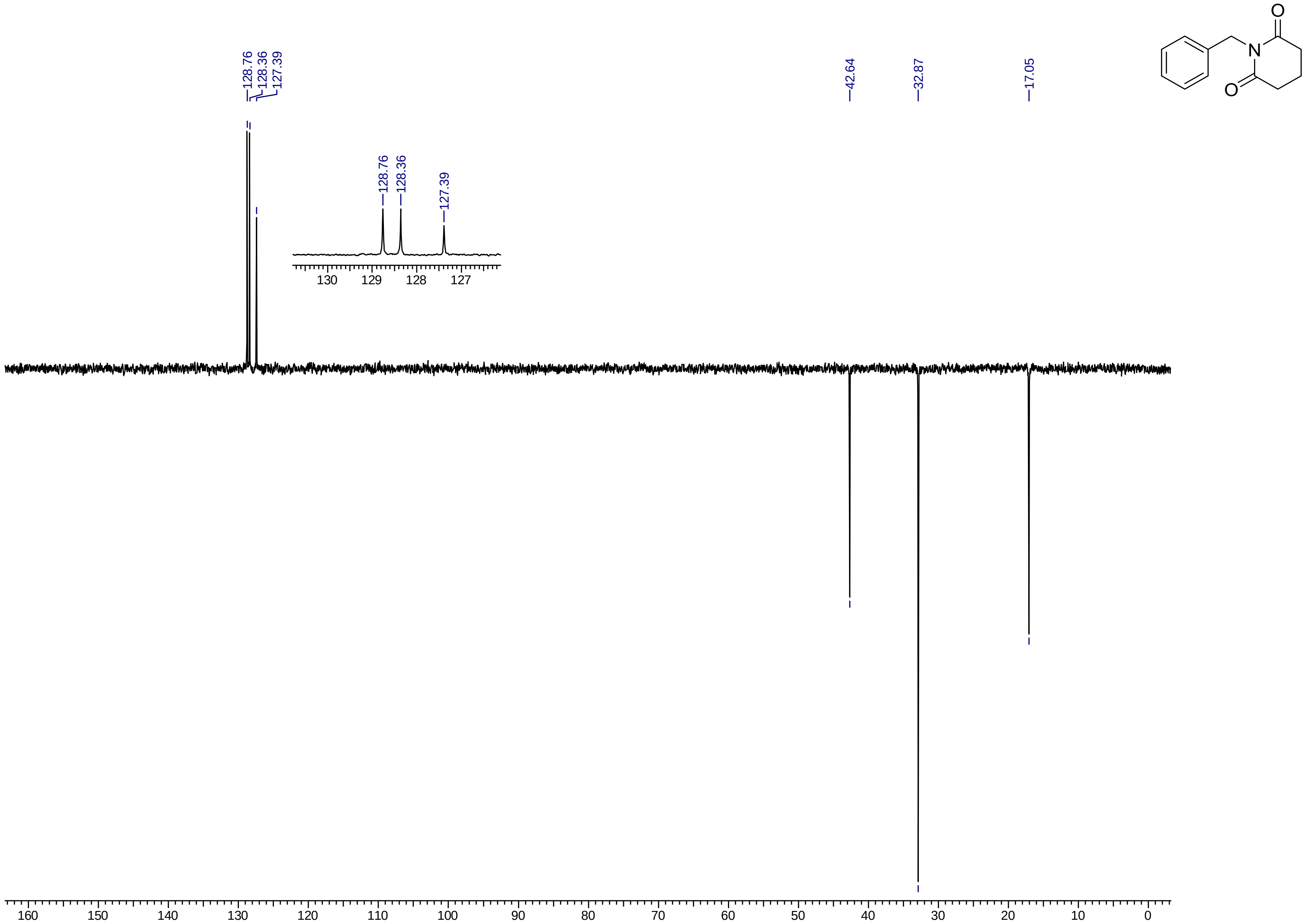

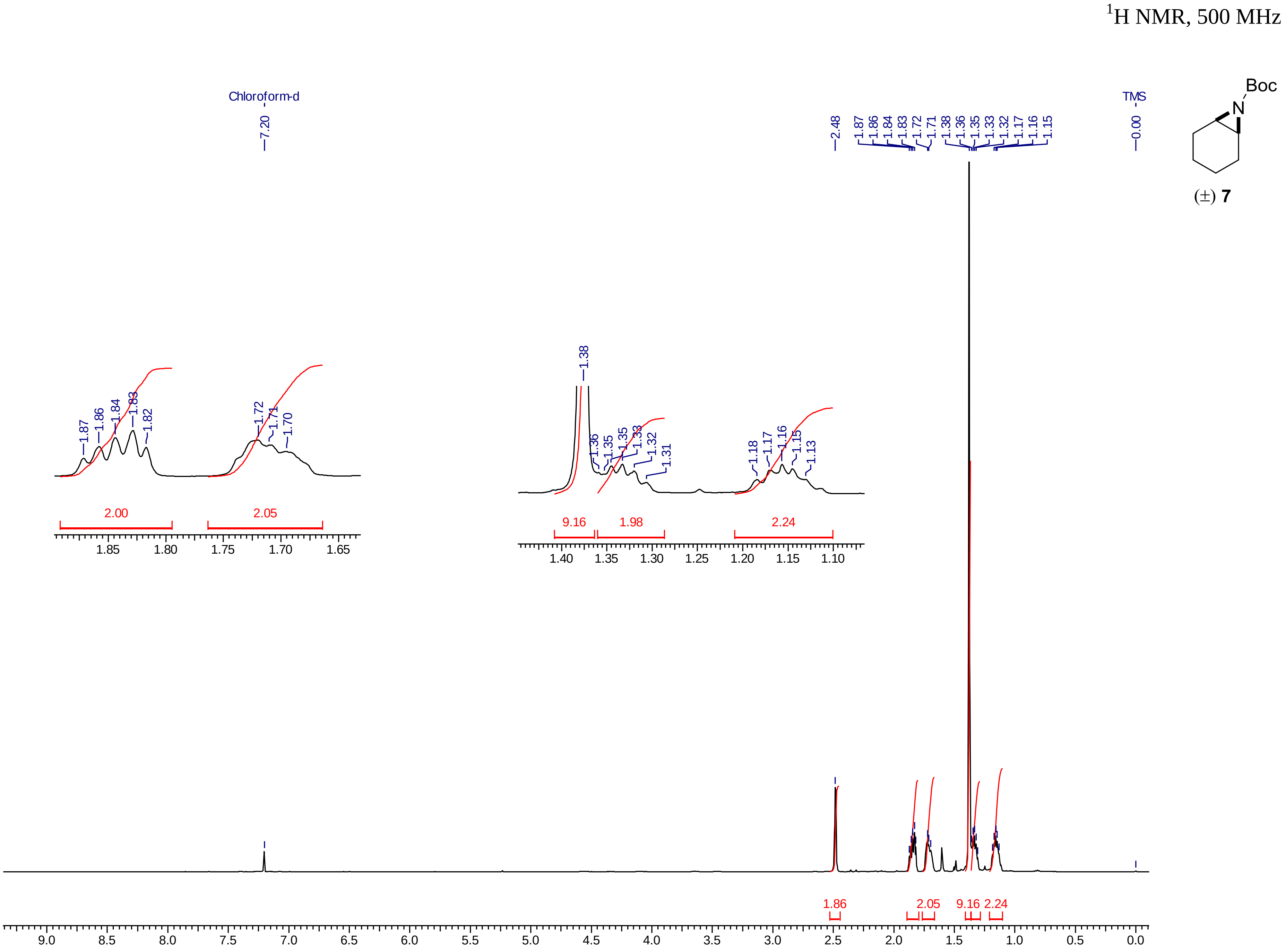
1H NMR, 500 MHz
Chloroform-d
7.20
2.48
1.87
1.86
1.84
1.83
1.72
1.71
1.38
1.36
1.35
1.33
1.32
1.17
1.16
1.15
TMS
0.00
Boc
N
(±) 7
2.00
2.05
9.16
1.98
2.24
1.86

^{13}C NMR, 125 MHz

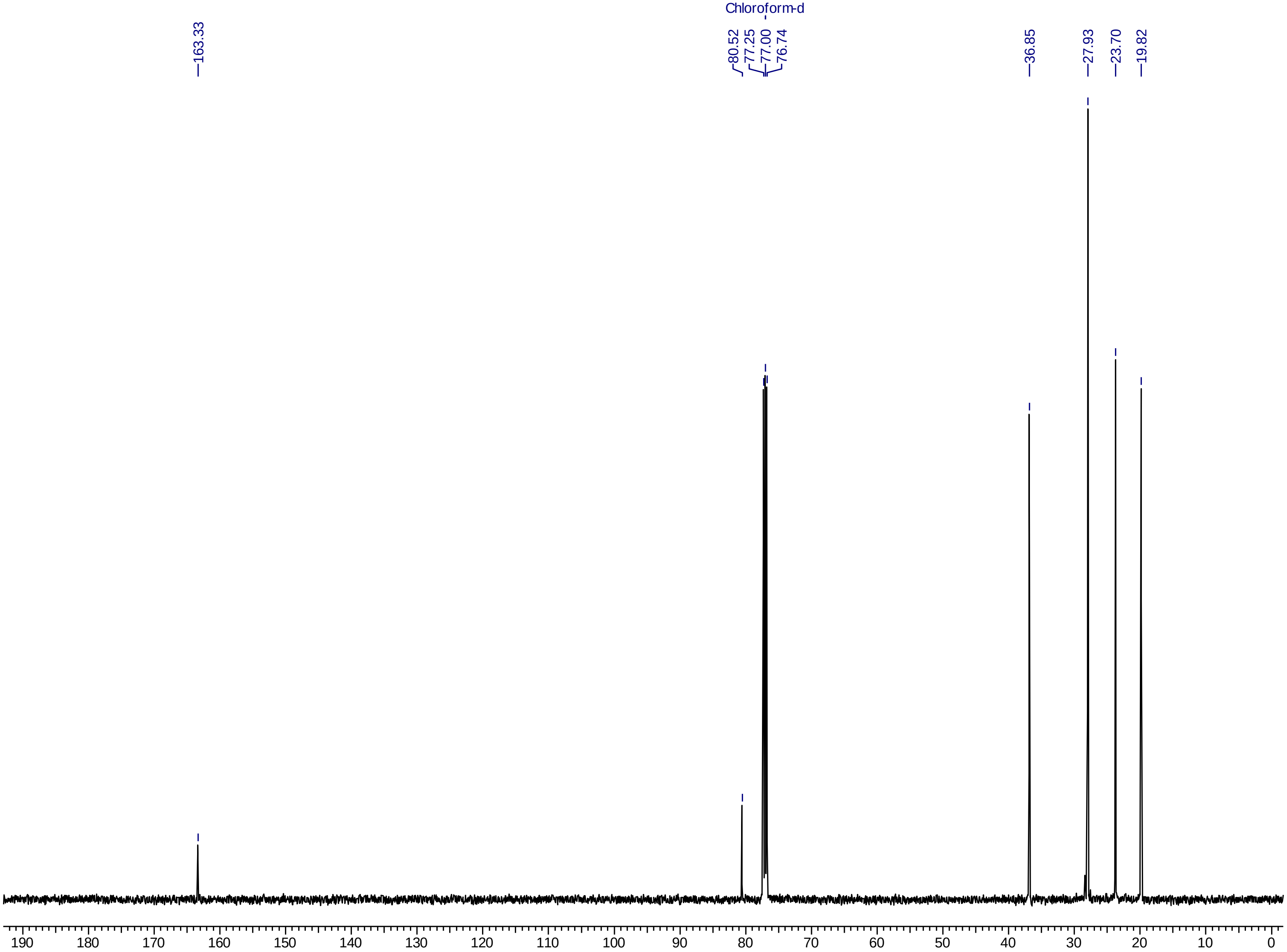

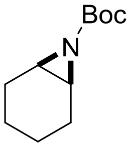

(±) **7**

^{13}C NMR, DEPT, 125 MHz

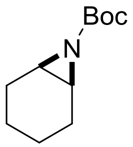

(±) **7**

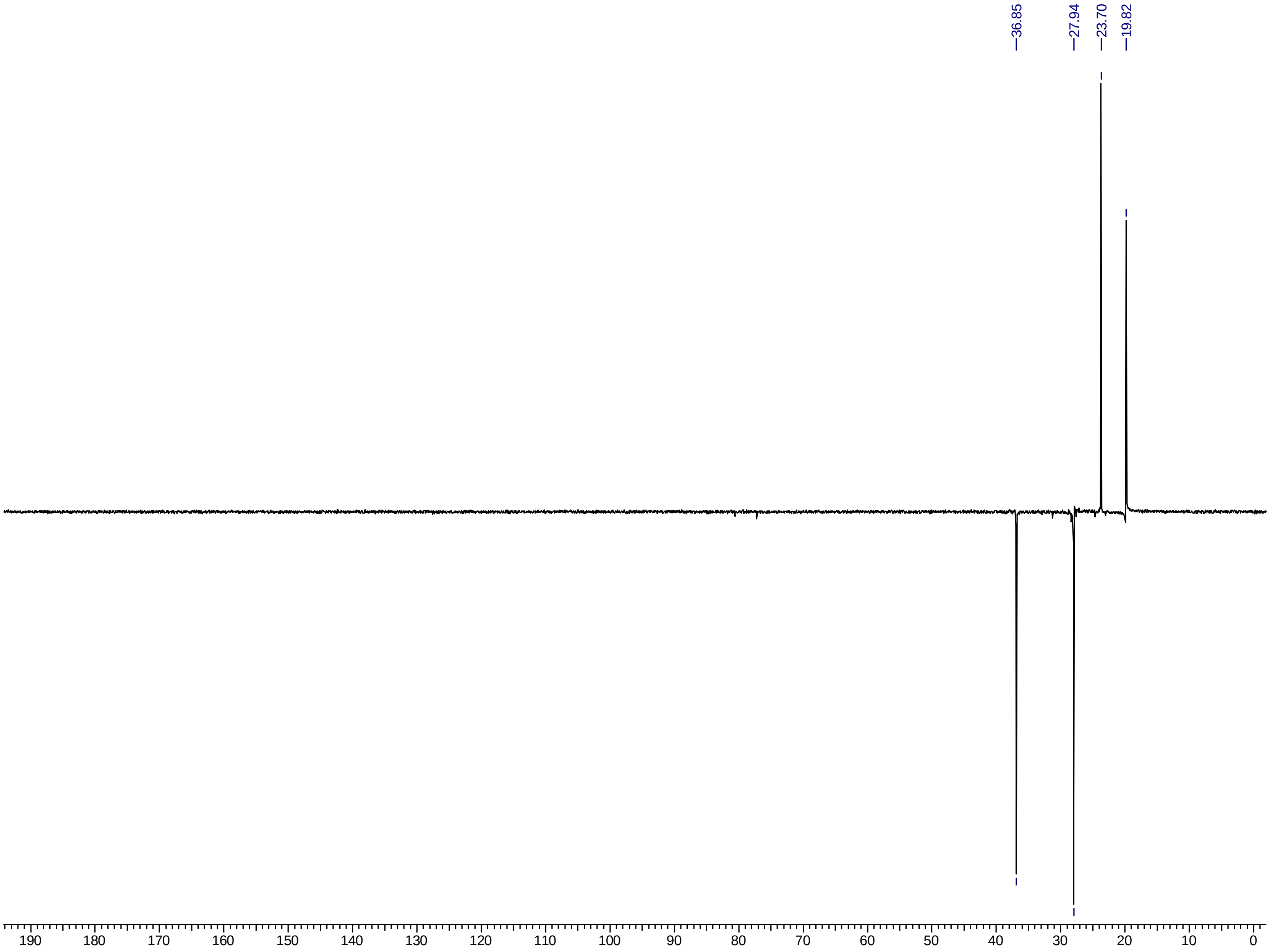

^1H NMR, 400 MHz

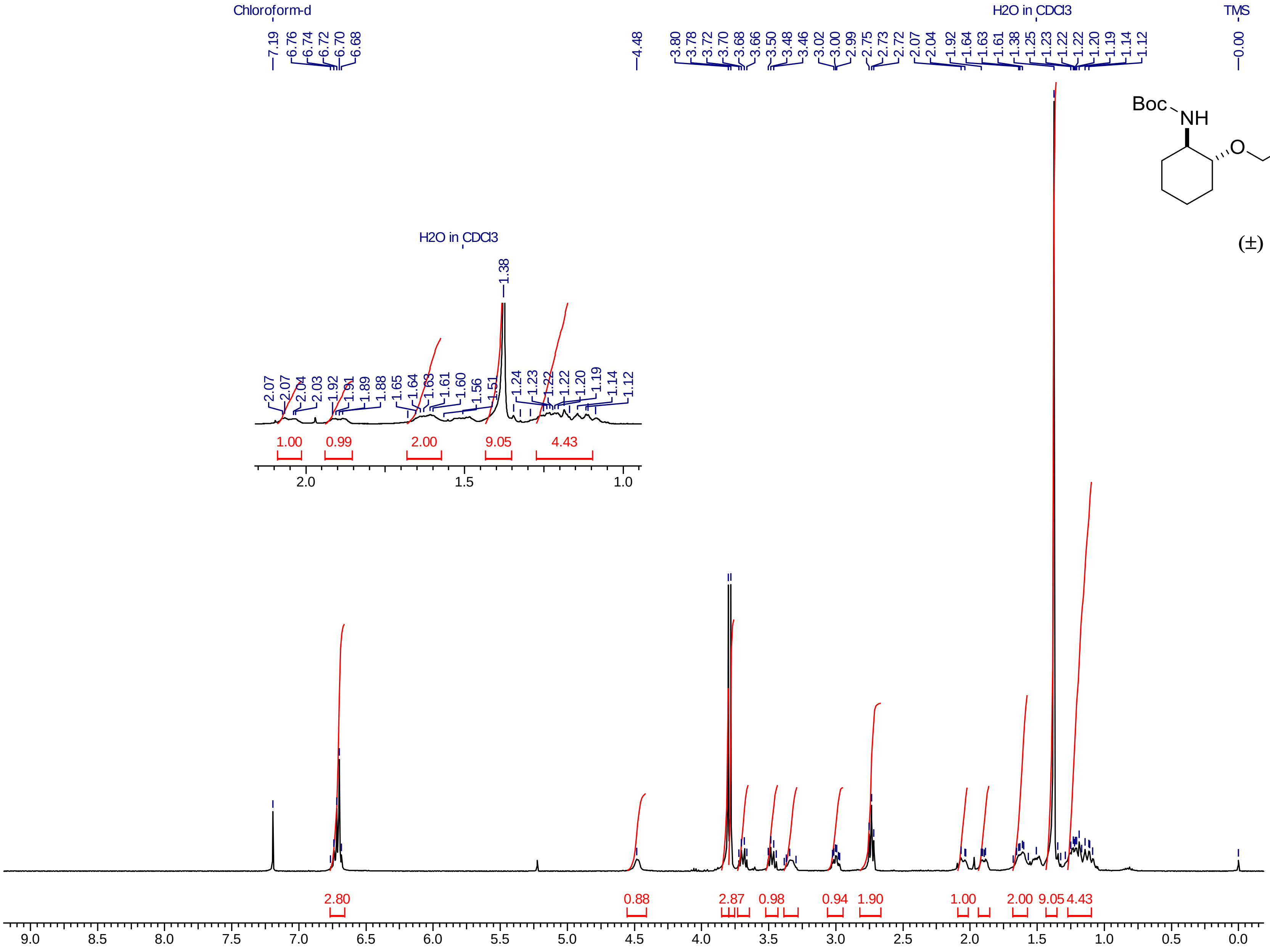

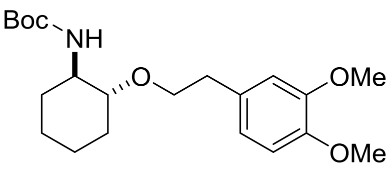

(±) 8

^{13}C NMR, 100 MHz

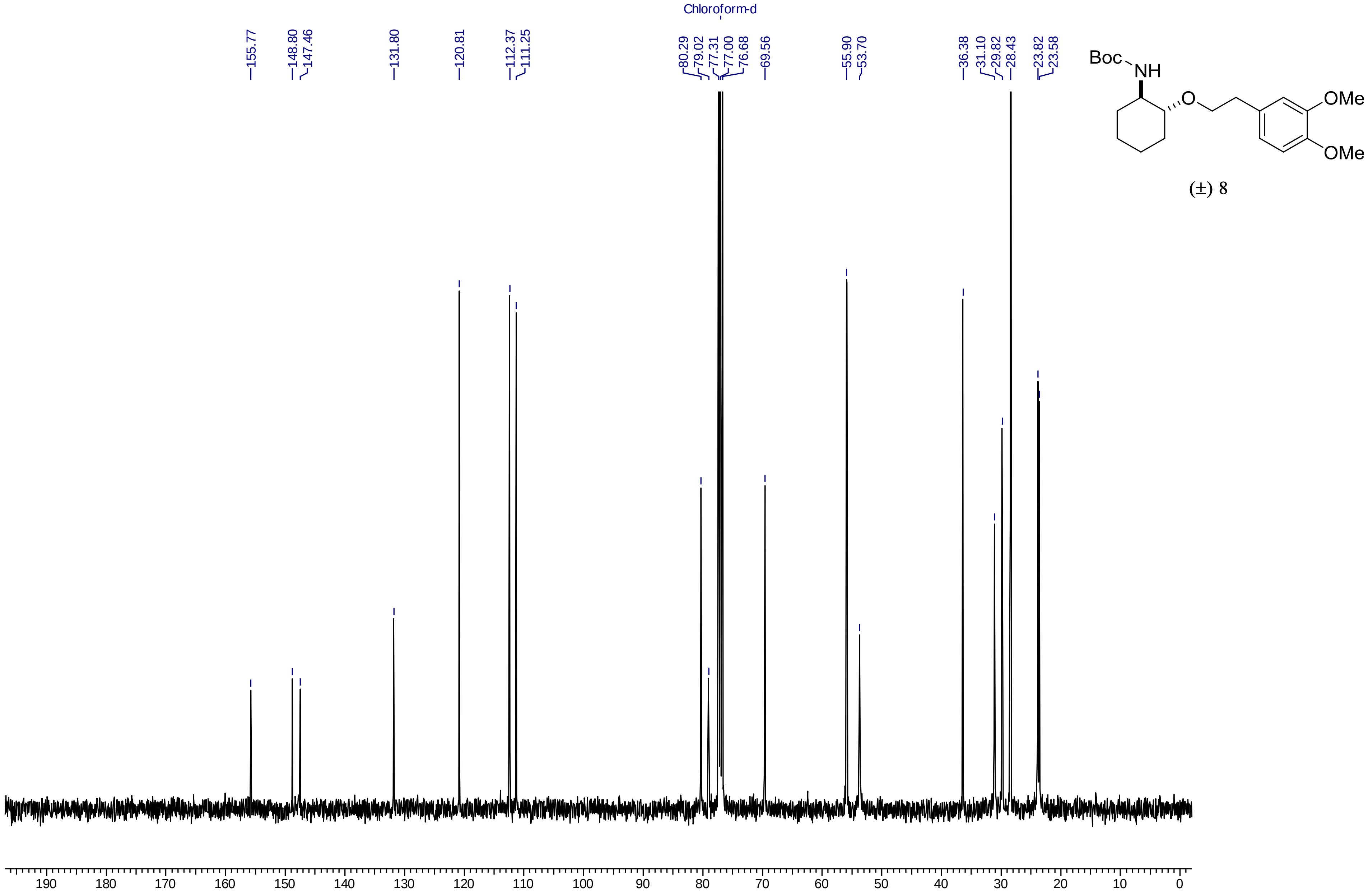

(±) 8

^{13}C NMR, DEPT, 100 MHz

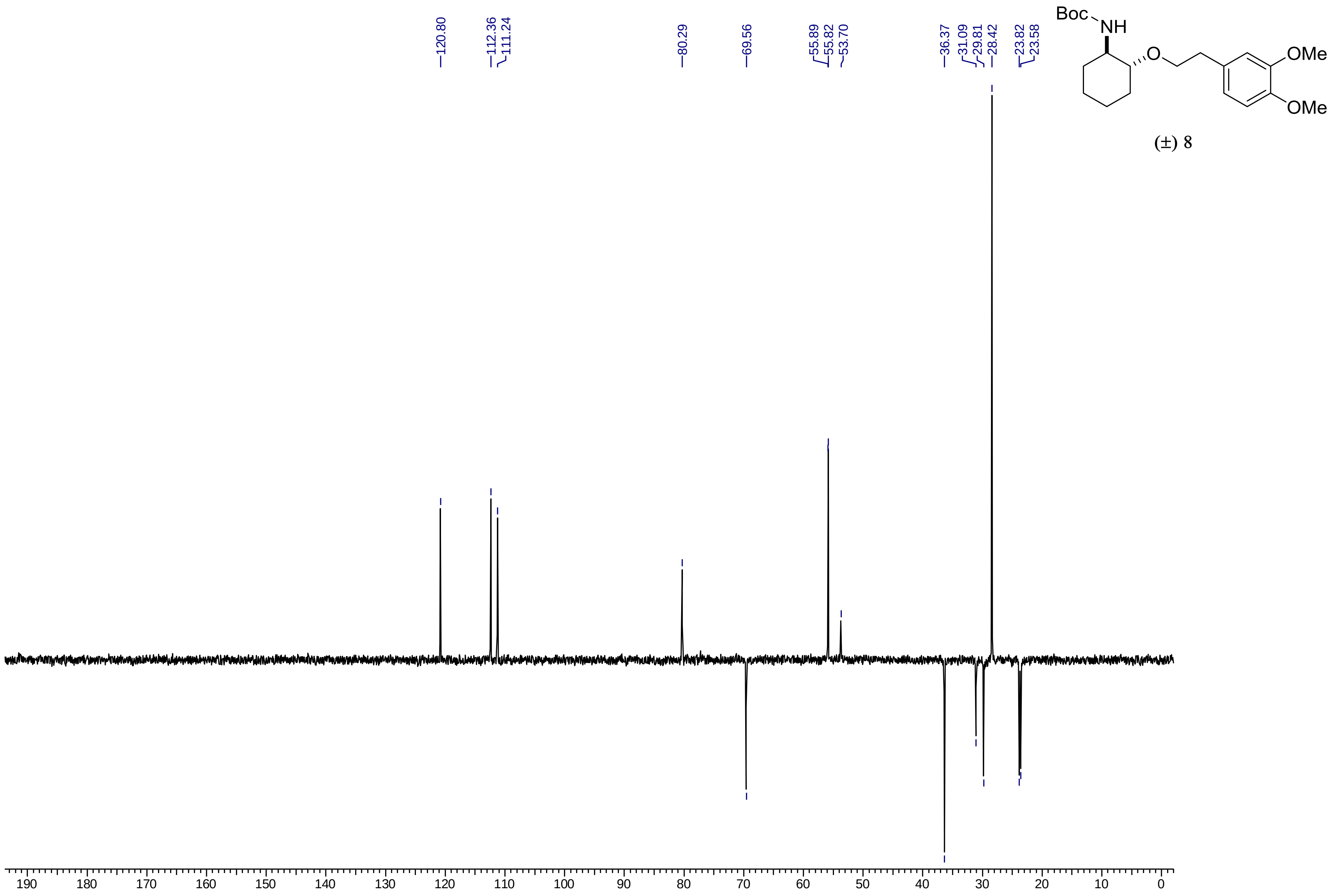

(±) 8

^{1}H NMR, 500 MHz

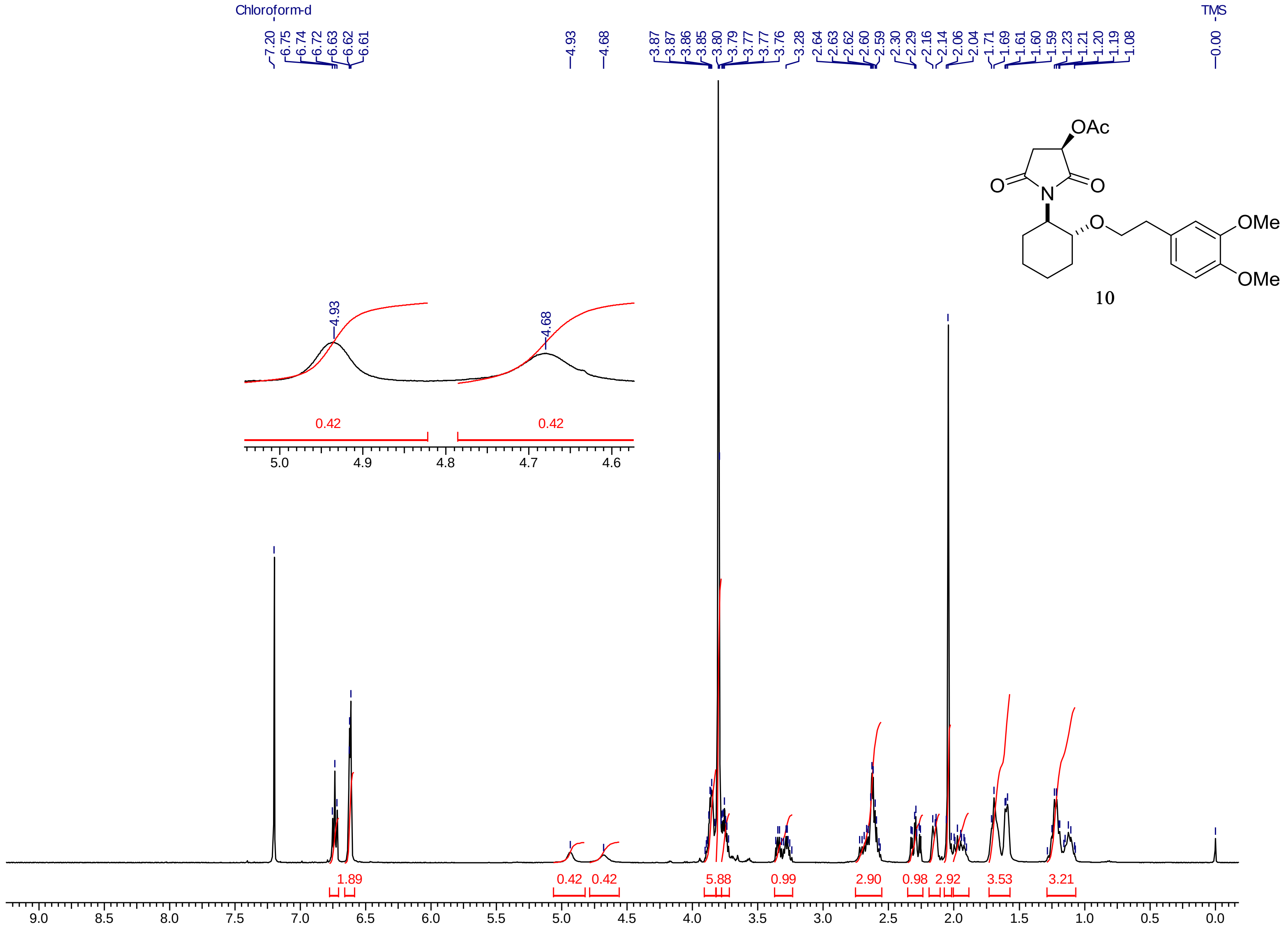

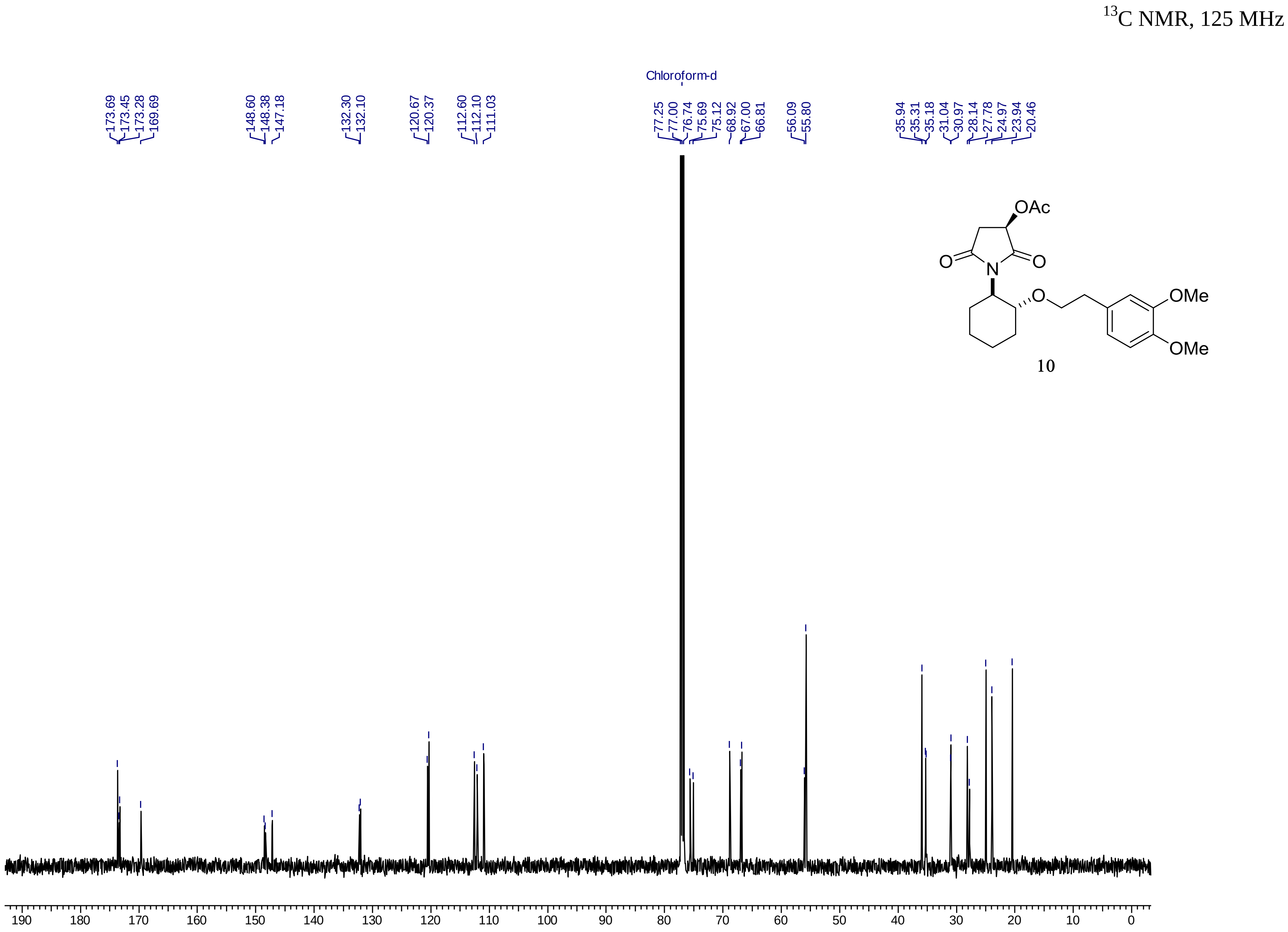

^{13}C NMR, 125 MHz
Chloroform-d
173.69
173.45
173.28
169.69
148.60
148.38
147.18
132.30
132.10
120.67
120.37
112.60
112.10
111.03
77.25
77.00
76.74
75.69
75.12
68.92
67.00
66.81
56.09
55.80
35.94
35.31
35.18
31.04
30.97
28.14
27.78
24.97
23.94
20.46
OAc
O
N
O
O
OMe
OMe
10
190 180 170 160 150 140 130 120 110 100 90 80 70 60 50 40 30 20 10 0

^{13}C NMR, DEPT, 125 MHz

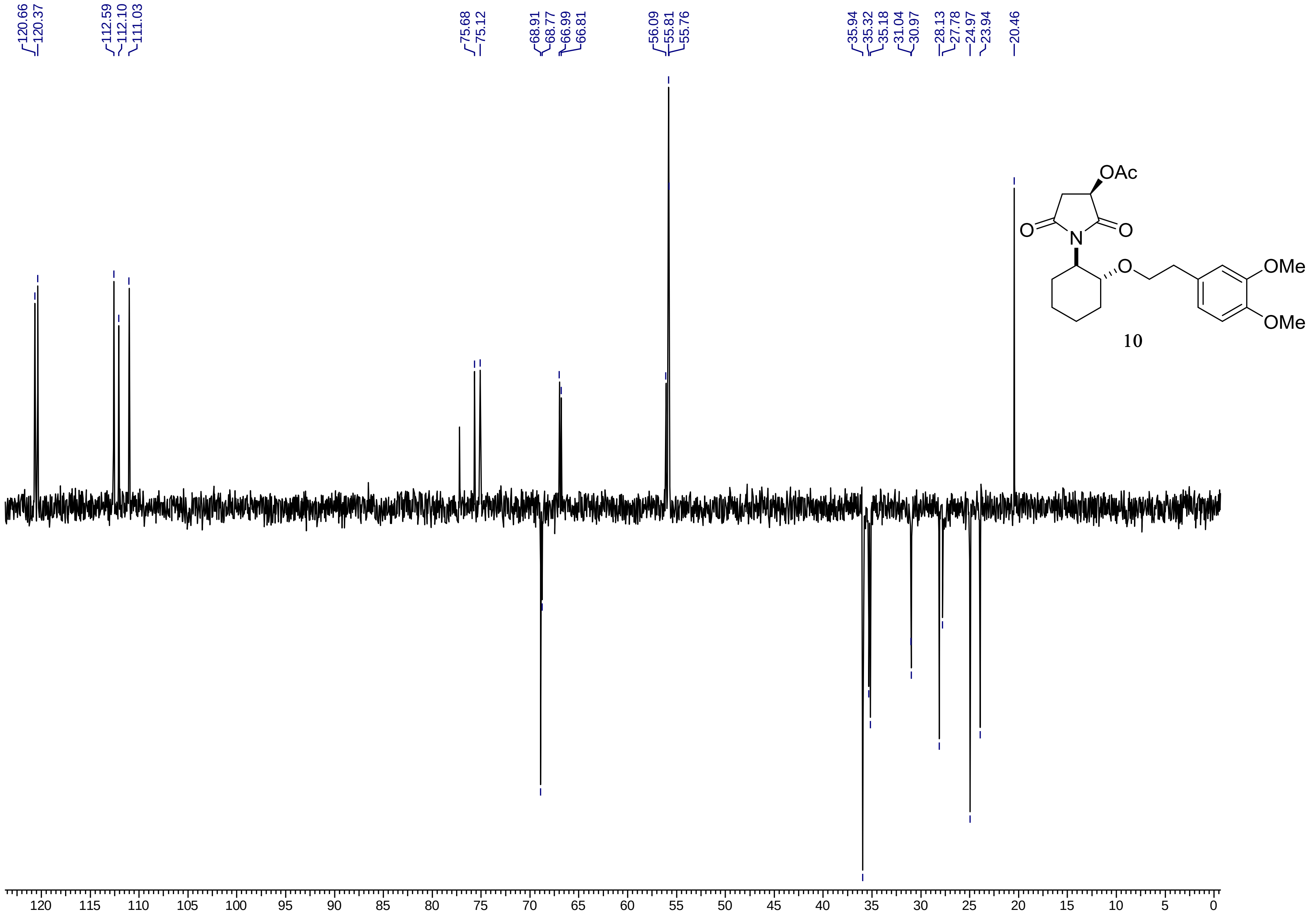

10

^{1}H NMR, 400 MHz

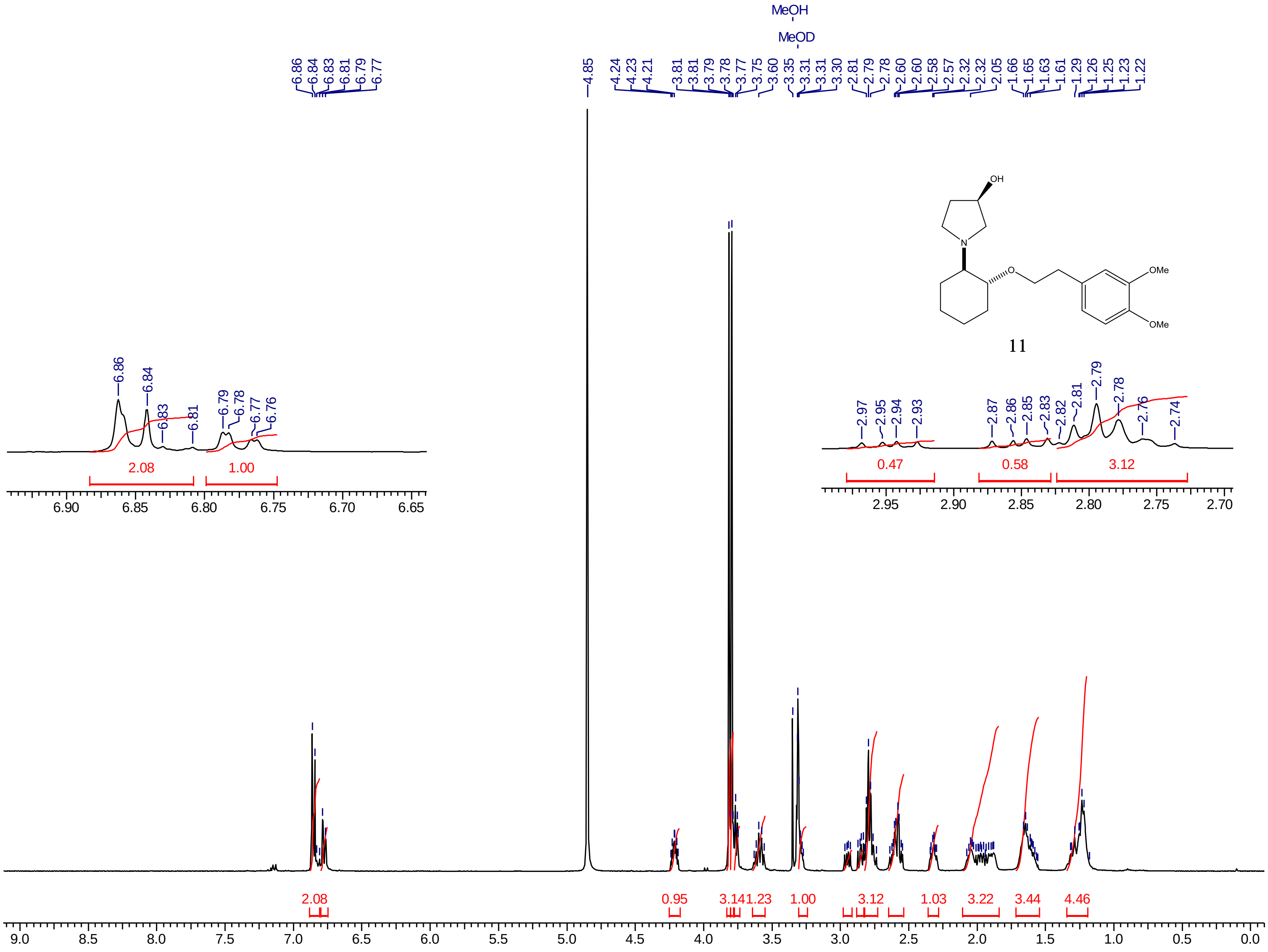

11

^{13}C NMR, 100 MHz

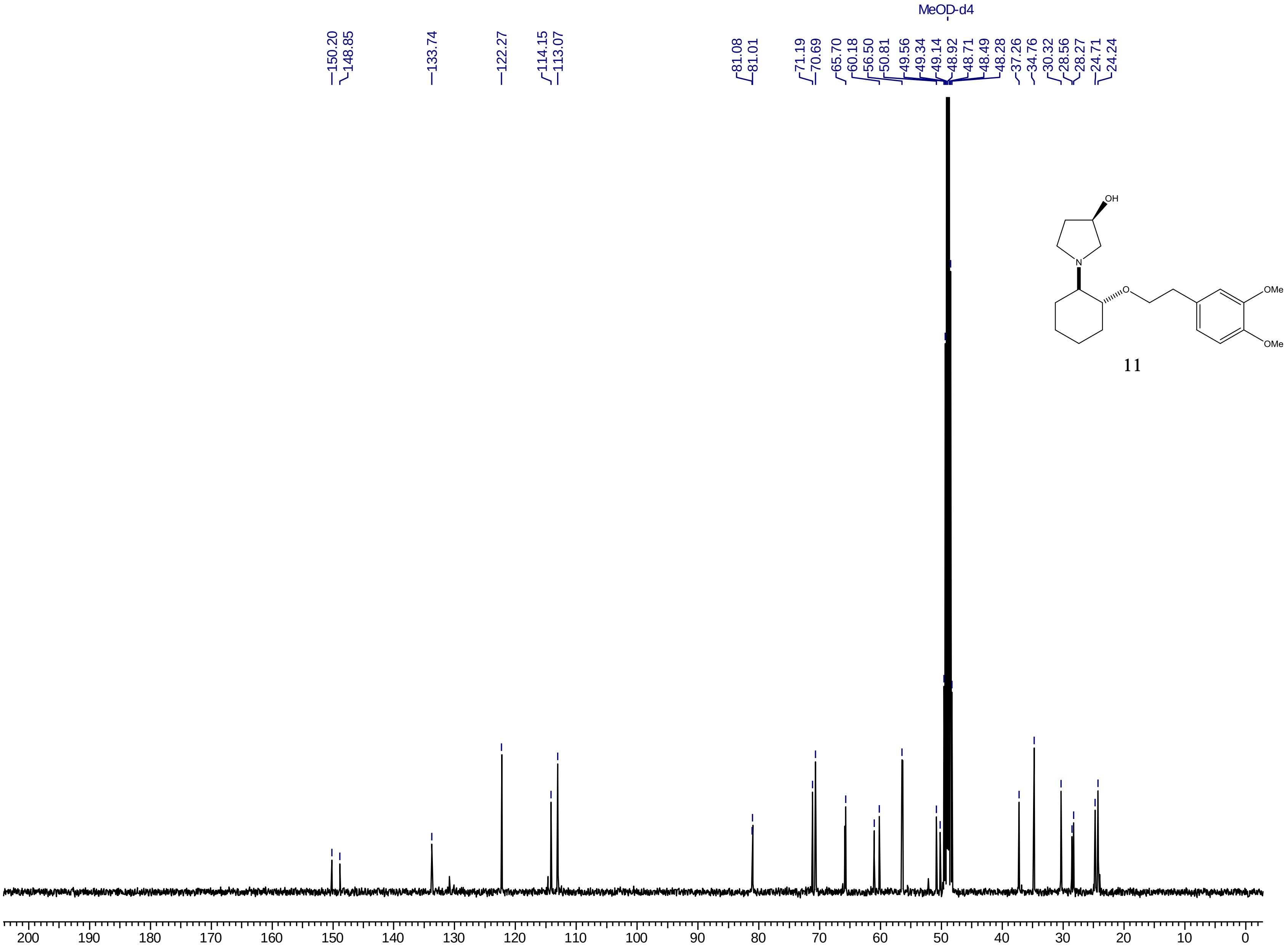

^{13}C NMR, DEPT, 100 MHz

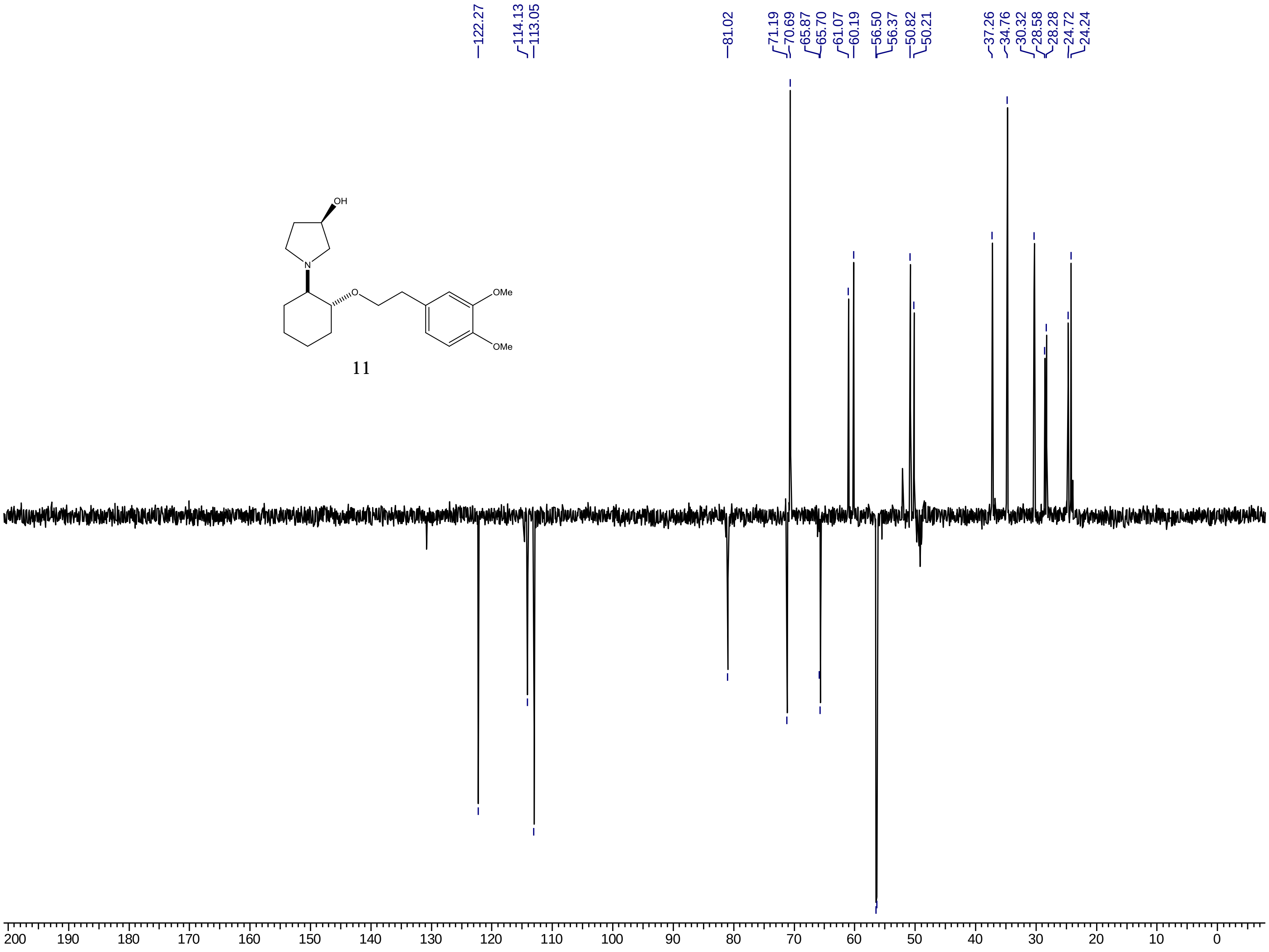

11
